# Atrazine induced epigenetic transgenerational inheritance of disease, lean phenotype and sperm epimutation pathology biomarkers

**DOI:** 10.1371/journal.pone.0184306

**Published:** 2017-09-20

**Authors:** Margaux McBirney, Stephanie E. King, Michelle Pappalardo, Elizabeth Houser, Margaret Unkefer, Eric Nilsson, Ingrid Sadler-Riggleman, Daniel Beck, Paul Winchester, Michael K. Skinner

**Affiliations:** 1 Center for Reproductive Biology, School of Biological Sciences, Washington State University, Pullman, Washington, United States of America; 2 Indiana University, School of Medicine, Department of Pediatrics, Indianapolis, Indiana, United States of America; INIA, SPAIN

## Abstract

Ancestral environmental exposures to a variety of environmental toxicants and other factors have been shown to promote the epigenetic transgenerational inheritance of adult onset disease. The current study examined the potential transgenerational actions of the herbicide atrazine. Atrazine is one of the most commonly used herbicides in the agricultural industry, in particular with corn and soy crops. Outbred gestating female rats were transiently exposed to a vehicle control or atrazine. The F1 generation offspring were bred to generate the F2 generation and then the F2 generation bred to generate the F3 generation. The F1, F2 and F3 generation control and atrazine lineage rats were aged and various pathologies investigated. The male sperm were collected to investigate DNA methylation differences between the control and atrazine lineage sperm. The F1 generation offspring (directly exposed as a fetus) did not develop disease, but weighed less compared to controls. The F2 generation (grand-offspring) was found to have increased frequency of testis disease and mammary tumors in males and females, early onset puberty in males, and decreased body weight in females compared to controls. The transgenerational F3 generation rats were found to have increased frequency of testis disease, early onset puberty in females, behavioral alterations (motor hyperactivity) and a lean phenotype in males and females. The frequency of multiple diseases was significantly higher in the transgenerational F3 generation atrazine lineage males and females. The transgenerational transmission of disease requires germline (egg or sperm) epigenetic alterations. The sperm differential DNA methylation regions (DMRs), termed epimutations, induced by atrazine were identified in the F1, F2 and F3 generations. Gene associations with the DMRs were identified. For the transgenerational F3 generation sperm, unique sets of DMRs (epimutations) were found to be associated with the lean phenotype or testis disease. These DMRs provide potential biomarkers for transgenerational disease. The etiology of disease appears to be in part due to environmentally induced epigenetic transgenerational inheritance, and epigenetic biomarkers may facilitate the diagnosis of the ancestral exposure and disease susceptibility. Observations indicate that although atrazine does not promote disease in the directly exposed F1 generation, it does have the capacity to promote the epigenetic transgenerational inheritance of disease.

## Introduction

Epigenetic transgenerational inheritance is defined as the germline transmission of epigenetic information and phenotypic change across generations in the absence of any continued direct environmental exposure or genetic manipulation [1–3]. Exposure of a gestating female (F0 generation) also exposes the F1 generation fetus and the germline within the fetus which will generate the F2 generation, such that the F3 generation is the first transgenerational generation with no potential exposure [2, 4]. A critical window of exposure for the germline is during fetal gonadal sex determination when epigenetic reprogramming in the primordial germ cell undergoes a DNA methylation erasure followed by remethylation during gonadal development [1]. Environmental exposures during this developmental period appear to promote a permanent alteration in the germline epigenome (DNA methylation) that is protected from epigenetic reprogramming after fertilization, similar to an imprinted gene [5]. This germline epigenetic inheritance will alter the embryonic stem cell epigenome such that all cell types derived will have an altered epigenome and transcriptome, and those somatic cell types sensitive to this altered epigenome and gene expression will be susceptible to developing adult onset disease across generations [6, 7]. Previous studies with a number of environmental toxicants including the fungicide vinclozolin [2, 5], plastic compounds (bisphenol A and phthalates) [8], insect repellent N,N-diethyl-meta-toluamide (DEET) and pesticide permethrin [9], dioxin [10], hydrocarbons (jet fuel JP8) [11], dichlorodiphenyltrichloroethane (DDT) [12], and methoxychlor [13] have been shown to promote the epigenetic transgenerational inheritance of adult onset disease and sperm epimutations [14]. Interestingly, the transgenerational epigenetic alterations (epimutations) in sperm appear to be exposure-specific and may be useful as biomarkers of ancestral toxicant exposure and susceptibility to transgenerational adult onset disease [14]. The current study was designed to examine the potential transgenerational impacts of atrazine exposure.

Atrazine is a widely-used herbicide in agriculture, especially for corn and soy, that frequently contaminates ground, surface and drinking water [15], S1 Fig. The compound atrazine (2 chloro-4-ethylamino-6-isopropyl-amino- s-triazine) is a triazine herbicide that acts as an endocrine disrupter and can cause demasculinization and feminization [16]. Atrazine can inhibit estrogen-mediated signaling responses [17]. Studies have also found that high doses of prenatal atrazine can promote overt toxicity and cause reduced weight in exposed progeny [18]. One study found that very high doses of atrazine (100 mg/kg per day) produced significantly decreased weight in the rat pups, but they caught up with the controls by post-natal day 35 [19]. Three separate studies also observed maternal weight loss in atrazine treated rats, two studies at a much higher dosage (100 mg/kg per day and 500 ppm) and the third study at a dosage as low as 25 mg/kg per day [20–22]. In three of five studies, which examined Sprague-Dawley rats for two years while they were exposed to varying doses of atrazine orally, the rats had a significant increase in tumor rates at the higher doses (400, 500 and 1,000 ppm) [23]. Another study concluded that atrazine acts to inhibit the cell-mediated, humoral, and non-specific immune functions of mice after two weeks of gavage administration at levels of 87.5 and 175 mg/kg per day [24]. Atrazine exposure has also been shown to alter testis function [16, 25, 26] and behavior [27, 28] in rodent studies. In 2003 the European Union banned the use of atrazine, based on its prevalent contamination of water, but in the same year in the United States the Environmental Protection Agency (EPA) permitted its continued use. Levels of atrazine during peak agricultural use are generally at about 5 μg/L in affected reservoirs, although they can reach levels of 20 μg/L [15]. A United States Geological Survey (USGS) study found that water in streams and rivers surrounding agricultural areas can have as much as 144 parts per billion of atrazine, and runoff from the fields can reach 4,000 parts per billion [29], compared to the maximum contaminant level of 3 parts per billion allowed by the EPA [30]. In the Midwest USA drinking water levels of atrazine reach 59 ppm in some public water systems. The regional distribution of atrazine use in the USA and associated crops for 2014 is shown in S1 Fig, (https://water.usgs.gov/nawqa/pnsp/usage/maps/show_map.php?year=2014&map=ATRAZINE&hilo=L&disp=Atrazine). The current study used a daily exposure dose of 25 mg/kg for seven days during fetal development.

Environmental toxicants have been shown to promote the transgenerational inheritance of a number of different pathologies, diseases and phenotypic abnormalities. Vinclozolin, an anti-androgenic compound, induces transgenerational prostate disease, kidney disease, immune system abnormalities, testis abnormalities, and tumor development in rats [31]. Ancestral DDT exposure has been shown to promote testis disease, polycystic ovarian disease, immune abnormalities, kidney disease and obesity [12]. Pesticide and insect repellent mixtures (permethrin and DEET) were found to induce pubertal abnormalities, testis, and ovarian disease [9]. Plastic derived (bisphenol A, BPA and phthalates) endocrine disruptor compounds were also found to induce pubertal abnormalities, testis disease, obesity, and ovarian disease as well as changes in social recognition and activity [8, 32–34]. As discussed, the transgenerational transmission of epigenetic change from an exposed gestating female requires assessment of the F3 generation. The pathologies observed in the directly exposed F1 and F2 generation are important to assess and compare with the F3 generation to determine the differences between the direct exposure and transgenerational pathologies. The current study examines the pathology of the F1, F2 and F3 generations to identify those pathologies that become transgenerational.

The current study was designed to investigate the potential that the agricultural herbicide atrazine may promote the epigenetic transgenerational inheritance of disease in both male and female rats. Following the transient exposure of an F0 generation gestating female outbred rat during fetal gonadal sex determination, subsequent generations were analyzed. This study used a dose of 25 mg/kg body weight for atrazine (4% of rat oral LD50) [35] with an intraperitoneal administration to gestating rats. Direct exposure toxic effects of atrazine were observed in the induction of reduced weights in the F1 generation as previously observed [18]. The current study was not designed as a risk assessment study, but to simply investigate the potential that atrazine may promote transgenerational abnormalities and disease. Tissues previously showing transgenerational diseases were investigated including the testis, prostate, kidney, and ovary. In addition, tumor development, abnormal puberty onset and metabolic phenotypes were assessed. Pathology was evaluated in 12-month-old F1, F2 and F3 generation control and atrazine lineage rats. Another aspect of the study tested for behavioral changes in the F3 generation with the use of open field and elevated plus mazes. Results showed a significant increase in testes disease in the atrazine lineage males, and an increase in tumor rates in the F2 generation, as well as a significant increase in a lean phenotype frequency in both males and females in the F3 generation. Alterations in differential DNA methylation regions (DMRs) in the atrazine lineage F1, F2 and F3 generation sperm (epimutations) were observed and associated with adult onset disease phenotype in the F3 generation atrazine lineage sperm. Observations suggest pathology-specific epigenetic alterations may be useful as biomarkers (i.e. diagnostics) to identify transgenerational adult-onset disease susceptibility.

## Results

### Pathology analysis

The transgenerational actions of control vehicle (DMSO) and atrazine (25mg/kg body weight) treatments administered to female rats (F0 generation) during days 8 to 14 of gestation were investigated. The dose of atrazine used is a putative high dose environmental exposure [15, 29]. The F1 generation (direct fetal exposure), F2 generation (direct germline exposure) and F3 generation (transgenerational) rats of control and atrazine lineages were aged to 1 year and euthanized for analysis. No inbreeding (sibling or cousin crosses) was performed to maintain the outbred nature of the lines of rats [2]. The testis, prostate, kidney and ovary were collected and examined for histopathologies. To assess if there was any direct fetal exposure toxicity to atrazine, the F1 generation litter sizes, sex ratios and weaning body weights were measured. No effect was observed on litter size or sex ratio (p>0.05) for any generation. The weaning body weights of the F1 generation were also not directly affected. Therefore, negligible overt toxicity to atrazine was observed in the direct in utero exposed F1 generation lineages. As the animals were aged to 1 year there was a reduced weight or lean phenotype in the males and females in the F1 generation atrazine lineage animals, so some direct exposure toxicity (weight loss) is observed. However, no overt toxicity or pathology was observed in the F0 generation exposed females after monitoring for 3–6 months following exposure.

**Fig 1:**
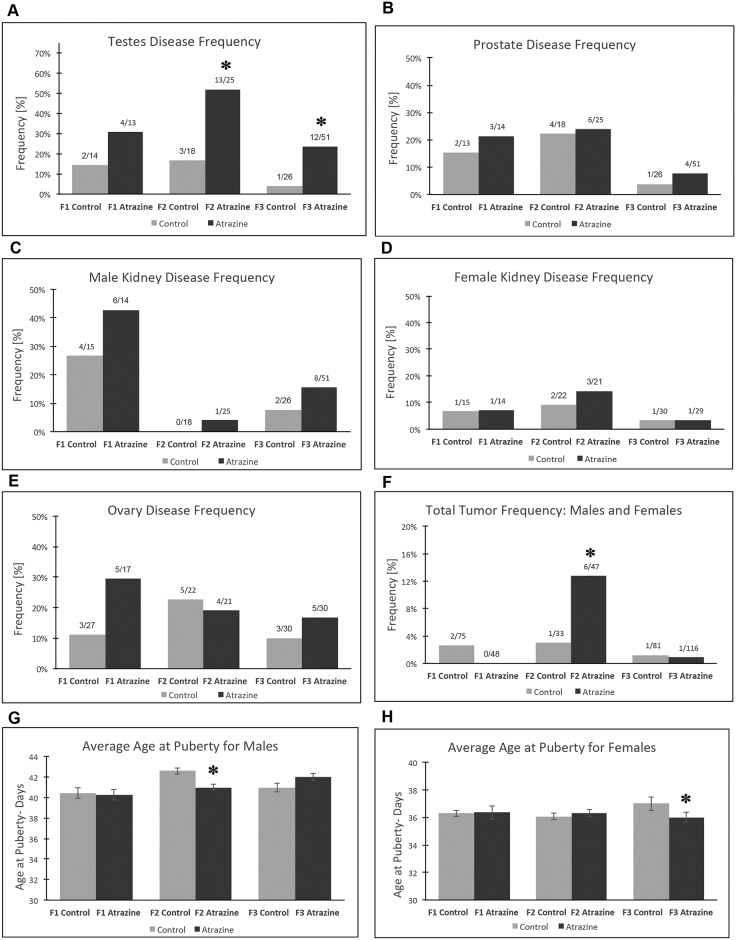
Pathology analysis in F1, F2 and F3 generation control and atrazine linage 1 yr old rats. **(A)** testis disease frequency, **(B)** prostate disease frequency, **(C)** male kidney disease frequency, **(D)** female kidney disease frequency, **(E)** ovary disease frequency, **(F)** total tumor frequency: males and females, **(G)** male pubertal onset age, and **(H)** female pubertal onset age. The pathology number ratio with total animal number is listed for each bar graph (A-F), or mean ±SEM (G-H), presented with (*) indicating a statistical difference p<0.05 in comparison with control lineage animals.

The histopathology analysis was based on examination of stained paraffin sections of isolated tissues, as previously described in the Methods [12]. Testis disease was characterized by the presence of histopathologies including azoospermia, atretic seminiferous tubules, presence of vacuoles in basal regions of seminiferous tubules, sloughed germ cells in the lumen of seminiferous tubules, and lack of seminiferous tubal lumen [2, 31, 36], S2 Fig. Spermatogonial cell apoptosis was assessed as described [2, 31], and this has previously been shown to associate with reduced sperm numbers and motility [36]. The frequency or incidence of testis disease was similar between the control and atrazine lineage F1 generation animals at one year of age, Fig 1. In contrast, the frequency of testis disease was increased in the F2 and F3 generation atrazine lineage compared to control, Fig 1A. Therefore, a transgenerational disease (F3 generation) observed was testis disease in approximately 25% of the atrazine lineage males (a five-fold increase in disease risk).

The frequency of prostate disease was not found to be different between the control versus atrazine lineage F1, F2 or F3 generation males, Fig 1B. Prostate disease was characterized by atrophic or hyperplastic prostate glandular epithelium as previously described [37]. The frequency of kidney disease was also not found to be different between control and atrazine lineages in F1, F2 or F3 generation males or females, Fig 1C and 1D. Kidney disease was characterized by the presence of an increased number of proteinaceous fluid filled cysts, reduction in size of glomeruli and thickening of Bowman’s capsules as previously described [14, 31]. The frequency of ovarian disease was not found to be different between the control versus atrazine lineage F1, F2 or F3 generation females, Fig 1E. Ovarian disease was characterized by the development of polycystic ovaries with an increase in the number of small and large cysts as previously described [38]. In addition, follicle counts were performed to determine any changes in the primordial follicle pool size as previously described [38, 39]. No significant (p>0.22) alteration in ovarian disease was observed, Fig 1E. This was surprising since this was the most frequent disease in females with other environmental toxicant induced transgenerational disease. Tumor development was also monitored in males and females and found to increase in the F2 generation atrazine lineage, but not the F1 or F3 generation atrazine lineages, Fig 1F. The most predominant tumors developed in the male or female were mammary tumors as previously described [12, 31], but other tumors observed included brain and skin tumors. Tumor histopathology analysis generally identified adenomas or sarcomas of the tissues. The pubertal analysis identified early pubertal onset in males in the F2 generation atrazine lineage and early pubertal onset in females in the F3 generation atrazine lineage, Fig 1G & 1H.

**Fig 2:**
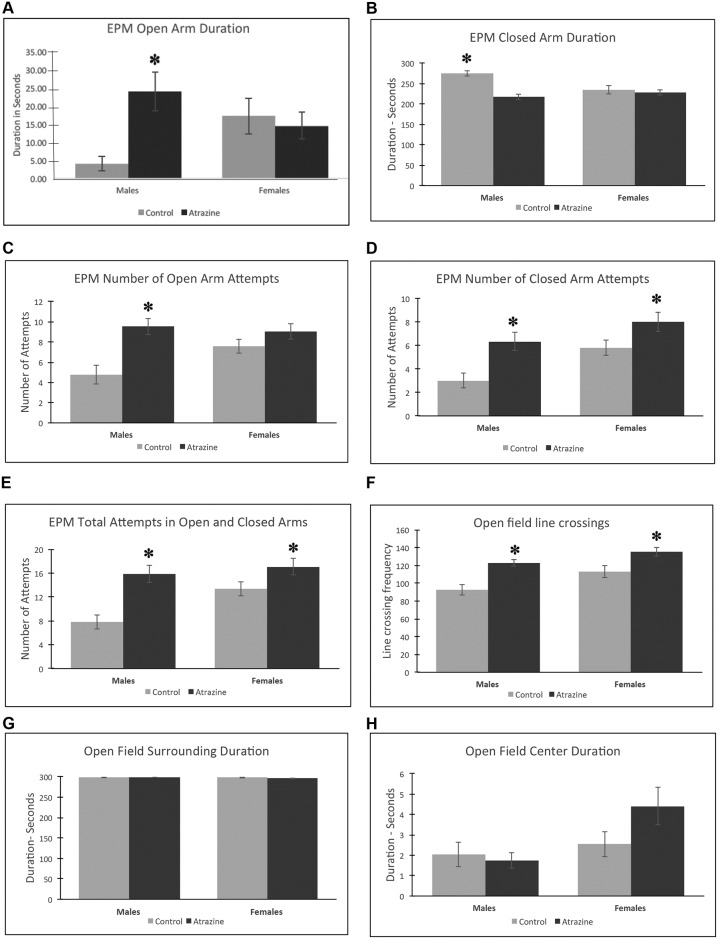
Behavior analysis in F3 generation control and atrazine lineage 10 month old rats. **(A)** elevated plus maze (EPM) open arm duration, **(B)** EPM closed arm duration, **(C)** EPM open arm attempts, **(D)** EPM closed arm attempts, **(E)** EPM total attempts, **(F)** open field line crossings, **(G)** open field surrounding duration, and **(H)** open field center duration. The mean ±SEM is presented with (*) indicating a statistical difference p<0.05 by Students t-test in comparison with control lineage animals.

Previous studies have identified transgenerational impacts of environmental toxicants on behavior [3, 27, 28, 40]. Therefore, the transgenerational F3 generation animals at 11 months of age were analyzed for behavioral alterations using an open field and elevated plus maze to assess anxiety behavior as previously described [40]. The F3 generation atrazine lineage males and females had an increased number of line crossings for the open field test compared to the F3 generation control lineage animals, Fig 2. No changes in other open field test parameters measured were observed. For the elevated plus maze with an open and closed arm, results indicate that the F3 generation atrazine lineage males had significantly increased entry attempts and duration of the time spent in the open arm, Fig 2C & 2E. The F3 generation atrazine lineage females also had increased attempts in the closed arm, Fig 2D, but not in the open arm. Therefore, the F3 generation atrazine lineage males had an altered affective state associated with a reduced anxiety-like behavior and were higher risk takers compared to the control lineage males. Interestingly, the open field and elevated plus maze analysis demonstrated increased locomotor activity for atrazine lineage F3 generation males and females as determined by significant increases in the number of attempts and in line crossings. Therefore, higher levels of general locomotor action independent of affective state was the primary observed behavioral effect in the atrazine lineage animals. In summary, atrazine promoted transgenerational behavioral effects in motor hyperactivity in males and females and a mild anxiolytic effect in males.

**Fig 3:**
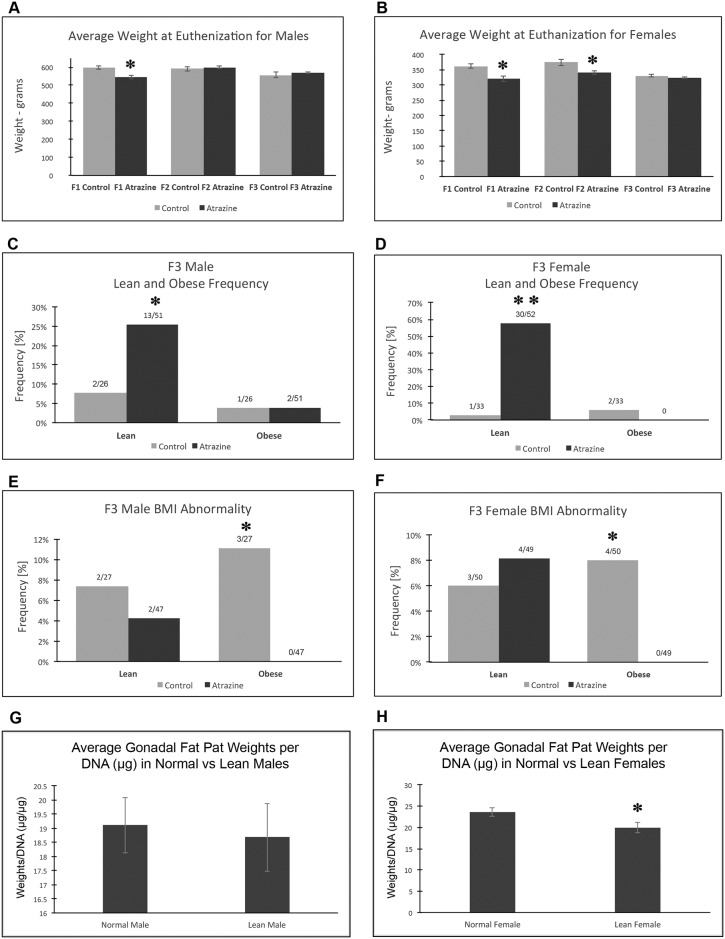
Metabolic disease associated pathology in F1, F2 and F3 generation control and atrazine lineage 1 yr old rats. **(A)** Weight at euthanization for males, **(B)** weight at euthanization for females, **(C)** male lean and obesity frequency, **(D)** female lean and obese frequency, **(E)** male BMI abnormality, **(F)** female BMI abnormality, **(G)** male gonadal fat pad weights per DNA, and **(H)** female gonadal fat pad weights per DNA. The pathology number ratio with total animal number is listed for each bar graph (C-F) or mean ±SEM (A, B, G, H) presented with (*) indicating a statistical difference p<0.05 in comparison with control lineage animals.

**Fig 4:**
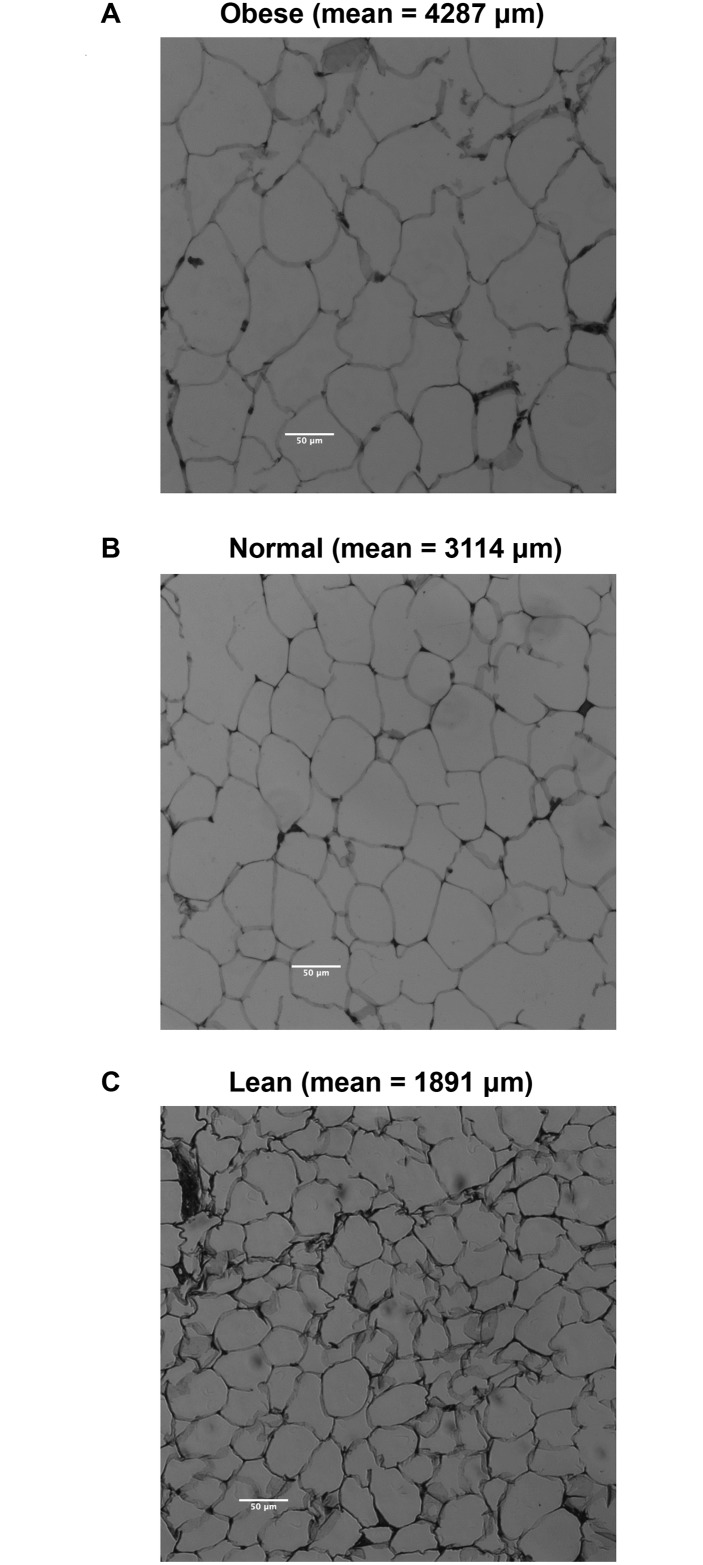
Adipocyte size area and morphology. **(A)** obese, **(B)** normal, and **(C)** lean adipocytes with the mean area listed.

The average weight at euthanization was assessed and the F1 generation atrazine males and the F1 and F2 generation atrazine lineage females had lower mean body weights, Fig 3A & 3B. Due to these lower weights observed, a more extensive analysis of metabolic disease was made in the F3 generation control and atrazine lineages. Several additional parameters of metabolic disease were assessed in the F3 generation control and atrazine lineage males and females. These included body mass index (BMI), gonadal fat pad adipocyte size (cell area), and adiposity as described in the Methods [12]. Previous studies have used these parameters to assess toxicant impacts on transgenerational obesity [8, 11, 12]. Interestingly, the current study has identified the opposite abnormality of a lean (thin) phenotype. The F3 generation control and atrazine lineage males and females were analyzed. The adipocyte size was found to be one of the most reliable parameters to assess metabolic disease [41] and was found to decrease significantly in both F3 generation atrazine lineage male and female adipocytes, Fig 3C & 3D. No increase in adipocyte size was observed or associated with obesity. The range for the adipocyte area in lean females is less than 2671 μm, normal area between 2671 μm– 3912 μm, and in obese is greater than 3912 μm. The range for the adipocyte area in males is less than 2425 μm for lean, normal area between 2425 μm—3912 μm, and obese is greater than 3912 μm. The adipocyte cell areas are shown in Fig 4 for the lean, normal and obese phenotypes. The range for BMI in females is less than 0.6040 g/cm^2^ for lean, between 0.6040–0.7763 g/cm^2^ for normal, and greater than 0.7763 g/cm^2^ for obese. The range for BMI in males is less than 0.7894 g/cm^2^ for lean, between 0.7894 g/cm^2^ and 0.9954 g/cm^2^ for normal and greater than 0.9954 g/cm^2^ for obese. F3 generation atrazine lineage males and females had significantly less obesity associated BMI, Fig 3E & 3F. The BMI change did not correlate well with the lean phenotype due to concomitant changes in muscle mass in the lean individuals [42]. The adiposity assessed with a gonadal fat pad weight per DNA analysis demonstrated a significant decrease in atrazine F3 generation lineage females, but not males, Fig 3G & 3H. Identification of the lean and obese phenotypes in the F3 generation atrazine and control lineages demonstrated a significant increase in the lean phenotype of atrazine lineage males and females, Fig 3C & 3D. Once the lean phenotypes were identified the body weights of the lean versus non-lean F3 generation atrazine lineage animals were compared. In contrast to the lack of weight difference in the comparison of the entire atrazine colony weights in Fig 3A & 3B, the comparison of the lean versus non-lean did have a difference of weight in females (p<10^−4^) and males (p<0.055). The potential that the lean phenotype may be due to a difference in food consumption was investigated with an assessment of daily food consumption in the control versus atrazine lineage F3 generation animals. The mean daily food consumption for F3 generation control lineage 15.76 ± 0.55 grams, and atrazine lineage, 15.0 ± 0.43 grams, animals (n = 10 each group) had no significant difference (p>0.3). Therefore, the lean phenotype does not appear to be due to altered levels of food consumption.

**Fig 5:**
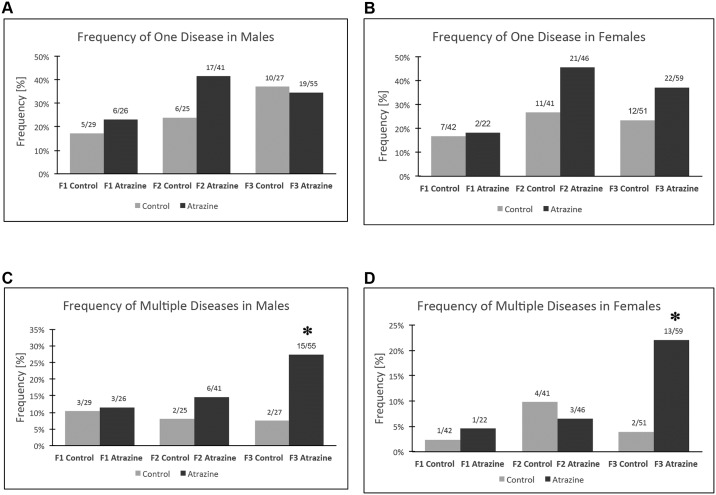
Disease and abnormal pathology frequency. **(A)** frequency of one disease in males, **(B)** frequency of one disease in females, **(C)** frequency of multiple (≥ 2) diseases in males, and **(D)** frequency of multiple (≥ 2) diseases in females. The pathology number ratio with total animal number listed above each bar graph with (*) indicating a statistical difference p<0.05 with Fisher’s exact test in comparison with control lineage animals.

The incidence of disease and abnormalities in all F1, F2 and F3 generation control and atrazine lineage males and females is presented in Fig 5 and in S1 & S2 Tables. The specific disease associated with each individual animal is shown in S1 & S2 Tables for F1 generation males (S1A & S1B Table) and females (S1C & S1D Table), F2 generation males (S1E & S1F Table) and females (S1G & S1H Table), and F3 generation males (S1I & S1J, S2A & S2B Tables) and females (S1K & S1L, S2C & S2D Tables). This information was used for the analysis of single (one disease) and multiple (≥2 diseases) disease, Fig 5. The frequency of single disease in F1, F2 or F3 generation atrazine lineage males or females was not statistically different from control lineage animals, Fig 5A & 5B. The frequency of multiple (≥2 diseases) disease in the F1 and F2 generations was not different between control and atrazine lineages. The frequency of multiple diseases in F3 generation atrazine lineage males and females was significantly increased in comparison to the control lineage, Fig 5C & 5D. Therefore, the F3 generation atrazine lineage males and females had a significant increase in disease, reflected in a susceptibility for multiple diseases. Over 50% of the F3 generation atrazine lineage males (2.7 fold-increase) and/or females (4.6 fold-increase) developed disease and abnormalities.

### Epigenetic transmission of sperm epimutations

The atrazine induced epigenetic transgenerational inheritance of disease and abnormalities requires the germline transmission of epimutations [1, 6, 43]. Previously F3 generation sperm have been shown to have differential DNA methylation regions (DMRs) induced by a variety of environmental toxicants [2, 5, 14]. Interestingly, the sperm epimutations appear to be largely unique to the specific environmental exposure [14]. The current study investigated the sperm epimutations in F1, F2 and F3 generation atrazine and control lineage males. For the F1 and F2 generations (atrazine lineage F1: 3 pools each 8 to 9 individuals, F2: 3 pools each 13 individuals; control lineage F1: 3 pools each 7 to 8 individuals, F2: 3 pools each 5 individuals) males from different litters were pooled into 3 pools with equivalent amounts of sperm DNA from each individual. Sperm DMRs from F3 generation control and atrazine lineage males were individually prepared and analyzed. This was required to identify specific disease-associated biomarkers as discussed below. The epigenetic analysis procedure involved a methylated DNA immunoprecipitation (MeDIP) followed by next generation sequencing (MeDIP-Seq) as described in the Methods [44]. Those DMRs between the control and atrazine lineage sperm samples were identified and termed epimutations. The genome was broken into 100bp windows for DMR identification. The majority of DMRs were single window DMRs, but some had multiple windows and both data sets are shown in Table 1 with different p-value thresholds. A lower p-value was used to reduce background noise and false positives and identified 519 DMRs (p<10^−6^) in the F1 generation sperm, 431 DMRs (p<10^−5^) in the F2 generation sperm, and 958 DMRs (p<10^−9^) in the F3 generation sperm. The different p-values were selected to have similar numbers of DMR to reduce imbalance in subsequent data analysis and comparisons. Although the general approach is to use the same p-value, to allow a more balanced data analysis with comparison between the generations we felt comparable DMR data set size was critical to consider. Additionally, FDR adjusted p-values were also calculated. At an FDR p-value threshold of 0.1, these results identified 5750, 98, and 131231 DMR for the F1, F2 and F3 generations, respectively. The higher number of DMRs and reduced p-value associated with the F3 generation sperm was in part due to the individual animal analyses compared to the pooled analysis in the F1 and F2 generation sperm. The number of independent samples in the F3 generation was 50 for the treatment and 18 for the control. This increased sample size results in an increased statistical power to identify DMRs. To test this observation, the F3 samples were pooled and subsampled in silico, to obtain sample and read numbers similar to the F1 and F2 datasets. This analysis identified 366 DMRs at an edgeR threshold of 1e-05. This confirmed that the increase in DMR numbers in the F3 generation with increased statistical significance was due to the increased sequencing depth because of the individual sample sequencing performed on the F3 generation individuals. Although DMRs at all p-values for the different generations are potentially important, the selected DMR data sets at high p-values are further investigated and presented to demonstrate the phenomenon discussed.

**Table 1:** Generational sperm DMR. DMR numbers for F1, F2, F3 generation sperm. DMRs were defined using an edgeR p-value of 10^−5^, 10^−6^ and 10^−9^ for the F1, F2 and F3 generations, respectively. The DMR edges were extended to include neighboring windows with a p-value less than 0.1. Genomic windows are considered neighboring if they are within 1000 bp of the DMR boundary. In other words, DMR edges are extended until there is no genomic window within 1000 bp with a p-value less than 0.1. The number of DMRs found using different p-value cutoff thresholds is presented. The All Window column shows all DMRs. The Multiple Window column shows the number of DMRs containing at least two significant windows. **(A)** F1 generation sperm DMR numbers at various p value thresholds with p<10^−6^ selected for further analysis. **(B)** F2 generation sperm DMR numbers at various p value thresholds with p<10^−5^ selected for further analysis. **(C)** F3 generation sperm DMR numbers at various p value thresholds with p<10^−9^ selected for further analysis.

**(A) F1 Generation Sperm DMR Numbers and Statistics**						
	**P-value**		**All Window**		**Multiple Window**	
	1e-04		8854		1102	
	1e-05		2067		191	
	**1e-06**		**519**		**69**	
	1e-07		174		35	
	1e-08		64		16	
	1e-09		32		8	
	1e-10		21		3	
	1e-11		16		2	
Number of significant windows	1	2	3	4	5	≥7
Number of DMR (1e-06)	450	53	8	2	1	5
**(B) F2 Generation Sperm DMR Numbers and Statistics**						
	**P-value**		**All Window**		**Multiple Window**	
	1e-04		2522		122	
	**1e-05**		**431**		**29**	
	1e-06		104		7	
	1e-07		36		5	
	1e-08		14		3	
	1e-09		9		2	
	1e-10		4		2	
	1e-11		1		1	
Number of significant windows	1	2	3	4	5	
Number of DMR (1e-05)	402	24	3	1	1	
**(C) F3 Generation Sperm DMR Numbers and Statistics**						
	**P-value**		**All Window**		**Multiple Window**	
	1e-04		53781		17286	
	1e-05		22855		6144	
	1e-06		10190		2219	
	1e-07		4499		866	
	1e-08		2068		357	
	**1e-09**		**958**		**140**	
	1e-10		451		62	
	1e-11		219		30	
Number of significant windows	1	2	3	4	5	≥6
Number of DMR (1e-09)	818	106	21	5	3	5

**Fig 6:**
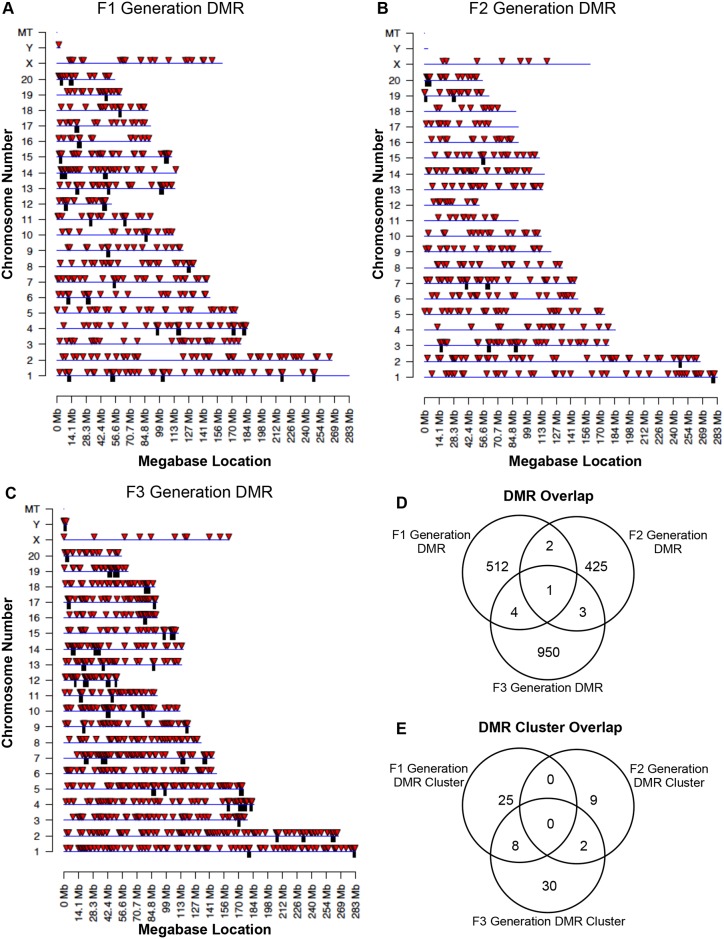
Chromosomal locations of DMR. Red arrowheads indicate DMR and black boxes represent regions with over-representation of DMR (i.e. cluster). **(A)** F1 generation sperm DMR locations on the individual chromosomes. All DMRs at a p-value threshold of 1e-06 are shown. **(B)** F2 generation sperm DMR locations on the individual chromosomes. All DMRs at a p-value threshold of 1e-05 are shown. **(C)** F3 generation sperm DMR locations on the individual chromosomes. All DMRs at a p-value threshold of 1e-09 are shown. **(D)** DMR overlap between the F1, F2 and F3 generation DMR. **(E)** DMR cluster overlap between the F1, F2 and F3 generation DMR clusters.

**Fig 7:**
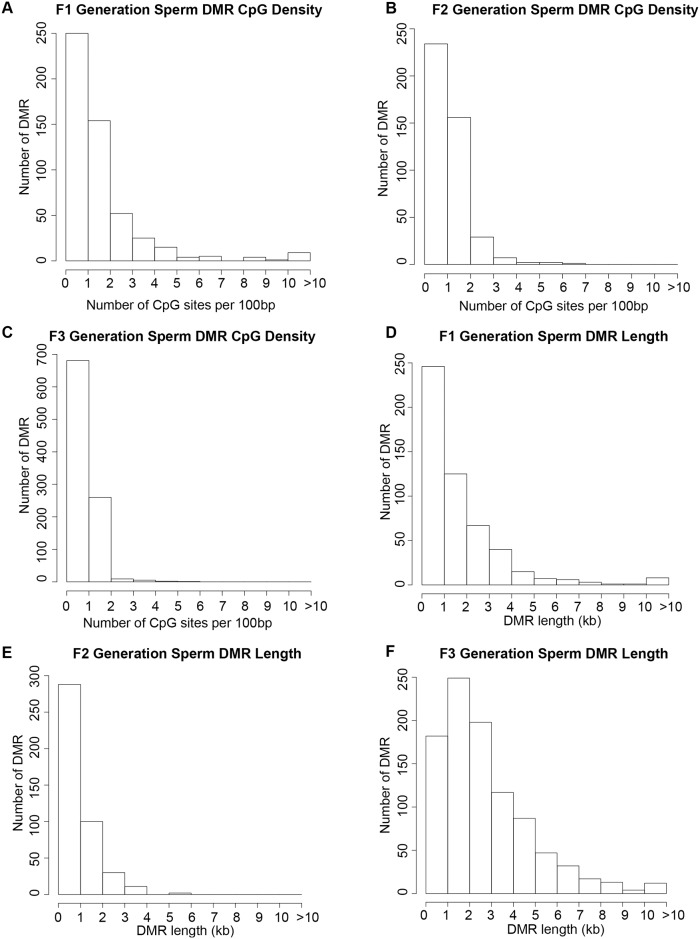
The number of DMRs having specified CpG densities and lengths. **(A)** F1 generation all DMR at a p-value threshold of 1e-06. **(B)** F2 generation all DMR at a p-value threshold of 1e-05. **(C)** F3 generation all DMR at a p-value threshold of 1e-09. **(D)** F1 generation sperm DMR lengths. All DMRs at a p-value threshold of 1e-06. **(E)** F2 generation sperm DMR lengths. All DMRs at a p-value threshold of 1e-05. **(F)** F3 generation sperm DMR lengths. All DMRs at a p-value threshold of 1e-09.

The chromosomal locations of the DMRs for each generation sperm are shown in Fig 6. All chromosomes are involved except the Y in the F2 generation. The red arrowheads identify the DMR sites and the black boxes show chromosomal regions with statistically over-represented clusters of DMR. The biological significance of these DMR clusters appear to be to function as potential epigenetic control regions involving non-coding RNA [7]. The DMR DNA methylation increase or decrease was monitored by analysis of the control portion of the total read depth in the significant windows contained in the DMR. It was found that approximately 25% of DMRs in the F1 generation, approximately 50% of DMRs in the F2 generation, and approximately 5% of DMRs in the F3 generation had an increase in DNA methylation while the rest had a decrease, S3 Fig. One of the primary genetic features of the DMRs is that they exist in CpG deserts within the genome [45]. Atrazine-induced sperm DMRs for the F1 generation (Fig 7A), F2 generation (Fig 7B) and F3 generation (Fig 7C) all demonstrate that the predominant CpG density for the DMRs is 1 CpG / 100 bp. The predominant lengths of the DMRs identified for the atrazine-induced F1 generation sperm (Fig 7D), F2 generation sperm (Fig 7E) and F3 generation sperm (Fig 7F) were 1 kb for F1 generation sperm DMR, 0.5 kb for F2 generation sperm DMR, and 2 kb for F3 generation sperm DMR. Therefore, the genomic features of the DMRs are similar to those previously observed [45].

A comparison of the F1, F2 and F3 generation sperm DMRs was performed to identify those DMRs that overlap. A Venn diagram of the overlap is shown in Fig 6D. The F1 generation actions of atrazine are due to direct exposure of the fetus and germline that will generate the F2 generation, so no transgenerational mechanisms involved. The F3 generation has no direct exposure and is mediated through transgenerational mechanisms. Analysis of potential overlap of the sperm DMRs between generations showed negligible overlap, Fig 6D. The majority of DMRs were unique to each generation. The DMR clusters also had negligible overlap, Fig 6E. The DMR lists for the F1 generation sperm (S3 Table), F2 generation sperm (S4 Table) and F3 generation sperm (S5 Table) present the gene location, size, CpG density, and associated genes for each DMR. The one DMR that overlapped between the generations is DMR15:106498001 and is not annotated.

**Fig 8:**
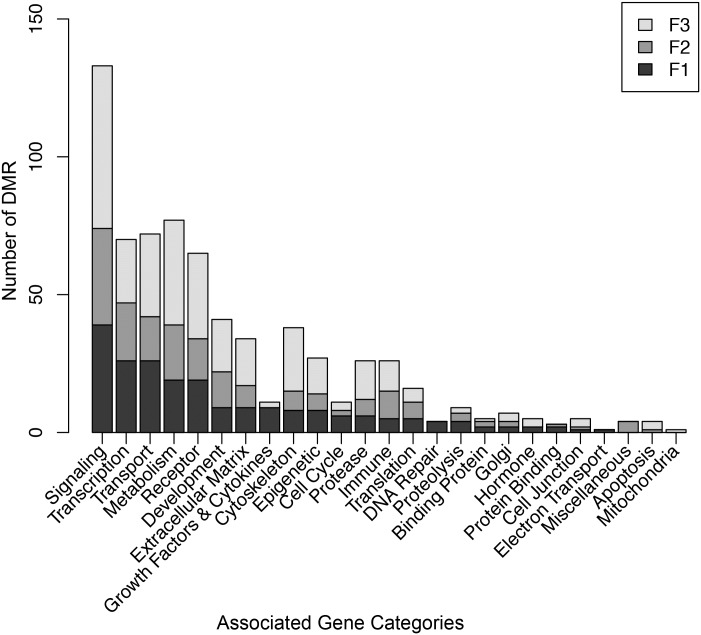
DMR associated gene categories. F1, F2 and F3 generation functional gene categories versus number of DMR per category. Insert color code for F1, F2 and F3 generation presented.

As previously observed [14], the majority of DMRs do not have associated genes but are intergenic or not close to a known gene. The percentage of DMR for each generation with associated genes is approximately 50%, Table 2A. The DMR associated gene categories for each generation identified signaling, transcription, metabolism and transport as the most common gene category for the F1 and F2 generation sperm DMRs, while the F3 generation sperm DMRs associated gene categories were signaling, metabolism, receptor and transport, Fig 8. The associated gene pathway analysis for each generation demonstrated that metabolism, endocytosis and cancer pathways overlap between the F1, F2 and F3 generation sperm DMRs, Table 2B. The cancer pathway and others indicate that generally different sets of genes within any one pathway are involved for each generation, S4 & S5 Figs.

**Table 2:** DMR-associated gene information. **(A)** The percentage of DMR that have associated genes and number. **(B)** KEGG pathways containing DMR-associated genes for each generation. The number of genes falling into that pathway is listed in parentheses. Bolded pathways are in common.

**A** Percentage DMR with gene associations		
F1 Generation 52.8% 274 gene associated DMR		
F2 Generation 50.3% 217 gene associated DMR		
F3 Generation 45.1% 432 gene associated DMR		
**B** Pathways for DMR gene associations		
**F1 Generation**	**F2 Generation**	**F3 Generation**
**Metabolic pathways (13)**	**Metabolic pathways (13)**	**Metabolic pathways (22)**
**Pathways in cancer (12)**	HTLV-I infection (13)	cAMP signaling pathway (9)
MAPK signaling pathway (8)	Cell adhesion molecules (9)	PI3K-Akt signaling pathway (9)
**Endocytosis (7)**	Viral myocarditis (9)	Cell adhesion molecules (9)
Tight junction (6)	Epstein-Barr virus infection (9)	**Endocytosis (9)**
Neuroactive ligand-receptor (6)	Herpes simplex infection (9)	Phagosome (8)
Hypertrophic cardiomyopathy (6)	**Endocytosis (8)**	HTLV-I infection (8)
	Autoimmune thyroid disease (8)	Herpes simplex infection (7)
	Type I diabetes mellitus (8)	Neuroactive ligand-receptor (7)
	Phagosome (8)	Glutamatergic synapse (6)
	Graft-versus-host disease (8)	Tight junction (6)
	Antigen & presentation (8)	Morphine addiction (6)
	Allograft rejection (8)	**Pathways in cancer (6)**
	**Pathways in cancer (6)**	Focal adhesion (6)
	Viral carcinogenesis (6)	
	Taxoplasmosis (6)	
	Influenza A (6)	

**Fig 9:**
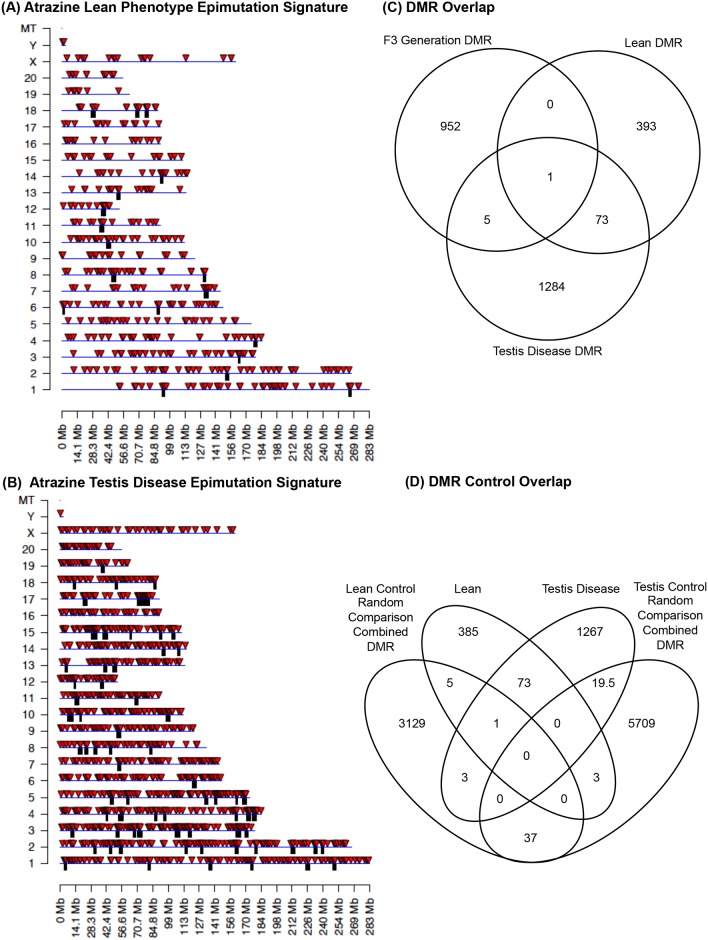
Pathology associated DMRs (epigenetic biomarker signatures). **(A)** Atrazine DMR chromosomal locations for the lean versus non-lean comparisons within the F3 generation atrazine lineage populations. All DMRs at a p-value threshold of 1e-05. **(B)** Atrazine DMR chromosomal location on individual for the testis versus non-testis comparisons within the F3 generation atrazine lineage population. **(C)** DMR overlap between the lean DMRs, testis disease DMRs and original total F3 generation atrazine versus control lineage DMR. **(D)** DMR overlap between the lean and testis disease DMR signatures and the atrazine population random permutation comparisons combined DMR dataset.

The final analysis identified DMRs in the F3 generation individuals with specific disease or abnormalities. Since each individuals sperm was analyzed independently, those animals with disease could be compared with those without disease to identify potential transgenerational epimutation biomarkers for disease. Analysis of the F3 generation atrazine lineage males for non-lean versus lean individuals identified a DMR set that correlated well, Fig 9A. The DMR signature contained 467 DMRs (p<10^−5^) for atrazine lineage non-lean versus lean comparison, Table 3A. The chromosomal locations of the atrazine lean phenotype epimutation signature are presented in Fig 9A showing all chromosomes are involved. This lean epimutation signature provides a potential biomarker DMR signature for the transgenerational lean phenotype. A similar analysis with testis disease in the F3 generation atrazine lineage males was performed. The individuals with testis disease within the atrazine F3 generation lineage were compared to the non-testis disease individuals. A potential epigenetic biomarker DMR signature was identified with 1363 DMR at p<10^−5^, Table 3B. The chromosomal locations of the testis disease epimutation signature are presented in Fig 9B. An overlap analysis demonstrated 74 DMRs overlap between the testis disease DMR signature and the lean phenotype DMR signature, Fig 9C. A list of overlapping lean versus testis disease DMRs is presented in S6 Table. A pathway analysis of the overlapping 74 DMRs did not identify any pathways with three or more associated genes. A comparison of the lean phenotype and testis disease epimutation signatures with the original F3 generation control versus atrazine lineage DMR data set identified negligible overlap and only one DMR overlap with each data set, (DMR 1:195528701 which is not annotated), Fig 9C.

**Table 3:** Disease epigenetic biomarkers. Disease epigenetic (DMR) biomarkers for atrazine lineage F3 generation males. **(A)** Atrazine lean phenotype sperm epimutation signature and statistics with the p<10^−5^ selected for further analysis. **(B)** Atrazine testis disease sperm epimutation signature and statistics with p<10^−5^ selected for further analysis. **(C)** Mean number of DMR identified at a p-value threshold of 10^−5^ for the random permutation replicates. Values for the testis disease and the lean phenotype permutation DMR numbers are presented.

**(A) Atrazine Lean Phenotype (DMR) Epimutation Signature and Statistics**		
**P-value**	**All Window**	**Multiple Window**
1e-04	3152	95
**1e-05**	**467**	**7**
1e-06	56	0
1e-07	10	0
**(B) Atrazine Testis Disease (DMR) Epimutation Signature and Statistics**		
**P-value**	**All Window**	**Multiple Window**
1e-04	7714	430
**1e-05**	**1363**	**35**
1e-06	242	5
1e-07	49	1
1e-08	9	0
1e-09	1	0
**(C) Atrazine Random Comparison Permutation Mean DMR and Statistics**		
**P-value**	**All Window**	**Multiple Window**
Testis Disease Control		
**1e-05**	**288.5**	**10.55**
Lean Phenotype Control		
**1e-05**	**158.7**	**3**

To determine whether the number of DMRs obtained for the lean and testes disease comparisons were significantly increased, a random permutation analysis was performed. The permutation analysis involving the combination of disease and non-disease samples to then randomly select two groups for DMR identification. This provides a random control experiment to determine if the disease versus non-disease identifies a statistically significant increase in DMRs identified. The lean and non-lean individuals were used in 20 different randomly selected permutation data sets of individuals to compare and generate a DMR set as a control comparison, Table 3C. A similar experiment used the testis disease and non-testis disease individuals to randomly select 20 different two group comparisons of animals to generate a control DMR dataset, Fig 3D. A comparison of the combined total random control DMRs with the corresponding lean or testis disease DMR signatures is presented in Fig 9D. For each of the lean and testis disease comparisons, the twenty random permutations generated a null distribution for the number of DMRs. As can be seen in S6 Fig, all of the permutation analyses identified fewer DMRs than the original analysis. Therefore, the lean and testes transgenerational disease comparisons resulted in significantly increased numbers of DMR (p<0.05). No overlap is observed except for the 74 DMRs overlap between the lean versus testis disease DMRs identified, Fig 9D. Therefore, a random permutation analysis did not generate the lean or testis disease DMR signatures observed. These control analyses help validate the transgenerational disease epimutation signatures identified. Observations demonstrate transgenerational biomarker sperm epimutation signatures can potentially be used to identify the lean or testis disease phenotypes.

## Discussion

The current study was designed to investigate the potential transgenerational actions of the herbicide atrazine. Atrazine is the most commonly used herbicide on corn and soy crops, and is extensively used in the central mid-west of the USA [15], S1 Fig. The current study used a mode of administration to control the exposure dose that does not allow this study to be considered for risk assessment. The study was performed to simply determine the potential that atrazine may promote the epigenetic transgenerational inheritance of pathology and sperm epimutations. The results of this study suggest future risk assessments will need to consider multigenerational and transgenerational pathology impacts. Classic toxicology studies involve direct exposure of the individual, while the consideration of impacts on future generations is not currently assessed. Previous studies have shown that direct exposure to atrazine has limited risk [46], however, impacts on subsequent generations have not been previously assessed. A recent study demonstrated that atrazine exposure of a gestating F0 generation rat promoted decreased spermatogenic cell numbers in the F3 generation males [46]. Exposure of a gestating female exposes the F0 exposed female, the F1 generation fetus and the germline generating the F2 generation [1, 3]. Therefore, direct exposure actions can be assessed in the F1 generation, a potential combination of direct exposure and generational exposure in the F2 generation, and transgenerational actions in the F3 generation great-grand offspring and subsequent generations. The molecular actions and mechanisms are very distinct for each of these generations. The direct exposure F1 generation involves somatic cell tissue effects and alteration of classic signaling and developmental pathways to promote any pathologies in those tissues. The endocrine disruptor characteristics of atrazine will have a role in these direct somatic cell actions. The transgenerational actions in the F3 generation involve the germline transmission of epigenetic alterations that change the epigenome of the embryonic stem cells to subsequently alter the differentiation of all cell types generated from that altered stem cell population. The altered germline epigenome will change the stem cell epigenome and transcriptome which impacts all somatic derived epigenomes and transcriptomes [3]. Therefore, the direct somatic cell exposure F1 generation mechanisms contrast significantly with the transgenerational epigenetic altered embryonic stem cell F3 generation mechanisms. The differences observed between the generations appear to in large part be due to the direct exposure versus transgenerational mechanisms.

### Atrazine exposure and pathology analysis

The F0 generation gestating female rats were exposed transiently to vehicle control (DMSO) or atrazine during gonadal sex determination (embryonic days 8–14). The F1 generation animals obtained were bred to generate the F2 generation animals which were then bred to generate the F3 generation [2]. No sibling or cousin breeding was used to avoid any inbreeding artifacts in either the control lineage or atrazine lineage animals [4]. The animals were aged to 1 yr of age to assess pathologies as previously transgenerational pathology has shown to develop between 6–12 months of age. Pathologies previously shown to be altered by the environmentally induced epigenetic transgenerational inheritance were investigated. The litter size and sex ratio (average 5 males and 5 females) were found not to be affected. Weaning weighs were also not affected in any of the generations. No direct toxic effects of atrazine were observed in the F1 generation, except for the reduced weight observed in the 1 yr adults. Pubertal development in the males was altered (early onset puberty) in the F2 generation atrazine lineage males, but not in F1 or F3 generation males. Pubertal development in the females was altered (early onset puberty) in the transgenerational F3 generation atrazine lineage females, but not in F1 or F2 generation females. Early onset puberty in females is a significant problem in the human population today [47, 48]. The male prostate disease incidence in the F1, F2 or F3 generations was not altered. Kidney disease incidence was not altered in either the female or male F1, F2 or F3 generation atrazine lineages. Interestingly the ovary pathologies of reduced primordial follicle pool size and/or presence of polycystic ovaries were also not altered in the F1, F2 or F3 generation females. The most common female transgenerational disease identified in the past with a number of toxicant exposures was ovarian disease [31, 38] and this is the first exposure not shown to influence ovarian disease. In contrast, testis disease involving both increased apoptotic spermatogenic cell numbers and abnormal histology was identified in the F2 and F3 generation atrazine lineage males, S2 Fig. Testis disease was the most predominant transgenerational pathology observed in males, as previously described [2, 14]. A dramatic increase in tumors was observed in the F2 generation males and females, but not F1 or F3 generation animals. The tumors identified in the F2 generation males and females were primarily mammary tumors, along with brain and skin tumors. In summary, no pathology was observed in the F1 generation atrazine lineage males or females compared to control lineage animals. The F2 generation atrazine lineage males had early onset puberty, testis disease and tumors, while the F2 generation atrazine lineage females had increased frequency of tumor development compared to control lineage females. The transgenerational F3 generation atrazine males had testis disease, while the F3 generation females had early onset puberty compared to control lineage.

Behavior analysis of the F3 generation control and atrazine lineage males and females involved open field and elevated plus maze tests. Previous studies have demonstrated increased anxiety in females and decreased anxiety and higher risk taking in males in the F3 generation vinclozolin lineage animals [12]. The F3 generation atrazine lineage males did have a mild anxiolytic effect with lower anxiety and higher risk taking, but females showed no effect compared to controls. However, both the F3 generation atrazine lineage males and females in the elevated plus maze and open field analysis demonstrated higher levels of locomotor activity compared to the control lineage animals. The primary transgenerational behavioral effect observed is increased motor hyperactivity in both males and females compared to the control lineage. Further studies will be needed to investigate the increased motor hyperactivity in atrazine lineage individuals and potential correlations with the lean phenotype.

Potential alterations of a number of different metabolic disease parameters were assessed. Previously, a variety of environmental exposures including DDT, BPA and phthalates, and hydrocarbons [8, 11, 12, 41] have been shown to promote the epigenetic transgenerational inheritance of obesity. Therefore, potential impacts of atrazine on metabolic parameters were investigated. The average weight at 1 yr euthanization was lower in F1 generation atrazine lineage males, but not in F2 or F3 generations. The average euthanization weight for females was also reduced in the F1 and F2 generation atrazine lineages, but not in the F3 generation. A body mass index (BMI) was established for the male and female rats and used to assess BMI associated with obesity and lean phenotype compared to normal for both the control and atrazine lineage F3 generation animals. The BMI analysis identified obese animals in the control lineage males and females, but negligible numbers of obese animals were observed in the atrazine lineage males or females. The BMI was not found to be useful to identify the lean phenotype. The weight analysis of lean versus non-lean F3 generation atrazine lineage animals did identify a reduced weight in the lean animals. Previously, abdominal adiposity was used to assess obesity in the transgenerational DDT lineage rats [12] and a lack of adiposity did help confirm the lean phenotype. Assessment of gonadal fat pad weights also has been used in the past [49], but within group variability has limited its use in assessment of obesity. This is in large part due to the technical difficulties of extracting the entire fat pad. Therefore, we developed an alternate approach to obtain a gonadal fat pad sample tissue weight where it was normalized by the amount of DNA in the sample (μg weight / μg DNA). Adipocytes typically expand in size rather than divide into new cells, consequently the amount of DNA in a standardized volume of adipose tissue is indicative of both the number of cells and cell size in the sample. No change was found in the male epididymal fat pad tissue in the F3 generation atrazine lineage compared to control. However, a significantly reduced weight per DNA for the female gonadal fat pad was observed in the F3 generation atrazine lineage females. Overall the metabolic parameters demonstrate a reduced weight and lean (thin) phenotype and a corresponding reduced obesity rate in the atrazine lineage males and females compared to the control lineage. A previous study demonstrated a lean phenotype induced by the direct actions of atrazine in rats [22].

Previously one of the most consistent metabolic parameters found to correlate with obesity was adipocyte size (area) [50]. Adipocyte size has been shown to be a useful marker for environmental exposures (e.g. tributyltin) that induced epigenetic transgenerational inheritance of obesity [41]. The adipocyte size in gonadal fat pads for both male and female F3 generation atrazine versus control lineages was examined. The lean, normal and obese adipocyte size (area) was used to categorize animals as lean or obese. The male and female F3 generation atrazine lineage animals had a significant increase in the lean phenotype and reduced level of obesity in females. Therefore, the atrazine-induced transgenerational pathology was a lean phenotype in both males and females instead of obesity. Animals that have had a lipectomy, lipotoxicity, or lipodystrophy have exacerbated metabolic disease with low BMI and weight. Adipocyte cell size abnormalities also can indicate a metabolic syndrome even in a non-obese or lean animal [51–53]. A lean phenotype may then be just as significant as an obese phenotype in investigating the etiology of metabolic disease and adipocyte abnormalities.

The total level of pathology per rat was assessed by considering all the different diseases and abnormal pathologies examined (Fig 5). The proportion of animals with only one disease or pathology was not different in the F1, F2 or F3 generation atrazine lineage compared to the control lineage males or females. However, when multiple pathologies and diseases were assessed there was a significant increase in the F3 generation atrazine lineage males and females compared to the control lineage. Atrazine induced the epigenetic transgenerational inheritance of disease susceptibility for multiple pathologies in both males and females. Similar observations were made for other toxicant induced epigenetic transgenerational disease including vinclozolin, DDT and methoxychlor [8, 12, 13]. Pregnant women have been shown to have quantifiable levels of most of the toxicants studied [54]. The possibility that ancestral exposures may increase the disease susceptibility observed today is a novel concept in the etiology of disease and pathology. A recent study in 188 different developed countries indicated that greater than 85% of the human population has one or more chronic diseases at all ages of development [55]. The current study suggests that ancestral exposures to a variety of toxicants and environmental factors will be a critical component to consider in disease etiology.

The atrazine transgenerational phenotype observed in males was predominantly testis disease, a lean phenotype and motor hyperactivity, while in females it was the lean phenotype, early onset puberty and motor hyperactivity. Interestingly, negligible pathology was observed in the F1 generation atrazine lineage compared to the control lineage. Therefore, the direct actions of atrazine on the F1 generation fetus did not promote somatic cell effects to influence later life disease. However, atrazine appears to have altered the epigenetic reprogramming of the primordial germ cells (PGCs) during gonadal sex determination influencing the epigenetic programming of the germ line and promoting subsequent transgenerational disease. A previous study with vinclozolin exposure demonstrated transgenerational impacts on the PGCs epigenome and transcriptome [56]. The major pathology in the F2 generation grand-offspring was tumor development in both males and females, testis disease and pubertal abnormality in males. Therefore, risk assessment of atrazine and most other environmental toxicants only on the directly exposed individual and offspring is likely to result in a significant underestimate of the actual risk. The incorporation of transgenerational risk assessment is going to be essential to consider in the future.

### Atrazine exposure and sperm epimutations

Environmentally induced epigenetic transgenerational inheritance of disease and phenotype variation requires the germline transmission of epigenetic information between generations in the absence of continued exposure [1–3]. This phenomenon has been observed in all organisms investigated so appears highly conserved [3]. Although transgenerational DNA methylation alterations have been well documented [8], other epigenetic processes such as non-coding RNA (ncRNA) and histone modifications have also been shown to have a role in epigenetic transgenerational inheritance [57, 58]. The current study focused on DNA methylation due to its critical role in germline development, in particular in the primordial germ cell and early embryonic epigenetic programming [59, 60]. The potential that atrazine alters the sperm epigenome (DNA methylation) to transmit the transgenerational pathology observed was investigated.

The sperm differential DNA methylation regions (DMRs) in the control versus atrazine lineage F1, F2 and F3 generation males were investigated. The F1 and F2 generation males sperm DNA in each control or atrazine lineage were pooled into 3 different pools of F1 generation atrazine lineage (9 animals each), and control lineage (8 animals each), and F2 generation atrazine lineage (13 animals each) and control lineage (5 animals each for each pool). Therefore, the biological variation (total number of rats) was n = 15–27 for each group while the technical variation (statistical sample size) was n = 3. The DMRs were identified using an established MeDIP-Seq protocol and bioinformatics analysis previously described [44]. The F1 generation DMR dataset with a p<10^−6^ threshold identified 519 DMRs. The F2 generation DMR dataset with a p<10^−5^ threshold identified 431 DMRs. The DMRs at other p-value thresholds are presented and the p-value selected was used to reduce potential background and false positives, and provide similar numbers of DMRs for a comparison between the DMR datasets. In contrast to the F1 and F2 generation analysis, the F3 generation analysis performed an MeDIP-Seq on each individual animal separately. This was done to allow individuals with different pathologies and diseases to be compared to identify potential disease DMR biomarker signatures or sets of DMRs (epimutations). The F3 generation DMR dataset with a p<10^−9^ threshold identified 958 DMRs. The significantly higher number of DMRs observed at all the different p-value thresholds was due to the sequencing depth differences between the F1 and F2 versus the F3 generation analysis. The F1 and F2 generation analysis involved approximately 40 million read pair depth per pool sample with 5–9 individuals. The F3 generation analysis involved approximately 40 million read pairs per individual such that the read depth for a comparable group of individuals was approximately 200–360 million read pairs. Therefore, an imbalance exists in the comparison of the F1 and F2 generation data with the F3 generation data. The p-value threshold used was therefore altered to help adjust this imbalance for comparison.

The chromosomal locations of the F1, F2 and F3 generation control versus atrazine lineage sperm DMR datasets generally involved all chromosomes. Many of the DMRs clustered to produce statistically significant over-represented groups of DMRs. The comparison of the different generation sperm DMRs demonstrated negligible overlap with only one DMR present in all three generations, Fig 6D. In addition, negligible overlap was observed between the DMR clusters identified in the different generations. Since the F1 generation sperm DMRs are due to direct exposure impacts on the germline, it was anticipated that the F3 generation sperm epigenetic alterations would be distinct. This has also been observed in vinclozolin induced epigenetic transgenerational inheritance of sperm epimutations [61]. Therefore, the direct exposure F1 generation impacts on the sperm epigenetics appears to involve a distinct mechanism and promotes in later generations an altered transgenerational epigenetic developmental programming that involves a different molecular mechanism and associated DMRs. In contrast, greater overlap between the F2 generation with the F1 or F3 generation DMR was anticipated. However, negligible overlap was observed with the F2 generation sperm DMR as well. One potential reason might be that the different generation DMRs were in similar regions but not directly overlapping. An analysis was done adding 1 kb or 10 kb flanking regions to the DMR and then assessing overlap, but this did not alter the observations obtained. Therefore, the lack of overlap was not simply due to flanking regions being within 10 kb. Since the F2 generation has direct exposure of the germline during the F1 generation fetal exposure, it appears this mechanism also generates a distinctive set of DMRs in the sperm. Future studies are needed to provide additional technical approaches to validate the MeDIP-Seq data. Currently the bisulfite sequencing technology (e.g. RRBS) is not efficient to accurately assess low density CpG regions in the <10 CpG /100 bp range, but is accurate in higher density >15 CpG / 100 bp regions. Sequence capture technology with bisulfite sequencing may be an approach for future studies. The observations presented suggest the F1, F2 and F3 generation control versus atrazine lineage sperm DMRs are predominantly distinct between each generation.

The genomic features of the atrazine induced F1, F2 and F3 generation sperm DMRs (epimutations) involved low density CpG desert of 1–2 CpG / 100 bp and a size of 1–3 kbase for the DMRs. Therefore, the DMR exist in regions of low density CpG previously termed CpG deserts [45] that appears to involve 5–10 CpG with differential DNA methylation in a few kilobase regions [45]. Previous studies have shown the majority of the genome evolved to have regions with low density CpG due to the high frequency of C to T conversion from CpG methylation [45, 62]. Observations suggest the small clusters of CpG in these regions may be conserved due to their potential regulatory role in epigenetic control regions known to influence areas with clusters of gene expression [63]. The DMRs observed in the F1, F2 and F3 generation control versus atrazine lineage sperm also identified DMR clusters. These DMRs and clusters are proposed to have an important role in the regulation of gene expression as an epigenetic control region [7, 63] involving ncRNA and to correlate with the transgenerational phenotypes observed.

The DMR associated genes were identified as those that were within 10 kb of the DMR. The majority of promoters are approximately 10 kb so this is why a 10 kb region was selected. The DMR associated genes for the F1, F2 and F3 generation control versus atrazine lineage sperm DMR were identified. Generally, 50% of the sperm DMR had associated genes. Gene categories involving signaling, transcription, transport and metabolism were the most predominant gene categories for all three generations. A pathway analysis identified several pathways in common between the generations including metabolism pathways, pathways in cancer and endocytosis. Other pathways were distinct to the generational DMRs, Table 2. Those pathways in common generally had the majority of associated genes within the pathways be distinct between the generational DMR sets as well. Observations suggest the atrazine induced epigenetic transgenerational inheritance of sperm DMRs may have a significant impact on gene expression in subsequent generations [7]. Although many of the DMRs were not associated with genes, they may still have significant impacts on genome activity and gene expression through epigenetic control regions [7, 63]. Similar to imprinting control regions a differential DNA methylation region (DMR) can alter the expression of non-coding RNA (e.g. lncRNA) that can affect gene expression for several megabases in either direction within the chromosome. The atrazine promoted epigenetic transgenerational inheritance of sperm DMRs has the capacity to have significant impacts on gene expression in all somatic cells derived from the embryo, as previously described with other environmental toxicants [38, 64].

### Sperm epimutation biomarkers for transgenerational pathology

The individual male sperm epigenomes were analyzed in the F3 generation atrazine lineage to potentially identify epimutations that may correlate with specific pathologies. The two pathologies in the F3 generation atrazine lineage males examined were testis disease and the lean phenotype. Animals with testis disease were separated as a group and the non-testis disease separated as a group. The non-testis disease group did not contain any animals with a disease or lean phenotype. Comparison of these groups using the MeDIP-Seq data identified a DMR data set or signature that was associated with testis disease. This involved 1363 DMRs at p<10^−5^ as an epimutation signature of sperm from males with testis disease. The chromosomal locations of these DMRs are shown and also involve some clusters of DMRs.

A similar analysis of the F3 generation atrazine lineage males with the lean phenotype was also performed to potentially identify a lean phenotype epimutation signature in sperm. The individuals with the lean phenotype were grouped and compared to those without the lean phenotype within the F3 generation atrazine lineage males. The analysis identified 467 DMRs at p<10^−5^ as an epimutation signature for the male lean phenotype. The chromosomal locations of the DMRs and DMR clusters provide a potential epimutation signature for the lean phenotype.

A comparison of the lean phenotype versus the testis disease epimutation signatures demonstrated 74 DMR overlap, but the majority of the DMRs were distinct between the pathology biomarkers. There was also negligible overlap with the original F3 generation control versus atrazine lineage DMR data sets, presumably due to the fact that those comparisons were very different and involved the control lineage versus the lean phenotype and testis disease epimutation signatures derived within the atrazine lineage males. A more appropriate control comparison is to utilize the males without the specific pathology and make multiple random comparisons of the individuals to generate DMR that can be compared with the pathology epimutation signature. This permutation analysis of the random comparisons of different DMR subsets for either the lean phenotype or testis disease demonstrated negligible (<1.0%) DMR overlap with the pathology epimutation signature. Observations identify unique DMR datasets for the lean phenotype or testis disease as potential epimutation signatures or biomarkers.

This is one of the first demonstrations that a transgenerational disease or pathology specific epigenetic biomarker or diagnostic could be potentially developed and associated with the majority of the animals with the pathology. The observation that the epimutation signatures were distinct between the lean phenotype and testis disease suggests epigenetic diagnostics or biomarkers may be disease specific and unique. The control comparisons suggest a significance for the epimutation signatures identified, which require in the further analysis with increased populations. Therefore, epigenetic biomarkers or diagnostics may provide a more useful molecular diagnostic for disease. Further studies will be required, but the current study suggests the potential to develop epigenetic diagnostics and biomarkers for pathologies and disease is feasible. This could have a significant impact on disease diagnosis and management. In addition, the current identification of the epimutation signature in sperm suggests a preconception diagnostic capability for future disease susceptibility in the offspring.

## Conclusion

The current study demonstrates that atrazine exposure of a gestating female during gonadal sex determination of the fetus promotes the epigenetic transgenerational inheritance of disease and sperm epimutations. Interestingly, no significant pathology was detected in the F1 generation, but a significant increase in disease and pathology was observed in the F3 generation atrazine lineage male and female rats. Therefore, future assessment of exposure toxicity needs to consider transgenerational impacts. A transgenerational increase in testis disease, lean phenotype, behavior motor hyperactivity, and increase in multiple disease susceptibility was observed. Sperm epigenetic alterations were observed in the F1, F2 and F3 generation atrazine lineages and the DMR associated genes identified. The F3 generation males were used to identify unique signatures (groups) of DMRs for the testis disease and lean phenotype. This provides a preliminary proof of concept that epigenetic biomarkers for disease can be identified and potentially used in the future to diagnose disease and disease susceptibility.

## Methods

### Animal studies and breeding

Female and male rats of an outbred strain Hsd:Sprague Dawley^®™^SD^®™^ (Harlan) at about 70 to 100 days of age were fed ad lib with a standard rat diet and ad lib tap water for drinking. To obtain time-pregnant females, the female rats in proestrus were pair-mated with male rats. The sperm-positive (day 0) rats were monitored for diestrus and changes in body weight. On days 8 through 14 of gestation [65], the females were administered daily intraperitoneal injections of Atrazine (25 mg/kg BW/day) or dimethyl sulfoxide (vehicle). The atrazine was obtained from Chem Service, Westchester PA and was injected in approximately 200 microliters of DMSO vehicle as previously described [14]. Treatment lineages are designated ‘control’ and ‘atrazine’ lineages. The gestating female rats treated were designated as the F0 generation. The offspring of the F0 generation rats were the F1 generation. Non-littermate females and males aged 70–90 days from F1 generation control or atrazine lineages were bred to obtain F2 generation offspring. The F2 generation rats were bred to obtain F3 generation offspring. The F1- F3 generation offspring were not themselves treated directly with atrazine. The control and atrazine lineages were housed in the same room and racks with lighting, food and water as previously described [1, 14, 31]. All experimental protocols for the procedures with rats were pre-approved by the Washington State University Animal Care and Use Committee (IACUC approval # 02568–049).

### Tissue harvest and histology processing

Rats at 12 months of age were euthanized by CO_2_ inhalation and cervical dislocation for tissue harvest. Body weights were measured at dissection time. Testis, epididymis, prostate, ovary and kidney were fixed in Bouin’s solution (Sigma) followed by 70% ethanol, then processed for paraffin embedding by standard procedures for histopathological examination. Tissue sections (5 μm) were made and were left either unstained and used for TUNEL analysis or stained with H & E stain and examined for histopathologies.

### Histopathology examination and disease classification

Testis histopathology criteria included the presence of a vacuoles in the seminiferous tubules, azoospermic atretic seminiferous tubules and ‘other’ abnormalities including sloughed spermatogenic cells in center of the tubule and a lack of a tubule lumen. Testis sections were examined by Terminal deoxynucleotidyl transferase-mediated dUTP nick end labeling (TUNEL) assay (In situ cell death detection kit, Fluorescein, Sigma, St. Louis, MO). Prostate histopathology criteria included the presence of vacuoles in the glandular epithelium, atrophic epithelial layer of ducts and hyperplasia of prostatic duct epithelium as previously described [37, 66]. Kidney histopathology criteria included reduced size of glomerulus, thickened Bowman’s capsule and the presence of proteinaceous fluid-filled cysts. A cut-off was established to declare a tissue ‘diseased’ based on the mean number of histopathological abnormalities plus two standard deviations from the mean of control tissues by each of the three individual observers blinded to the treatment groups. This number was used to classify rats into those with and without testis, ovary, prostate or kidney disease in each lineage. A rat tissue section was finally declared ‘diseased’ only when at least two of the three observers marked the same tissue section ‘diseased’.

Ovary sections were stained with hematoxylin and eosin and three stained sections (150 μm apart) through the central portion of the ovary with the largest cross section were evaluated. Ovary sections were assessed for two diseases, primordial follicle loss and polycystic ovary disease. Primordial follicle loss was determined by counting the number of primordial follicles per ovary section and averaging across three sections. An animal was scored as having primordial follicle loss if the primordial follicle number was less than that of the control mean minus two standard deviations. Primordial follicles had an oocyte surrounded by a single layer of either squamous or both squamous and cuboidal granulosa cells [39, 67]. Follicles had to be non-atretic and showing an oocyte nucleus in order to be counted. Polycystic ovaries were determined by microscopically counting the number of small and large cystic structures per section averaged across three sections. A polycystic ovary was defined as having a number of small and / or large cysts that was more than the control mean plus two standard deviations. Cysts were defined as fluid-filled structures of a specified size that were not filled with red blood cells and which were not follicular antra. A single layer of cells may line cysts. Small cysts were 50 to 250 μm in diameter measured from the inner cellular boundary across the longest axis, while large cysts were >250 μm in diameter. Percentages of females with primordial follicle loss or polycystic ovarian disease were computed.

### Obesity and lean phenotype analysis

Obesity and the lean phenotype were assessed with an increase in adipocyte size (area), body weight and abdominal adiposity. The obesity classification has been previously defined as these abnormalities and the presence of associated pathologies [68–72]. Body mass index (BMI) was calculated with weight (g) / length (cm)^2^. Gonadal fat pad slides were imaged using a Nikon Eclipse E800 microscope (10x) with an AVT Prosilica GE1050C Color GigE camera. Five field of view image captures were taken per slide in varying parts of the fat pad. Adipocyte size was measured converting pixels into microns using Adiposoft [73]. Measurements of the 20 largest cells from each image for a total of 100 were averaged as hypertrophic cells are the most metabolically relevant and susceptible to cell death [51]. Obesity and lean phenotypes were determined utilizing the mean of the control population males and females and a cut off of 1.5 standard deviations above and below the mean.

In order to confirm the lean and obese phenotypes, a novel test was developed to determine the weight of the fat pad section per DNA content. Gonadal fat pad adipose tissue frozen in phosphate buffered saline (PBS) was thawed. A small portion of the fat pad was weighed and placed into a container with 250μl of PBS. The tissue was sonicated for 1.5 minutes at 30 second intervals and then centrifuged at 10,000g for 10 minutes at 4°C. The lysate was collected and the DNA content was analyzed using Invitrogen^™^ Qubit^™^ 3.0 Fluorometer Broad Spectrum Double-Stranded DNA assay. The weights of the original sample (μg) were divided by the quantity of DNA (μg) to get the average weight per DNA in the sample.

### Behavior analysis

Behavior analysis was performed with both an elevated plus maze and open field analysis as previously described [74, 75]. F3 generation male and female Sprague-Dawley rats from control and atrazine lineages were used for the behavioral studies at 11 months of age. Elevated Plus-Maze tests were carried out between 9–10 am, and the same rats were always tested the following day at the same time for the Open Field test. Elevated plus maze data were obtained from 27 atrazine lineage males, 28 atrazine lineage females, 17 control males, and 27 control females. Open field data were obtained from 25 atrazine lineage males, 27 atrazine females, 18 control males, and 34 control females.

The elevated plus-maze consisted of a ‘‘plus”-shaped platform made of black opaque Plexiglas, with each platform 10 cm in width and 50 cm in length, creating a 10x10 cm neutral zone in the center. The plus-maze was elevated 50 cm from the floor. Two of the arms were enclosed with black Plexiglas walls 40 cm high, with no ceiling. The elevated plus-maze relies on the animal’s natural fear of open spaces, and the percent time spent on the open arms and percent of open arm entries comprises a general analysis of anxiety [76]. For this task, rats were placed individually into the center (neutral) zone of the maze, facing an open arm. Rats were allowed to explore for a 5 min period, and the number of open and closed arm entries and time spent on the open and closed arms were recorded. Entries were documented when a rat’s snout crossed into an open or closed arm. Animals were considered to be in the open or closed arms only when all four paws crossed out of the neutral zone.

The Open Field test consisted of a transparent Plexiglas 58x58 cm base and four 39.5 cm tall walls. The 58x58 cm base was divided up into a 4x4 grid with 14.7x14.7 cm sized squares made using red tape. The Open Field test has been validated for measuring motor behavior, ambulation and anxiety [77]. Rats were individually placed in the central 2x2 square and allowed to explore for a 5 min period. The duration spent in the central 2x2 area, duration spent in the surrounding (outside of the 2x2) area, and number of line crossings were recorded. Animals were considered to be in the central or surrounding area when all four paws were in those areas. A line crossing was counted when a rat’s snout crossed a tape line. Videos of the behavior were scored by two independent readers that were blinded to the animal identification.

### Statistical analyses for histopathological, obese/lean and behavioral data

For results that yielded continuous data (age at puberty, weight at euthanization, fat sample weights per DNA, behavioral parameters) treatment groups were analyzed using Student’s t-test. For results expressed as the proportion of affected animals that exceeded a pre-determined threshold (testis, prostate, kidney or ovary disease frequency, tumor frequency, lean/obese frequency) groups were analyzed using Fisher’s exact test.

### Overall disease incidence

The incidence of disease in rats from each lineage was assessed and the proportion of individual disease and multiple disease incidences was computed. For the individual diseases, only those rats that showed a presence of one disease are included in the computation. For the multiple diseases, the total number of diseases for each rat was assessed and the number added up for each of the rats. The single or multiple disease proportions are listed in S1 Table.

### Epididymal sperm collection and DNA isolation

The epididymis was dissected free of fat and connective tissue, a small cut made to the cauda and the tissue placed in 6 ml of phosphate buffer saline (PBS) for 20 minutes at 37°C and then kept at 4°C to immobilize the sperm. The epididymal tissue was coarsely minced and the released sperm centrifuged at 10,000 x *g* for 5 min and pellet resuspended in fresh NIM buffer and stored at -20°C until processed further. Fifty to hundred μl of rat sperm suspension were used for DNA extraction, then 820 μL DNA extraction buffer and 80 μl 0.1M DTT added. The sample was incubated at 65°C for 15 minutes. Following this incubation 80 μl proteinase K (20 mg/ml) was added and the sample was incubated at 55°C for 2 hours under constant rotation. Then 300 μl of protein precipitation solution (Promega Genomic DNA Purification Kit, A795A) were added, the sample mixed thoroughly and incubated for 15 min on ice. The sample was centrifuged at 13,500 rpm for 20 minutes at 4°C. One ml of the supernatant was transferred to a 2 ml tube and 2 μl of glycoblue and 1 ml of cold 100% isopropanol were added. The sample was mixed well by inverting the tube several times then left in -20°C freezer for at least one hour. After precipitation the sample was centrifuged at 13,500 rpm for 20 min at 4°C. The supernatant was taken off and discarded without disturbing the (blue) pellet. The pellet was washed with 70% cold ethanol by adding 500μl of 70% ethanol to the pellet and returning the tube to the freezer for 20 minutes. After the incubation the tube was centrifuged for 10 min at 4°C at 13,500 rpm and the supernatant discarded. The tube was spun again briefly to collect residual ethanol at bottom of tube and then as much liquid as possible was removed with gel loading tip. Pellet was air-dried at RT until it looked dry (about 5 minutes). Pellet was then resuspended in 100 μl of nuclease free water. For F1 and F2 generations equal amounts of DNA from each individual’s sperm sample was used to produce three different DNA pools per lineage and the pooled DNA used for methylated DNA immunoprecipitation (MeDIP). For F3 generation each individual’s sperm sample was analyzed separately for MeDIP-Seq.

### Methylated DNA immunoprecipitation MeDIP

Methylated DNA Immunoprecipitation (MeDIP) with genomic DNA was performed as follows: rat sperm DNA pools for the F1 and F2 generation were generated using equal amounts of genomic DNA from each individual to create 3 pools each of control and atrazine lineage animals. For the F3 generation DNA from each rat was processed for MeDIP separately. The genomic DNA was sonicated using the Covaris M220 the following way: up to 6μg of pooled or individual animal sperm genomic DNA was diluted to 130 μl with TE buffer (10mM Tris HCl, pH7.5; 1mM EDTA) into the appropriate Covaris tube. Covaris was set to 300 bp program and the program was run for each tube in the experiment. 10 μl of each sonicated DNA was run on 1.5% agarose gel to verify fragment size. The sonicated DNA was transferred from the Covaris tube to a 1.7 ml microfuge tube and the volume measured. The sonicated DNA was then diluted with TE buffer to 400 μl, heat-denatured for 10min at 95°C, then immediately cooled on ice for 10 min. Then 100μl of 5X IP buffer and 5μg of antibody (monoclonal mouse anti 5-methyl cytidine; Diagenode #C15200006) were added to the denatured sonicated DNA. The DNA-antibody mixture was incubated overnight on a rotator at 4°C.

The following day magnetic beads (Dynabeads M-280 Sheep anti-Mouse IgG; Life Technologies 11201D) were pre-washed as follows: The beads were resuspended in the vial, then the appropriate volume (50 μl per sample) was transferred to a microfuge tube. The same volume of washing buffer (PBS with 0.1% BSA and 2mM EDTA) (at least 1 ml) was added and the bead sample was resuspended. Tube was then placed into a magnetic rack for 1–2 minutes and the supernatant discarded. The tube was removed from the magnetic rack and the washed beads were resuspended in the same volume of 1xIP buffer (50 mM sodium phosphate pH 7.0, 700 mM Nacl and 0.25% triton-X-100) as the initial volume of beads. 50μl of beads were added to the 500μl of DNA-antibody mixture from the overnight incubation, then incubated for 2h on a rotator at 4°C.

After the incubation the beads were washed three times with 1X IP buffer. The tube was placed into magnetic rack for 1–2 minutes and the supernatant discarded, then washed with 1xIP buffer 3 times. The washed beads are then resuspended in 250μl digestion buffer (5mM Tris PH8, 10.mM EDT4, 0.5% SDS) with 3.5μl Proteinase K (20mg/ml). The sample was then incubated for 2–3 hours on a rotator at 55°. 250μl of buffered Phenol-Chloroform-Isoamylalcohol solution were added to the sample and the tube vortexed for 30 sec then centrifuged at 14,000rpm for 5min at room temperature. The aqueous supernatant was carefully removed and transferred to a fresh microfuge tube. Then 250μl chloroform were added to the supernatant from the previous step, vortexed for 30sec and centrifuged at 14,000rpm for 5min at room temperature. The aqueous supernatant was removed and transferred to a fresh microfuge tube. To the supernatant 2μl of glycoblue (20mg/ml), 20μl of 5M NaCl and 500μl ethanol were added and mixed well, then precipitated in -20°C freezer for >1 hour to overnight.

The DNA precipitate was centrifuged at 14,000rpm for 20min at 4°C and the supernatant removed, while not disturbing the pellet. The pellet was washed with 500μl cold 70% ethanol in -20°C freezer for 15 min. then centrifuged again at 14,000rpm for 5min at 4°C and the supernatant discarded. The tube was spun again briefly to collect residual ethanol at bottom of tube and then as much liquid as possible was removed with gel loading tip. Pellet was air-dried at RT until it looked dry (about 5 minutes) then resuspended in 20μl H_2_O or TE. DNA concentration was measured in Qubit (Life Technologies) with ssDNA kit (Molecular Probes Q10212).

### MeDIP-Seq analysis

The MeDIP DNA was used to create libraries for next generation sequencing (NGS) using the NEBNext^®^ Ultra^™^ RNA Library Prep Kit for Illumina^®^ (San Diego, CA) starting at step 1.4 of the manufacturer’s protocol to generate double stranded DNA. After this step the manufacturer’s protocol was followed. Each pool or individual sample received a separate index primer. NGS was performed at WSU Spokane Genomics Core using the Illumina HiSeq 2500 with a PE50 application, with a read size of approximately 50 bp and approximately 35 million reads per pool. Six libraries were run in one lane.

### Statistics and bioinformatics

The basic read quality was verified using summaries produced by the FastQC program. The new data was cleaned and filtered to remove adapters and low quality bases using Trimmomatic [78]. The reads for each MeDIP sample were mapped to the Rnor 6.0 rat genome using Bowtie2 [79] with default parameter options. The mapped read files were then converted to sorted BAM files using SAMtools [80]. To identify DMRs, the reference genome was broken into 100 bp windows. The MEDIPS R package [81] was used to calculate differential coverage between control and exposure sample groups. The edgeR p-value [82] was used to determine the relative difference between the two groups for each genomic window. Windows with an edgeR p-value less than an arbitrarily selected threshold were considered DMRs. The DMR edges were extended until no genomic window with an edgeR p-value less than 0.1 remained within 1000 bp of the DMR. CpG density and other information was then calculated for the DMR based on the reference genome.

DMRs were annotated using the biomaRt R package [83] to access the Ensembl database [84]. The genes that overlapped with DMR were then input into the KEGG pathway search [85, 86] to identify associated pathways. The DMR associated genes were manually then sorted into functional groups by consulting information provided by the DAVID [87], Panther [88], and Uniprot databases incorporated into an internal curated database (www.skinner.wsu.edu under genomic data). All molecular data has been deposited into the public database at NCBI (GEO # GSE98683) and R code computational tools available at GitHub (https://github.com/skinnerlab/MeDIP-seq) and www.skinner.wsu.edu.

## Supporting information

S1 Fig Estimated agricultural use for atrazine, 2014. **(A)** Geographical use in the USA and estimated use presented with insert color code. **(B)** Use by year for various crops as indicated by color code in reference to estimated millions pounds used.
(PDF)

S2 Fig Testis disease histology. **(A)** F3 generation control lineage testis with no disease. **(B)** F3 generation atrazine lineage testis with disease. A hematoxylin and eosin stain testis section is presented and micron size marker presented in each panel.
(PDF)

S3 Fig Alteration in DMR DNA methylation for increase or decrease in DNA methylation in (A) F1 generation, (B) F2 generation, and (C) F3 generation. The control percentage (%) of the total raw read depth for each DMR (significant genomic windows contained in DMR) is presented versus the DMRs ordered by control % mean. The circle is where no DMR are identified with statistical change and then decreased or increased DNA methylation identified.
(PDF)

S4 Fig F1, F2, F3 generation DMR associated genes in pathways in cancer. Circled in blue (F1), red (F2) or black (F3) DMR associated genes.
(PDF)

S5 Fig F1, F2, F3 generation DMR associated genes in endocytosis pathway. Circled in blue (F1), red (F2) or black (F3) DMR associated genes.
(PDF)

S6 Fig Atrazine lineage F3 generation male population random permutation analysis. **(A)** Non-testis disease versus testis disease DMRs identification and comparisons. **(B)** Non-Lean versus Lean DMR identification and comparisons. The number of DMRs for all 20 different permutation analyses. The vertical red line shows the number of DMRs found in the original disease analysis, and the number is significantly greater (p<0.05) than the number of DMRs found in random permutation analyses. All DMRs are defined using an edgeR p-value threshold of 1e-05.
(PDF)

S1 Table Individual animal pathologies. **(A)** F1 generation control lineage males. **(B)** F1 generation atrazine lineage males. **(C)** F1 generation control lineage females. **(D)** F1 generation atrazine lineage females. **(E)** F2 generation control lineage males. **(F)** F2 generation atrazine lineage males. **(G)** F2 generation control lineage females. **(H)** F2 generation atrazine lineage females. **(I)** F3 generation control lineage males. **(J)** F3 generation atrazine lineage females. **(K)** F3 generation control lineage males. **(L)** F3 generation atrazine lineage females. The animal number, rate ID, puberty (late or early), ovary disease, kidney disease, tumor disease, lean phenotype, obesity, and total diseases are presented positive with (+), negative with (-) and not analyzed blank space.
(PDF)

S2 Table Lean and obese characteristics in F3 generation control and atrazine lineage animals. **(A)** F3 generation control lineage males, **(B)** F3 generation atrazine lineage males, **(C)** F3 generation control lineage females, and **(D)** F3 generation atrazine lineage females. The animal ID and abnormal weight, BMI, adipose area (lean or obese) and adiposity are indicated with (+) or not effected (-) or not examined, (blank space).
(PDF)

S3 Table F1 generation DMR list with associated genes. The DMR name, chromosome, start site, length (bp), number # significant windows, minimum p-value, CpG number, CpG % density, associated gene and gene category are presented.
(PDF)

S4 Table F2 generation DMR list with associated genes. The DMR name, chromosome, start site, length (bp), number # significant windows, minimum p-value, CpG number, CpG % density, associated gene and gene category are presented.
(PDF)

S5 Table F3 generation DMR list with associated genes. The DMR name, chromosome, start site, length (bp), number # significant windows, minimum p-value, CpG number, CpG % density, associated gene and gene category are presented.
(PDF)

S6 Table Common overlapping DMR associated genes between the testis disease and lean phenotype DMR biomarkers. The overlapping testis disease DMR name, chromosome, lean and testis disease start site, lean and testis disease length (bp), lean and testis disease minimum p-value, lean and testis disease CpG number and density, and DMR associated gene are presented.
(PDF)
