# Supplementary figures and images for: Atrazine induced epigenetic transgenerational inheritance of disease, lean phenotype and sperm epimutation pathology biomarkers
Source: PLoS One. 2017 Sep 20;12(9):e0184306. doi: 10.1371/journal.pone.0184306 (PMC5606923; doi:10.1371/journal.pone.0184306)

**(A) Estimated Agricultural Use for Atrazine, 2014**

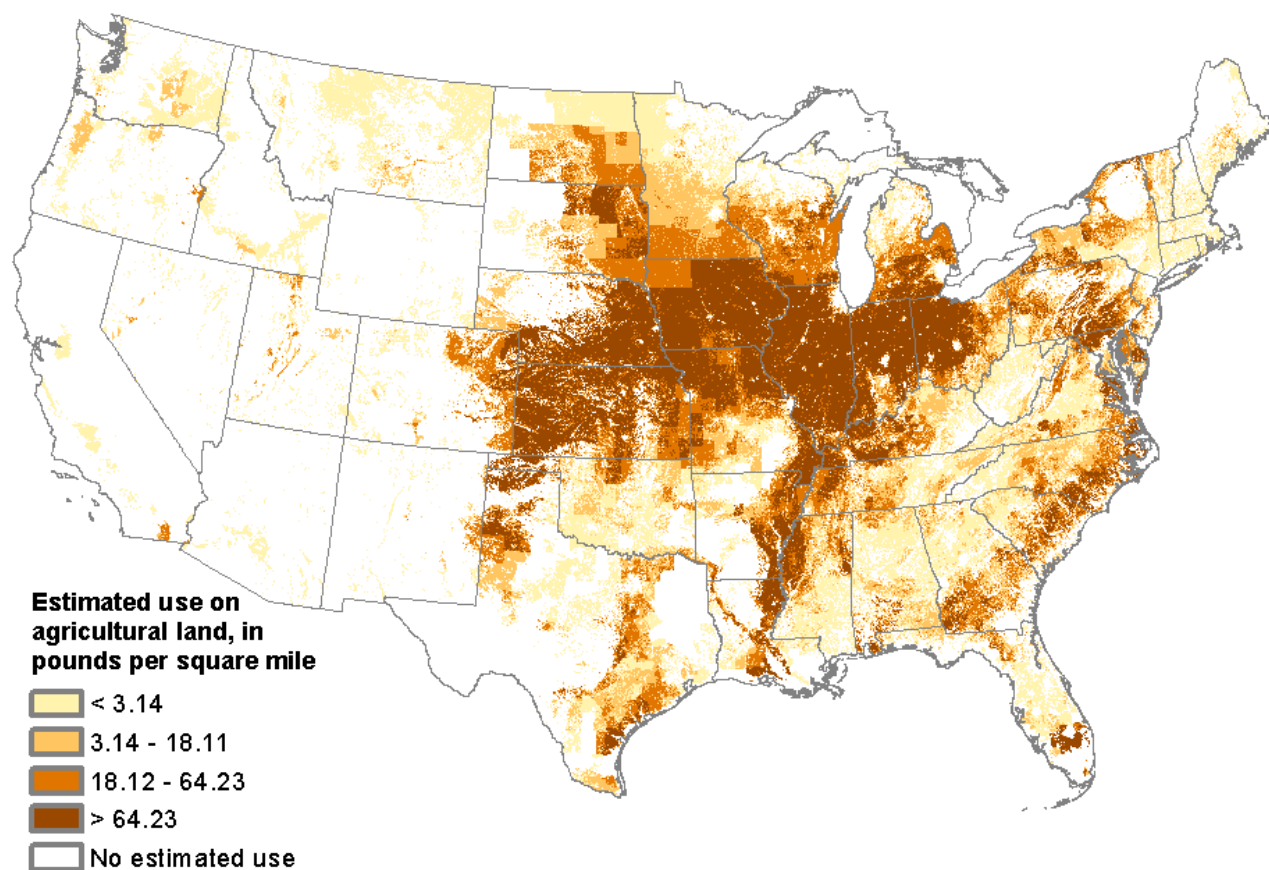

**(B) Use by Year and Crop**

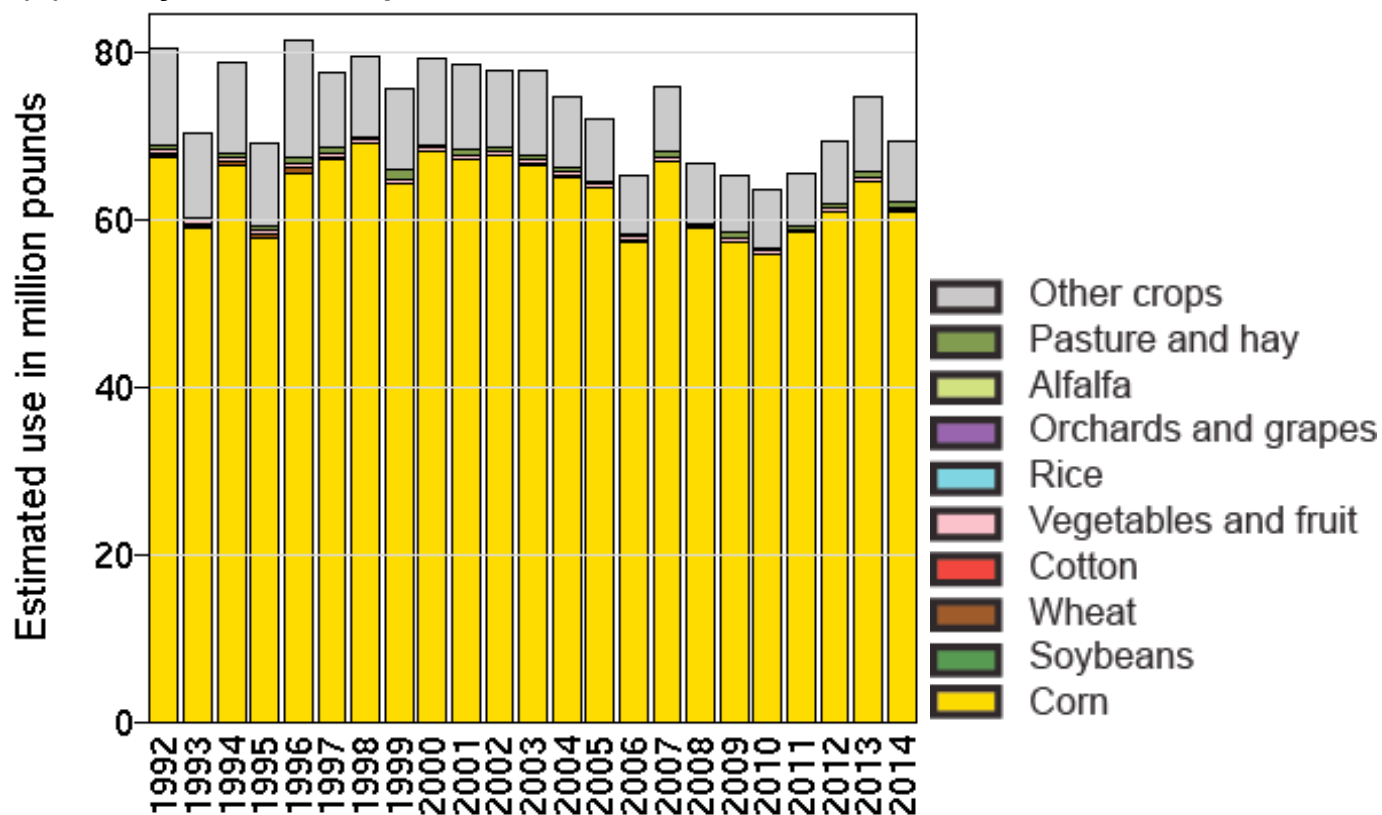

Supplement: S1 Fig — (A) Geographical use in the USA and estimated use presented with insert color code. (B) Use by year for various crops as indicated by color code in reference to estimated millions pounds used. (PDF) [file pone.0184306.s001.pdf]

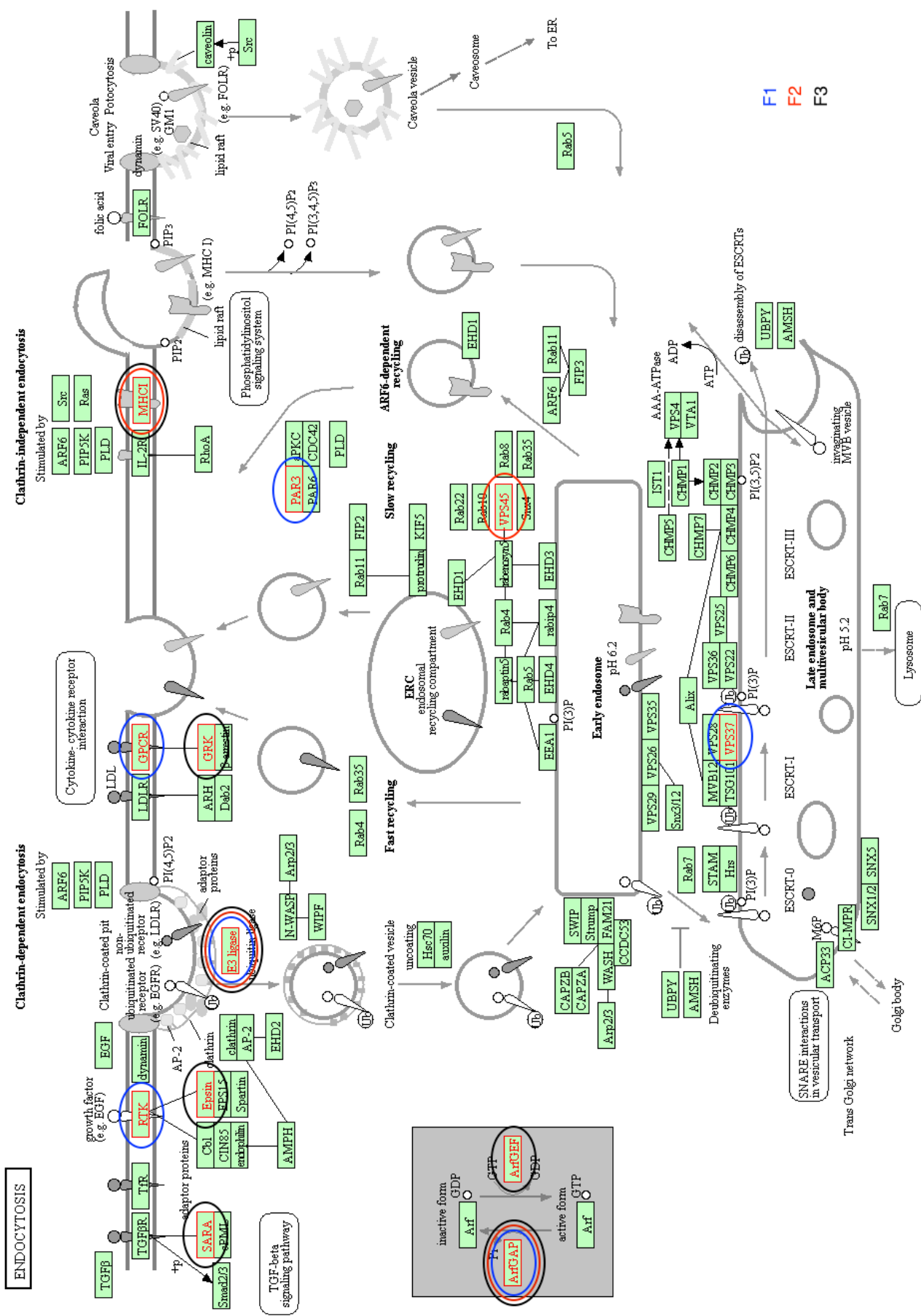

Supplement: S5 Fig — Circled in blue (F1), red (F2) or black (F3) DMR associated genes. (PDF) [file pone.0184306.s005.pdf]
