# Supplementary material for: Atrazine induced epigenetic transgenerational inheritance of disease, lean phenotype and sperm epimutation pathology biomarkers
Source: PLoS One. 2017 Sep 20;12(9):e0184306. doi: 10.1371/journal.pone.0184306 (PMC5606923; doi:10.1371/journal.pone.0184306)
Supplement: S2 Fig — (A) F3 generation control lineage testis with no disease. (B) F3 generation atrazine lineage testis with disease. A hematoxylin and eosin stain testis section is presented and micron size marker presented in each panel. (PDF) [file pone.0184306.s002.pdf]

Supplemental Figure S2

(A) F3 generation control lineage testis histology with normal (non-disease) seminiferous tubules

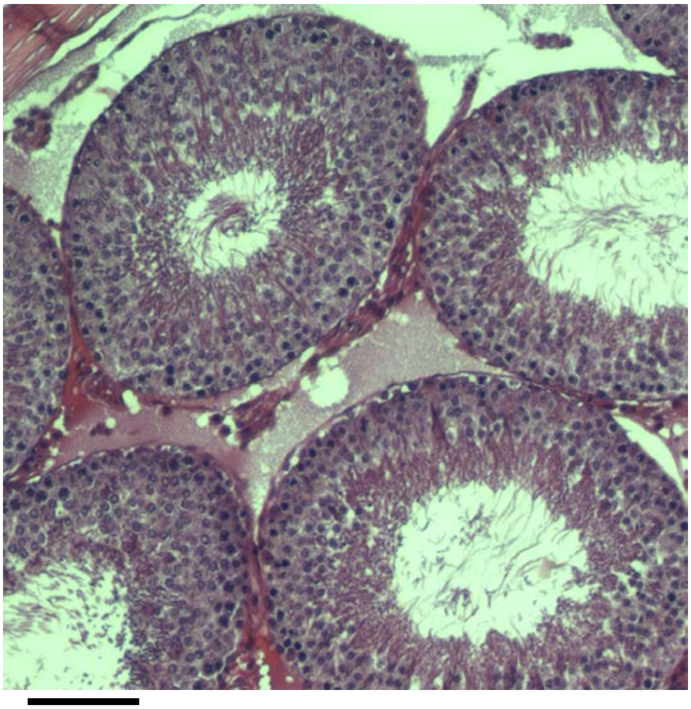

100 micrometers

(B) F3 generation atrazine lineage testis histology with seminiferous tubule atrophy and vacuoles

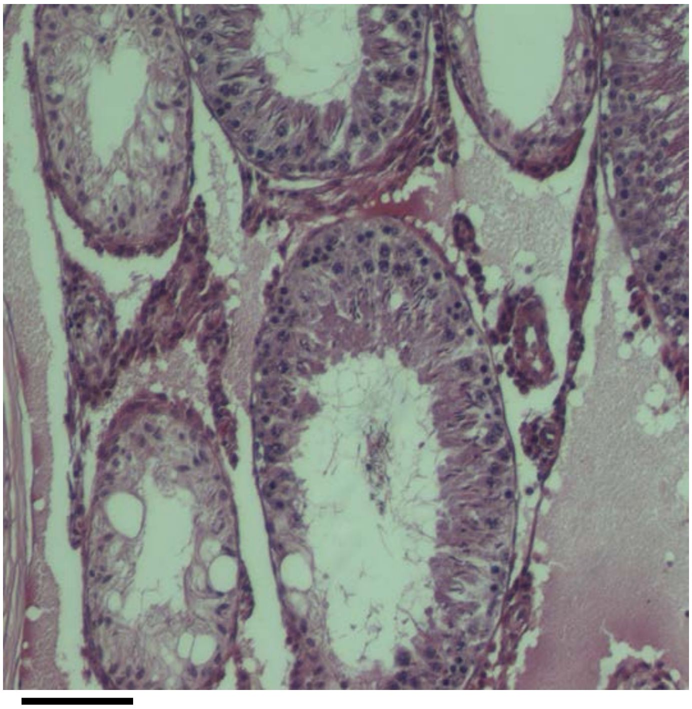

100 micrometers
