# Supplementary material for: Atrazine induced epigenetic transgenerational inheritance of disease, lean phenotype and sperm epimutation pathology biomarkers
Source: PLoS One. 2017 Sep 20;12(9):e0184306. doi: 10.1371/journal.pone.0184306 (PMC5606923; doi:10.1371/journal.pone.0184306)

Supplemental Figure S3

**(A) F1 Generation Sperm DMR Increase or Decrease in DNA Methylation**

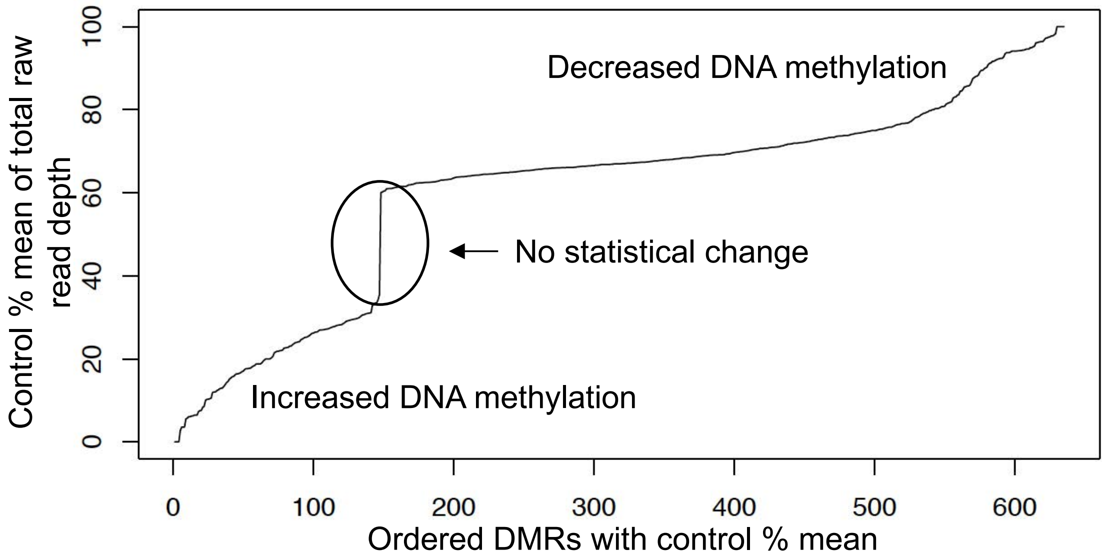


**(B) F2 Generation Sperm DMR Increase or Decrease in DNA Methylation**

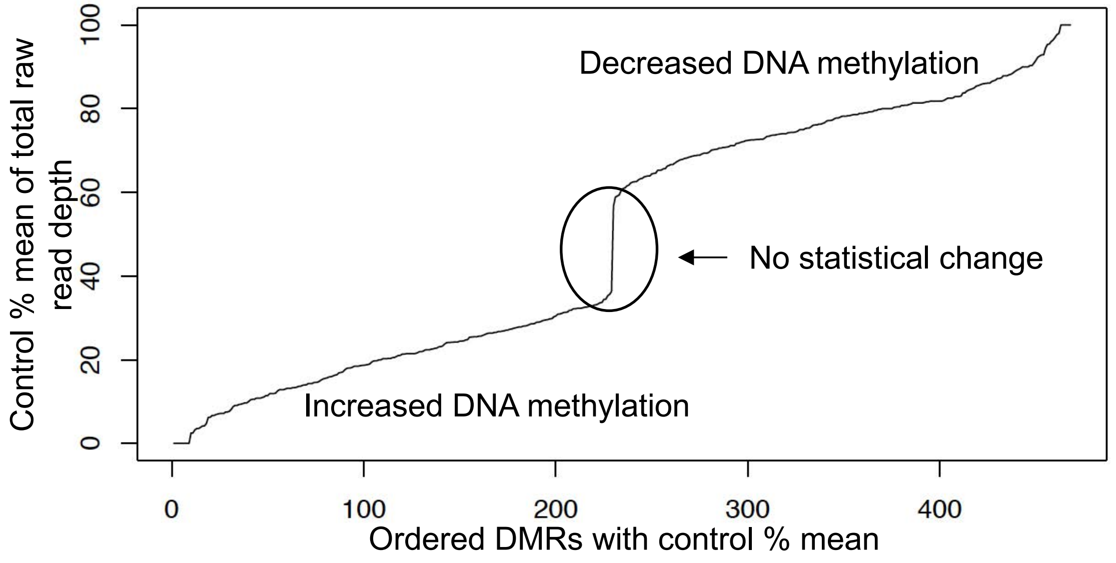


**(C) F3 Generation Sperm DMR Increase or Decrease in DNA Methylation**

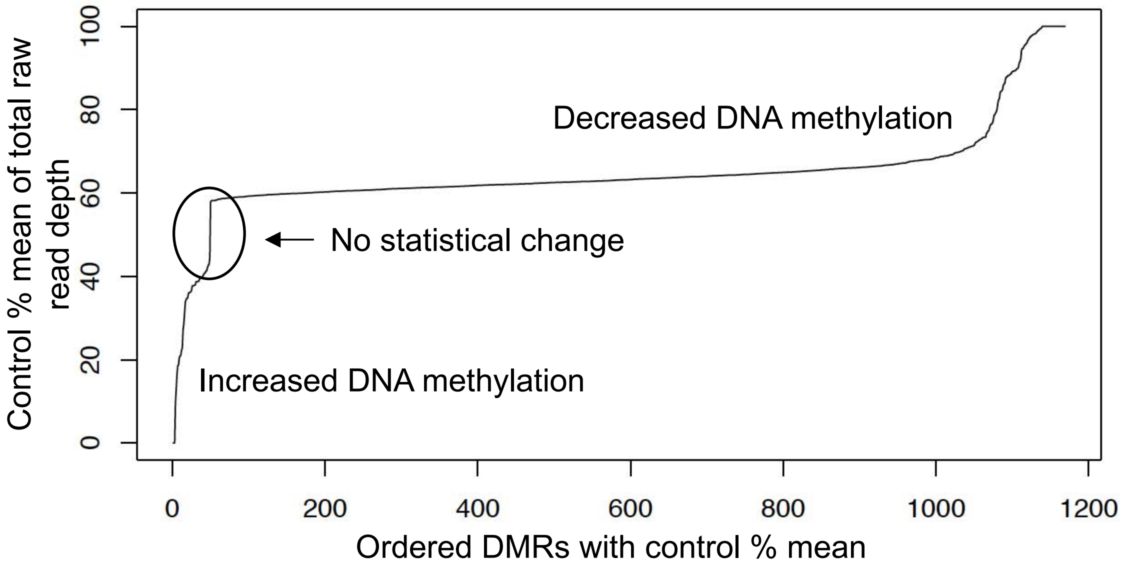

Supplement: S3 Fig — The control percentage (%) of the total raw read depth for each DMR (significant genomic windows contained in DMR) is presented versus the DMRs ordered by control % mean. The circle is where no DMR are identified with statistical change and then decreased or increased DNA methylation identified. (PDF) [file pone.0184306.s003.pdf]
