# Supplementary material for: Atrazine induced epigenetic transgenerational inheritance of disease, lean phenotype and sperm epimutation pathology biomarkers
Source: PLoS One. 2017 Sep 20;12(9):e0184306. doi: 10.1371/journal.pone.0184306 (PMC5606923; doi:10.1371/journal.pone.0184306)

Supplemental Figure S6

(A) Non-Testis Disease DMR versus Testis Disease DMR Comparison

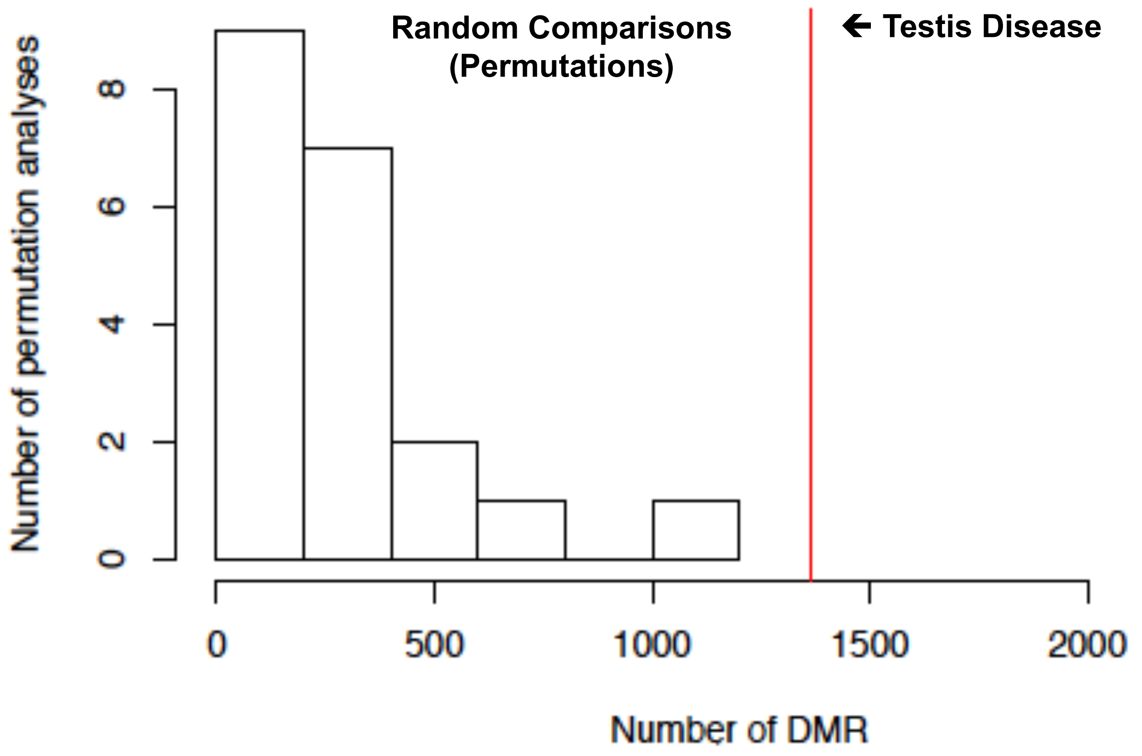

(B) Non-Lean versus Lean Phenotype DMR Comparison

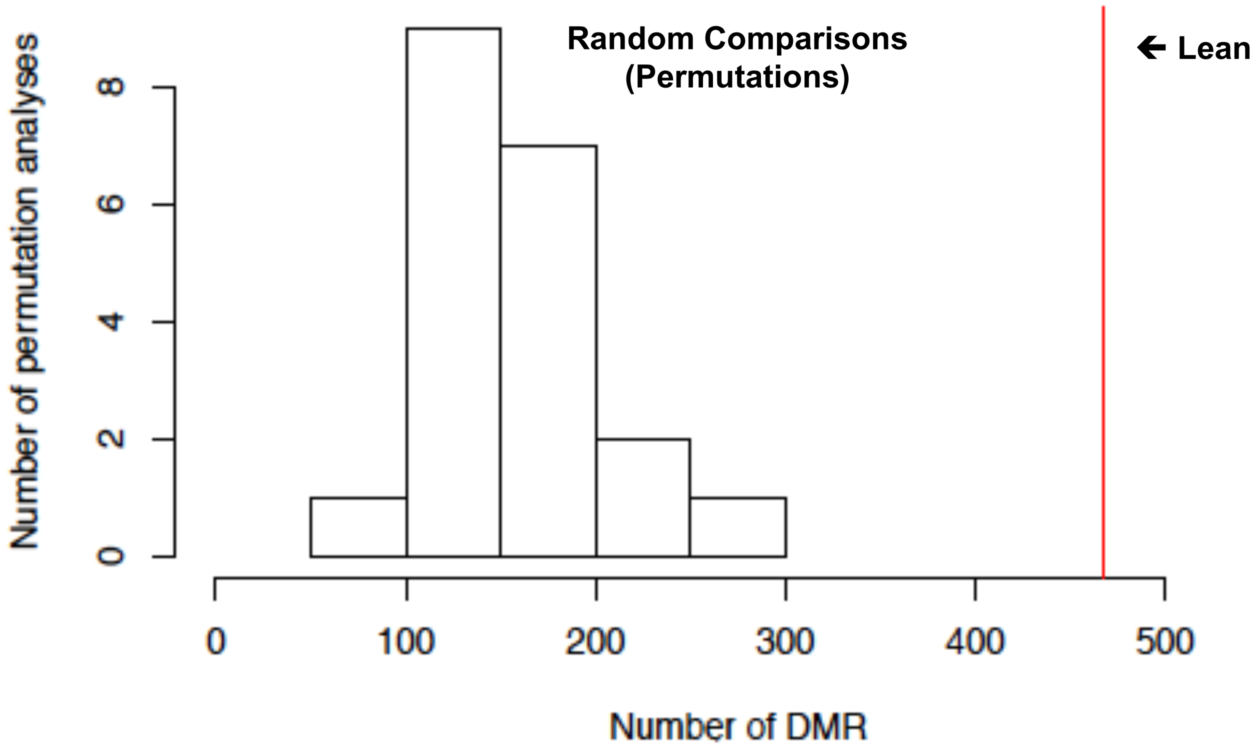

Supplement: S6 Fig — (A) Non-testis disease versus testis disease DMRs identification and comparisons. (B) Non-Lean versus Lean DMR identification and comparisons. The number of DMRs for all 20 different permutation analyses. The vertical red line shows the number of DMRs found in the original disease analysis, and the number is significantly greater (p<0.05) than the number of DMRs found in random permutation analyses. All DMRs are defined using an edgeR p-value threshold of 1e-05. (PDF) [file pone.0184306.s006.pdf]
