# Supplementary material for: Atrazine induced epigenetic transgenerational inheritance of disease, lean phenotype and sperm epimutation pathology biomarkers
Source: PLoS One. 2017 Sep 20;12(9):e0184306. doi: 10.1371/journal.pone.0184306 (PMC5606923; doi:10.1371/journal.pone.0184306)
Supplement: S1 Table — (A) F1 generation control lineage males. (B) F1 generation atrazine lineage males. (C) F1 generation control lineage females. (D) F1 generation atrazine lineage females. (E) F2 generation control lineage males. (F) F2 generation atrazine lineage males. (G) F2 generation control lineage females. (H) F2 generation atrazine lineage females. (I) F3 generation control lineage males. (J) F3 generation atrazine lineage females. (K) F3 generation control lineage males. (L) F3 generation atrazine lineage females. The animal number, rate ID, puberty (late or early), ovary disease, kidney disease, tumor disease, lean phenotype, obesity, and total diseases are presented positive with (+), negative with (-) and not analyzed blank space. (PDF) [file pone.0184306.s007.pdf]

Supplemental Table S1 Individual Animal Pathology

(A) F1 Generation Control Lineage Males

		**Puberty**		**Testes**	**Prostate**	**Kidney**	**Lean**	**Obese**	**Tumor**	**Total**
	Rat ID	**Early**	**Late**							
CM1	AC2-1-2-1	**+**	**-**	**-**	**-**	**+**	**-**	**-**	**-**	**2**
CM2	AC2-1-2-2	**-**	**-**	**-**	**-**	**-**	**-**	**-**	**-**	**0**
CM3	AC2-1-2-3	**-**	**-**	**-**	**-**	**+**	**-**	**-**	**-**	**1**
CM4	AC2-1-2-4	**-**	**-**	**-**		**-**	**-**	**-**	**-**	**0**
CM5	AC2-1-2-5	**-**	**-**	**-**	**-**	**-**	**-**	**-**	**-**	**0**
CM6	AC6-1-6-1	**-**	**-**	**-**	**-**	**-**	**-**	**-**	**-**	**0**
CM7	AC6-1-6-2	**-**	**-**		**+**	**+**	**-**	**-**	**-**	**2**
CM8	AC6-1-6-3	**-**	**-**	**-**	**-**	**-**	**-**	**-**	**-**	**0**
CM9	AC6-1-6-4	**-**	**-**	**+**	**+**	**+**	**-**	**-**	**-**	**3**
CM10	AC4-1-4-1	**-**	**-**				**-**	**-**	**-**	**0**
CM11	AC4-1-4-2	**-**	**-**				**-**	**-**	**-**	**0**
CM12	AC4-1-4-3	**-**	**-**				**-**	**-**	**-**	**0**
CM13	AC4-1-4-4	**-**	**-**				**-**	**-**	**-**	**0**
CM14	AC9-1-4-7	**-**	**-**	**-**	**-**	**-**	**-**	**-**	**-**	**0**
CM15	AC9-1-4-8	**-**	**-**	**-**		**-**	**-**	**-**	**-**	**0**
CM16	AC9-1-4-9	**-**	**-**				**-**	**-**	**-**	**0**
CM17	14C10-1-10-7	**-**	**-**	**-**	**-**	**-**	**-**	**-**	**-**	**0**
CM18	14C10-1-10-8	**-**	**-**	**-**	**-**	**-**	**-**	**+**	**-**	**1**
CM19	14C10-1-10-9	**-**	**-**	**+**	**-**	**-**	**-**	**-**	**-**	**1**
CM20	14C10-1-10-10	**-**	**-**	**-**	**-**	**-**	**-**	**+**	**-**	**1**
CM21	14C12-1-3-5	**-**	**-**				**-**	**+**	**-**	**1**
CM22	14C16-1-6-6	**-**	**-**				**-**	**-**	**-**	**0**
CM23	14C17-1-17-4	**-**	**-**				**-**	**-**	**-**	**0**
CM24	14C17-1-17-5	**-**	**-**				**-**	**-**	**-**	**0**
CM25	14C18-1-18-3	**-**	**-**				**-**	**-**	**-**	**0**
CM26	14C19-1-19-8	**-**	**-**				**-**	**-**	**-**	**0**
CM27	14C19-1-19-9	**-**	**-**				**-**	**-**	**-**	**0**
CM28	14C19-1-19-10	**-**	**-**				**-**	**-**	**-**	**0**
CM29	14C19-1-19-11	**-**	**-**				**-**	**-**	**-**	**0**

(B) F1 Generation Atrazine Lineage Males

		**Puberty**		**Testes**	**Prostate**	**Kidney**	**Lean**	**Obese**	**Tumor**	**Total**
	Rat ID	**Early**	**Late**							
AM1	AA3-1-3-1	**-**	**-**	**-**		**-**	**-**	**-**		
AM2	AA3-1-3-2	**-**	**-**		**-**		**-**	**-**	**-**	
AM3	AA3-1-3-3	**-**	**-**		**-**		**-**	**-**	**-**	
AM4	AA3-1-3-4	**-**	**-**				**-**	**-**	**-**	
AM5	AA4-1-4-1	**-**	**-**	**-**	**-**	**-**	**-**	**-**	**-**	
AM6	AA4-1-4-2	**-**	**-**	**-**		**-**	**-**	**-**	**-**	
AM7	AA4-1-4-3	**-**	**-**			**-**	**-**	**-**	**-**	
AM8	AA4-1-4-4	**-**	**-**	**-**			**-**	**-**	**-**	
AM9	AA4-1-4-5	**-**	**-**				**-**	**-**	**-**	
AM10	AA4-1-4-6	**-**	**-**				**-**	**-**	**-**	
AM11	AA2-1-2-7	**-**	**-**				**-**	**-**	**-**	
AM12	AA2-1-2-8	**-**	**-**				**-**	**-**	**-**	
AM13	AA2-1-2-9	**-**	**-**	**+**	**-**	**-**	**-**	**-**	**-**	**1**
AM14	AA2-1-2-10	**-**	**-**	**+**	**+**	**-**	**-**	**-**	**-**	**2**
AM15	AA8-1-8-5	**-**	**-**	**+**	**+**	**+**	**-**	**-**	**-**	**3**
AM16	AA8-1-8-6	**-**	**-**	**-**	**-**	**+**	**-**	**-**	**-**	**1**
AM17	AA8-1-8-7	**-**	**-**			**-**	**-**	**-**	**-**	
AM18	AA8-1-8-8	**-**	**-**	**+**	**-**	**+**	**-**	**-**	**-**	**2**
AM19	AA9-1-9-3	**-**	**-**	**-**	**-**	**+**	**-**	**-**	**-**	**1**
AM20	AA9-1-9-4	**-**	**-**	**-**	**+**	**-**	**-**	**-**	**-**	**1**
AM21	AA9-1-9-5	**-**	**-**	**-**	**-**	**-**	**-**	**-**	**-**	
AM22	AA9-1-9-6	**-**	**-**	**-**	**-**	**+**	**-**	**-**	**-**	**1**
AM23	AA9-1-9-7	**-**	**-**	**-**	**-**	**+**	**-**	**-**	**-**	**1**
AM24	AA9-1-9-8	**-**	**-**				**-**	**-**	**-**	

AM25	AA9-1-9-9	-	-				-	-	-
AM26	AA9-1-9-10	-	-				-	-	-

(C) F1 Generation Control Lineage Females

		Puberty		Ovary	Kidney	Lean	Obese	Tumor	Total
	Rat ID	Early	Late						
CF1	AC2-1-2-6	-	-	+	-	-	-	-	1
CF2	AC2-1-2-7	-	-	-	-	-	-	-	
CF3	AC2-1-2-8	-	-			-	-	-	
CF4	AC2-1-2-9	-	-			-	-	-	
CF5	AC2-1-2-10	-	-	-	-	-	-	-	
CF6	AC6-1-6-5	-	-	+	-	-	-	+	2
CF7	AC3-1-3-1	-	+			-	-	-	1
CF8	AC3-1-3-2	-	-	-	-	-	-	-	
CF9	AC3-1-3-3	-	-	-	-	-	-	-	
CF10	AC3-1-3-4	-	-	-	-	-	-	-	
CF11	AC3-1-3-5	-	-			-	-	-	
CF12	AC3-1-3-6	-	-	-	+	-	-	-	
CF13	AC9-1-4-1	-	-	-	-	-	-	+	1
CF14	AC9-1-4-2	-	-	-		-	-	-	
CF15	AC9-1-4-3	-	-	-		-	-	-	
CF16	AC9-1-4-4	-	-	-	-	-	-	-	
CF17	14C10-1-10-1	-	-	-	-	-	-	-	
CF18	14C10-1-10-2	-	-	-	-	-	+	-	1
CF19	14C12-1-3-1	-	-		-	-	-	-	
CF20	14C12-1-3-2	-	-	-	+	-	-	-	1
CF21	14C12-1-3-3	-	-	-	-	-	-	-	
CF22	14C14-1-4-1	-	-	+		-	-	-	
CF23	14C14-1-4-2	-	-	-		-	+	-	1
CF24	14C16-1-6-1	-	-	-		-	-	-	
CF25	14C16-1-6-2	-	-	-		-	-	-	
CF26	14C16-1-6-3	-	-	-		-	-	-	
CF27	14C16-1-6-4	-	-	-		-	-	-	
CF28	14C17-1-17-1	-	-	-		-	-	-	
CF29	14C17-1-17-2	-	-	-		-	-	-	
CF30	14C17-1-17-3	-	-	-		-	-	-	
CF31	14C18-1-18-1	-	-	-		-	-	-	
CF32	14C18-1-18-2	-	-	-		-	+	-	1
CF33	14C19-1-19-1	-	-			-	-	-	
CF34	14C19-1-19-2	-	-			-	-	-	
CF35	14C19-1-19-3	-	-			-	-	-	
CF36	14C19-1-19-4	-	-			-	-	-	
CF37	14C19-1-19-5	-	-			-	-	-	
CF38	14C19-1-19-6	-	-			-	-	-	
CF39	14C19-1-19-7	-	-			-	-	-	
CF40	14C20-1-20-1	-	-			-	-	-	
CF41	14C20-1-20-2	-	-			-	-	-	
CF42	14C20-1-20-3	-	-			-	-	-	

(D) F1 Generation Atrazine Lineage Females

		Puberty		Ovary	Kidney	Lean	Obese	Tumor	Total
	Rat ID	Early	Late						
AF1	AA3-1-3-5	-	-			-	-	-	
AF2	AA3-1-3-6	-	-			-	-	-	
AF3	AA3-1-3-7	-	-	-		-	-	-	
AF4	AA3-1-3-8	-	-	-	-	-	-	-	
AF5	AA3-1-3-9	-	-	-	-	-	-	-	
AF6	AA3-1-3-10	-	-	+	-	-	-	-	1
AF7	AA4-1-4-7	-	-			-	-	-	
AF8	AA4-1-4-8	-	-			-	-	-	
AF9	AA4-1-4-9	-	-	-	-	-	-	-	
AF10	AA4-1-4-10	-	-	+		-	-	-	1
AF11	AA2-1-2-1	-	-	-	-	-	-	-	
AF12	AA2-1-2-2	-	-	-		-	-	-	

AF13	AA2-1-2-3	-	-	-	-	-	-	-	
AF14	AA2-1-2-4	-	-	-		-	-	-	
AF15	AA2-1-2-5	-	-	-	-	-	-	-	
AF16	AA2-1-2-6	-	-	-	-	-	-	-	
AF17	AA8-1-8-1	-	-	+	+	-	-	-	**2**
AF18	AA8-1-8-2	-	-	+	-	-	-	-	**1**
AF19	AA8-1-8-3	-	-	+	-	-	-	-	**1**
AF20	AA8-1-8-4	-	-	-	-	-	-	-	
AF21	AA9-1-9-1	-	-	-	-	-	-	-	
AF22	AA9-1-9-2	-	-			-	-	-	

(E) F2 Generation Control Lineage Males

		**Puberty**		**Testes**	**Prostate**	**Kidney**	**Lean**	**Obese**	**Tumor**	**Total**
	Rat ID	**Early**	**Late**							
CM1	AC2-2-1-6	-	-	+	+	-	-	+	-	**3**
CM2	AC2-2-1-7	-	-	+	-	-	-	-	-	**1**
CM3	AC6-2-2-4	-	-	-	-	-	-	-	-	
CM4	AC6-2-2-5	-	-	-	+	-	-	-	-	**1**
CM5	AC6-2-2-6	+	-	-	+	-	-	-	-	**2**
CM6	AC2-2-3-3	-	-	-	-	-	-	-	-	
CM7	AC9-2-6-4	-	-	-	-	-	-	-	-	
CM8	AC9-2-6-5	-	-	-	-	-	-	-	-	
CM9	14C10-2-7-4	-	-	-	-	-	-	-	-	
CM10	14C10-2-7-5	-	-	+	-	-	-	-	-	**1**
CM11	AC2-2-10-5	-	-	-	-	-	-	-	-	
CM12	AC2-2-10-6	-	-	-	-	-	-	-	-	
CM13	AC9-2-11-3	-	-	-	-	-	-	+	-	**1**
CM14	AC6-2-12-6	-	-	-	-	-	-	-	+	**1**
CM15	AC6-2-12-7	-	-	-	-	-	-	-	-	
CM16	AC6-2-12-8	-	-	-	+	-	-	-	-	**1**
CM17	14C17-2-13-6	-	-	-	-		-	-	-	
CM18	14C17-2-13-7	-	-				-	-	-	
CM19	14C17-2-13-8	-	-	-	-		-	-	-	
CM20	14C18-2-16-2	-	-				-	-	-	
CM21	14C18-2-16-3	-	-				-	-	-	
CM22	14C18-2-16-4	-	-				-	-	-	
CM23	14C18-2-16-5	-	-				-	-	-	
CM24	14C18-2-17-4	-	-				-	-	-	
CM25	14C18-2-17-5	-	-				-	-	-	

(F) F2 Generation Atrazine Lineage Males

		**Puberty**		**Testes**	**Prostate**	**Kidney**	**Lean**	**Obese**	**Tumor**	**Total**
	Rat ID	**Early**	**Late**							
AM1	AA3-2-1-6	-	-	+	-	-	-	-	-	**1**
AM2	AA3-2-1-7	-	-			-	-	-	+	**1**
AM3	AA3-2-1-8	-	-	+	-	-	-	-	-	**1**
AM4	AA3-2-1-9	-	-	-	-	-	-	-	-	
AM5	AA3-2-1-10	+	-	+	+	-	-	-	-	**3**
AM6	AA4-2-1-6	-	-	-	-	-	-	-	-	
AM7	AA4-2-1-7	-	-	+	-	-	-	-	-	**1**
AM8	AA4-2-1-8	-	-	-	-	-	-	-	-	
AM9	AA4-2-1-10	-	-	-	-	-	-	-	-	
AM10	AA4-2-2-6	-	-	-	+	-	-	-	-	**1**
AM11	AA4-2-2-7	-	-	-	-	-	-	-	-	
AM12	AA4-2-2-8	-	-	-	-	-	-	-	-	
AM13	AA4-2-2-9	+	-			-	-	-	-	**1**
AM14	AA4-2-2-10	+	-			-	-	-	-	**1**
AM15	AA3-2-2-6	-	-	-	-	-	-	-	-	
AM16	AA3-2-2-7	-	-	+	-	-	-	-	-	**1**
AM17	AA3-2-2-8	-	-	-	-	-	-	-	-	
AM18	AA3-2-2-9	-	-	-	-	-	-	-	-	
AM19	AA3-2-2-10	-	-			-	-	-	+	**1**
AM20	AA2-2-3-4	-	-	+	-	-	-	-	-	**1**
AM21	AA2-2-3-5	-	-	+	+	-	-	-	-	**2**

AM22	AA2-2-3-6	-	-	+	-	-	-	-	-	1
AM23	AA9-2-7-7	-	-	+	-	-	-	-	-	1
AM24	AA9-2-7-8	-	-	+	-	-	-	+	-	2
AM25	AA9-2-7-9	-	-			-	-	-	-	
AM26	AA9-2-7-10	-	-			-	-	+	-	1
AM27	AA2-2-4-6	-	-			-	-	-	-	
AM28	AA2-2-4-7	-	-			-	-	-	-	
AM29	AA2-2-4-8	-	-			-	-	-	-	
AM30	AA2-2-4-9	-	-			-	-	-	-	
AM31	AA8-2-5-4	-	-	+	+	-	-	-	-	2
AM32	AA8-2-5-5	+	-			-	-	-	+	2
AM33	AA8-2-5-6	+	-	+	+	-	-	-	-	1
AM34	AA8-2-5-7	-	-			-	-	-	+	1
AM35	AA8-2-6-5	-	-			-	-	-	-	
AM36	AA9-2-8-5	-	-	-	-	-	-	+	-	1
AM37	AA9-2-8-6	-	-	+	+	-	-	-	-	2
AM38	AA9-2-8-7	-	-	-	-	+	-	-	-	1
AM39	AA9-2-8-8	-	-			-	-	-	-	
AM40	AA9-2-8-9	-	-			-	-	-	-	
AM41	AA9-2-8-10	-	-				-	-	-	

(G) F2 Generation Control Lineage Females

		Puberty		Ovary	Kidney	Lean	Obese	Tumor	Total
	Rat ID	Early	Late						
CF1	AC2-2-1-1	-	-	-	+	-	-	-	1
CF2	AC2-2-1-2	-	-		-	-	-	-	
CF3	AC2-2-1-3	-	+	-	-	-	-	-	1
CF4	AC2-2-1-4	-	-	-	-	-	+	-	1
CF5	AC2-2-1-5	-	+		-	-	+	-	2
CF6	AC6-2-2-1	-	-	-	-	-	-	-	
CF7	AC6-2-2-2	-	-	-	-	-	-	-	
CF8	AC6-2-2-3	-	-	+	-	-	-	-	1
CF9	AC2-2-3-1	-	-	-	-	-	+	-	1
CF10	AC2-2-3-2	-	-	-	-	-	-	-	
CF11	AC2-2-5-1	-	-		-	-	-	-	
CF12	AC9-2-6-1	-	-	+	+	-	-	-	2
CF13	AC9-2-6-2	-	-	-	-	-	-	-	
CF14	AC9-2-6-3	-	-	-	-	-	-	-	
CF15	14C10-2-7-1	-	-	-	-	-	-	-	
CF16	14C10-2-7-2	-	-	-	-	-	-	-	
CF17	14C10-2-7-3	-	-	-	-	-	-	-	
CF18	14C10-2-8-1	-	-		-	-	-	-	
CF19	14C10-2-8-2	-	+	+	-	-	-	-	2
CF20	14C10-2-8-3	-	-	-	-	-	-	-	
CF21	14C10-2-8-4	-	-	-	-	-	-	-	
CF22	AC2-2-10-1	-	-		-	-	+	-	1
CF23	AC2-2-10-2	-	-		-	-	-	-	
CF24	AC2-2-10-3	-	-		-	-	-	-	
CF25	AC2-2-10-4	-	-		-	-	-	-	
CF26	AC9-2-11-1	-	+	+	-	-	-	-	2
CF27	AC9-2-11-2	-	+	-	-	-	-	-	1
CF28	AC6-2-12-1	-	-	-	-	-	-	-	
CF29	AC6-2-12-2	-	-	-	-	-	-	-	
CF30	AC6-2-12-3	-	-	+	-	-	-	-	1
CF31	AC6-2-12-4	-	-			-	-	-	
CF32	AC6-2-12-5	-	-			-	-	+	1
CF33	14C17-2-13-1	-	-			-	-	-	
CF34	14C17-2-13-2	-	-			-	-	-	
CF35	14C17-2-13-3	-	-			-	-	-	
CF36	14C17-2-13-4	-	-			+	-	-	1
CF37	14C17-2-13-5	-	-			+	-	-	1
CF38	14C18-2-16-1	-	-			-	-	-	
CF39	14C18-2-17-1	-	-			-	-	-	
CF40	14C18-2-17-2	-	-			-	-	-	

CF41	14C18-2-17-3	-	-			-	-	-	

(H) F2 Generation Atrazine Lineage Females

		Puberty		Ovary	Kidney	Lean	Obese	Tumor	Total
	Rat ID	Early	Late						
AF1	AA3-2-1-1	-	-	-	+	-	-	-	1
AF2	AA3-2-1-2	-	-	-	+	-	-	-	1
AF3	AA3-2-1-3	-	-	-	-	-	+	+	2
AF4	AA3-2-1-4	-	-			-	-	-	
AF5	AA3-2-1-5	-	-			-	-	-	
AF6	AA4-2-1-1	-	-			-	-	-	
AF7	AA4-2-1-2	-	-	-	-	-	-	-	
AF8	AA4-2-1-3	-	-	+	-	-	-	-	1
AF9	AA4-2-1-4	-	+			-	-	-	1
AF10	AA4-2-1-5	-	-			-	-	+	1
AF11	AA4-2-1-9	-	-			-	-	-	
AF12	AA4-2-2-1	-	+	-	-	-	+	+	3
AF13	AA4-2-2-2	-	-	+	-	-	-	-	1
AF14	AA4-2-2-3	-	-	+	+	-	-	-	2
AF15	AA4-2-2-4	-	+	-	-	-	-	-	1
AF16	AA4-2-2-5	-	+			-	-	-	1
AF17	AA3-2-2-1	-	-	-	-	-	-	-	
AF18	AA3-2-2-2	-	-	-	-	-	-	-	
AF19	AA3-2-2-3	-	-			-	-	-	
AF20	AA3-2-2-4	-	-			-	-	-	
AF21	AA3-2-2-5	-	-	+	-	-	-	-	1
AF22	AA2-2-3-1	-	-	-	-	-	-	-	
AF23	AA2-2-3-2	-	-	-	-	-	-	-	
AF24	AA2-2-3-3	-	-	-	-	-	-	-	1
AF25	AA9-2-7-1	-	-	-	-	-	-	-	
AF26	AA9-2-7-2	-	-	-	-	+	-	-	1
AF27	AA9-2-7-3	-	-	-	-	+	-	-	1
AF28	AA9-2-7-4	-	-			-	-	-	
AF29	AA9-2-7-5	-	-			-	-	+	1
AF30	AA9-2-7-6	-	-			-	-	-	
AF31	AA2-2-4-1	-	-			+	-	-	1
AF32	AA2-2-4-2	-	-			-	-	-	
AF33	AA2-2-4-3	-	-			+	-	-	1
AF34	AA2-2-4-4	-	-			+	-	-	1
AF35	AA2-2-4-5	-	-			-	-	-	
AF36	AA8-2-5-1	-	-	-	-	-	-	-	
AF37	AA8-2-5-2	-	-	-	-	-	+	-	1
AF38	AA8-2-5-3	-	-	-	-	-	-	-	
AF39	AA8-2-6-1	-	-			-	-	-	
AF40	AA8-2-6-2	-	-			-	-	-	
AF41	AA8-2-6-3	-	-			-	+	-	1
AF42	AA8-2-6-4	-	-			-	-	-	
AF43	AA9-2-8-1	-	-			-	-	-	
AF44	AA9-2-8-2	-	-			+	-	-	1
AF45	AA9-2-8-3	-	-			-	+	-	1
AF46	AA9-2-8-4	-	-			-	+	-	1

(I) F3 Generation Control Lineage Males

		Puberty		Testes	Prostate	Kidney	Lean	Obese	Tumor	Total
	Rat ID	Early	Late							
CM1	14C10-3-2-10	-	-	-	-	-	-	+	-	1
CM2	14C10-3-2-8	-	-	-	-	-	-	-	-	
CM3	14C10-3-2-9	-	-	-	-	-	-	-	-	
CM4	14C10-3-3-10	-	-		-	-	-	-	-	
CM5	14C10-3-3-8	-	-	-	-	-	-	-	-	
CM6	14C10-3-3-9	-	-		-	+	+	-	-	2
CM7	14C17-3-4-7	-	-	-	-	-	+	-	-	1
CM8	14C17-3-4-8	-	-	-	-	-	-	-	-	

CM9	14C17-3-4-9	-	-	-	-	-	+	-	-	1
CM10	14C17-3-5-5	-	-	-	-	-	+	-	-	1
CM11	14C17-3-5-6	-	-	-	-	-		-	-	
CM12	14C17-3-5-7	-	-	+	+	-	-	-	-	2
CM13	14C17-3-5-9	-	-	-	-	-	-	-	-	
CM14	14C17-3-6-10	-	-	-	-	-	-	-	-	
CM16	AC2-3-3-9	-	-	-	-	-	-	-	-	
CM17	AC6-3-1-4	-	+	-	-	-	-	+	-	1
CM18	AC6-3-1-5	-	+	-	-	-	-	-	-	1
CM19	AC6-3-1-6	-	-	-	-	-	-	+	-	1
CM20	AC6-3-6-7	-	-	-	-	-	-	-	-	
CM21	AC6-3-6-8	-	-	-	-	-	-	-	-	
CM22	AC6-3-6-9	-	-	-	-	+	-	-	-	1
CM23	AC9-3-4-10	-	-	-	-	-	-	-	-	
CM24	AC9-3-4-8	-	-	-	-	-	-	-	-	
CM25	AC9-3-4-9	-	-	-	-	-	-	-	-	
CM26	AC9-3-5-8	-	-	-	-	-	-	+	-	1
CM27	AC9-3-5-9	-	-	-	-	-	-		-	1

(J) F3 Generation AtrazineLineage Males

		Puberty		Testes	Prostate	Kidney	Lean	Obese	Tumor	Total
		Early	Late							
	Rat ID									
AM1	AA2-3-11-5	-	-	-	-	+			-	1
AM2	AA2-3-11-6	-	-	-	+	+			-	2
AM3	AA2-3-11-7	-	-	-	-	-			-	
AM4	AA2-3-11-8	-	-	-	-	+	+		-	2
AM5	AA2-3-11-9	-	-	-	-	+			-	1
AM6	AA2-3-6-8	-	-	+	-	-	+		-	2
AM7	AA2-3-6-9	-	-	-	-	-			-	
AM8	AA2-3-9-10	-	-	-	-	-			-	
AM9	AA2-3-9-11	-	-	-	-	-			-	
AM10	AA2-3-9-6	-	-	+	-	-			-	1
AM11	AA2-3-9-7	-	-	-	-	-	+		-	1
AM12	AA2-3-9-8	-	-	-	-	-			-	
AM13	AA2-3-9-9	-	-	-	-	-			-	
AM14	AA3-3-1-5	-	-	+	-	-	+		-	2
AM15	AA3-3-1-6	-	-	-	-	-			-	
AM16	AA3-3-10-2	-	-	-	-	+			-	1
AM17	AA3-3-10-3	-	-	-	-	-			-	
AM18	AA3-3-10-4	-	-	-	-	-			-	
AM19	AA3-3-10-5	-	-	+	-	-		+	-	2
AM20	AA3-3-10-6	-	-	-	+	-		+	-	2
AM21	AA3-3-2-8	-	-	+	+	-	+		-	3
AM22	AA3-3-2-9	-	-	-	-	-			-	
AM23	AA3-3-3-2	-	-	-	-	-	+		-	1
AM24	AA3-3-8-10	-	+	+	-	-			-	2
AM25	AA3-3-8-11	-	+	-	-	-			-	1
AM26	AA3-3-8-12	-	+	-	-	-			-	1
AM27	AA3-3-8-13	-	+	+	-	-	+		-	3
AM28	AA3-3-8-14	-	+	-	-	-			-	1
AM29	AA3-3-8-9	-	+	-	-	-			-	1
AM30	AA4-3-4-3	-	-	-	-	-		+	+	2
AM31	AA4-3-4-4	-	-	-	-	-			-	
AM32	AA4-3-4-5	-	-	-	-	-			-	
AM33	AA4-3-4-6	-	-	-	-	-			-	
AM34	AA4-3-4-7	-	-	-	-	-			-	
AM35	AA4-3-4-8	-	-	-	-	-			-	
AM36	AA8-3-2-10	-	-	+	-	-			-	1
AM37	AA8-3-2-11	-	-	+	-	-	+		-	2
AM38	AA8-3-2-7	-	-	-	-	-			-	
AM39	AA8-3-2-8	-	-	+	-	-			-	1
AM40	AA8-3-2-9	-	-	+	-	-			-	1
AM41	AA9-3-12-10	-	-	-	-	-			-	

AM42	AA9-3-12-6	-	-	-	-	-	+		-	**1**
AM43	AA9-3-12-7	-	-	-	-	-			-	
AM44	AA9-3-12-8	-	-	-	-	-		+	-	**1**
AM45	AA9-3-12-9	-	-	-	-	-			-	
AM46	AA9-3-5-10	-	+	+	-	+	+		-	**4**
AM47	AA9-3-5-7	-	+	-	+	-	+		-	**3**
AM48	AA9-3-5-8	-	+	-	-	-			-	**1**
AM49	AA9-3-5-9	-	+	-	-	-			-	**1**
AM50	AA9-3-7-4	-	-	-	-	-	+		-	**1**
AM51	AA9-3-7-5	-	-	-	-	-			-	
AM52	AA9-3-7-6	-	-	-	-	-	+		-	**1**
AM53	AA9-3-7-7	-	-	-	-	+	+		-	**2**
AM54	AA9-3-7-8	-	-	-	-	+	+		-	**2**
AM55	AA9-3-7-9	-	-	-	-	-			-	

(K) F3 Generation Control Lineage Females

	Rat ID	**Puberty Early**	**Puberty Late**	**Ovary**	**Kidney**	**Tumor**	**Lean**	**Obese**	**Total**
CF1	AC2-3-3-1	-	-	-	-	-			
CF2	AC2-3-3-2	-	-	+	-	-			**1**
CF3	AC2-3-3-3	-	-	-	-	-			
CF4	AC2-3-3-4	-	-	-	-	-			
CF5	AC2-3-3-5	-	-		-	+		+	**2**
CF6	AC2-3-3-6	-	-	-	-	-			
CF7	AC2-3-7-1	-	-	-	-	-			
CF8	AC2-3-7-2	-	-	-	-	-			
CF9	AC6-3-1-1	-	+	-	-				**1**
CF10	AC6-3-1-7	-	-	-	-	-			
CF11	AC6-3-1-8	-	+	+	-	-			**2**
CF12	AC6-3-6-1	-	-	-	-	-			
CF13	AC6-3-6-2	-	-	-	-	-			
CF14	AC6-3-6-3	-	-	-	-	-			
CF15	AC6-3-6-4	-	-	-	-	-			
CF16	AC6-3-6-5	-	-	-	-	-			
CF17	AC9-3-4-1	-	-	-	-	-			
CF18	AC9-3-4-2	-	-	-	-	-			
CF19	AC9-3-4-3	-	-	-	-	-			
CF20	AC9-3-4-4	-	-		-	-	+		**1**
CF21	AC9-3-4-5	-	-	-	-	-		+	**1**
CF22	AC9-3-5-1	-	-	-	-	-			
CF23	AC9-3-5-2	-	-	-	-	-			
CF24	AC9-3-5-3	-	-	-	-	-			
CF25	AC9-3-5-4	-	-	-	-	-			
CF26	14C10-3-2-1	-	-	-	+	-			
CF27	14C10-3-2-2	-	-	-	-	-		+	**1**
CF28	14C10-3-2-3	-	-	-	-	-			
CF29	14C10-3-2-4	-	-	+	-	-			**1**
CF30	14C10-3-3-1	-	-	-	-	-			
CF31	14C10-3-3-2	-	-	-	-	-			
CF32	14C10-3-3-3	-	-	-	-	-			
CF33	14C10-3-3-4	-	-			-			
CF34	14C10-3-3-5	-	-		-	-	+		**1**
CF35	14C17-3-4-1								
CF36	14C17-3-4-2								
CF37	14C17-3-4-3						+		**1**
CF38	14C17-3-4-4								
CF39	14C17-3-4-5								
CF40	14C17-3-4-6						+		**1**
CF41	14C17-3-5-1								
CF42	14C17-3-5-2								
CF43	14C17-3-5-3								
CF44	14C17-3-5-4								
CF45	14C17-3-6-1								

CF46	14C17-3-6-2								
CF47	14C17-3-6-3								
CF48	14C17-3-6-4								
CF49	14C17-3-6-5							**+**	**1**
CF50	14C17-3-6-6							**+**	**1**
CF51	14C17-3-6-7							**+**	**1**

(L) F3 Generation Atrazine Lineage Females

		**Puberty**		**Ovary**	**Kidney**	**Tumor**	**Lean**	**Obese**	**Total**
	Rat ID	**Early**	**Late**						
AF1	AA2-3-11-1	-	-	-	-	-			
AF2	AA2-3-11-2	-	-	-	-	-			
AF3	AA2-3-11-3	-	-	-	-	-	+		**1**
AF4	AA2-3-11-4	-	-			-			
AF5	AA2-3-6-1	-	-	-	-	-	+		**1**
AF6	AA2-3-6-2	-	-	-	-	-	+		**1**
AF7	AA2-3-6-3	-	-	-	-	-	+		**1**
AF8	AA2-3-6-4	-	-			-	+		**1**
AF9	AA2-3-6-5	-	-			+	+		**2**
AF10	AA2-3-6-6	-	-			-			
AF11	AA2-3-6-7	-	-			-			
AF12	AA2-3-9-1	-	-	-	-	-			
AF13	AA2-3-9-2	-	-	-	-	-	+		**1**
AF14	AA2-3-9-3	-	-	-	-	-			
AF15	AA2-3-9-4	-	-			-			
AF16	AA2-3-9-5	-	-			-			
AF17	AA3-3-1-1	-	-	-	-	-			
AF18	AA3-3-1-2	-	-	-	-	-	+		**1**
AF19	AA3-3-1-3	-	-			-			
AF20	AA3-3-1-4	-	-			-			
AF21	AA3-3-10-1	-	-	+	-	-	+		**2**
AF22	AA3-3-2-1	-	-	-	-	-	+		**1**
AF23	AA3-3-2-2	-	-	+	-	-	+		**2**
AF24	AA3-3-2-3	-	-	-	-	-	+		**1**
AF25	AA3-3-2-4	-	-			-	+		**1**
AF26	AA3-3-2-5	-	-			-	+		**1**
AF27	AA3-3-2-6	-	-			-	+		**1**
AF28	AA3-3-2-7	-	-			-	+		**1**
AF29	AA3-3-3-1	-	-	-	+	-	+		**1**
AF30	AA3-3-8-1	-	-			-			
AF31	AA3-3-8-2	-	-			-			
AF32	AA3-3-8-3	-	-			-	+		**1**
AF33	AA3-3-8-4	-	-			-	+		**1**
AF34	AA3-3-8-5	-	-			-	+		**1**
AF35	AA3-3-8-6	-	-			-			
AF36	AA3-3-8-7	-	-			-			
AF37	AA3-3-8-8	-	-			-			
AF38	AA4-3-4-1	-	-	-	-	-			
AF39	AA4-3-4-2	-	-	-	-	-			
AF40	AA8-3-2-1	-	-	+	-	-	+		**2**
AF41	AA8-3-2-2	-	-	-	-	-			
AF42	AA8-3-2-3	-	-	-	-	-	+		**1**
AF43	AA8-3-2-4	-	+			-	+		**2**
AF44	AA8-3-2-5	-	+			-			**1**
AF45	AA8-3-2-6	-	-			-	+		**1**
AF46	AA9-3-12-1	-	-	-	-	-			
AF47	AA9-3-12-2	-	-	-	-	-			
AF48	AA9-3-12-3	-	-	-	-	-			
AF49	AA9-3-12-4	-	-			-			
AF50	AA9-3-12-5	-	-			-	+		**1**
AF51	AA9-3-5-1	-	+	+	-	-	+		**3**
AF52	AA9-3-5-2	-	+	-	-	-			**1**
AF53	AA9-3-5-3	-	+			-	+		**2**

AF54	AA9-3-5-4	**-**	**+**			**-**	**+**		**2**
AF55	AA9-3-5-5	**-**	**+**			**-**	**+**		**2**
AF56	AA9-3-5-6	**-**	**+**			**-**	**+**		**2**
AF57	AA9-3-7-1	**-**	**-**	**-**	**-**	**-**	**+**		**1**
AF58	AA9-3-7-2	**-**	**-**	**+**	**-**	**-**	**+**		**2**
AF59	AA9-3-7-3	**-**	**-**	**-**	**-**	**-**	**+**		**1**
