# Supplementary material for: Atrazine induced epigenetic transgenerational inheritance of disease, lean phenotype and sperm epimutation pathology biomarkers
Source: PLoS One. 2017 Sep 20;12(9):e0184306. doi: 10.1371/journal.pone.0184306 (PMC5606923; doi:10.1371/journal.pone.0184306)
Supplement: S2 Table — (A) F3 generation control lineage males, (B) F3 generation atrazine lineage males, (C) F3 generation control lineage females, and (D) F3 generation atrazine lineage females. The animal ID and abnormal weight, BMI, adipose area (lean or obese) and adiposity are indicated with (+) or not effected (-) or not examined, (blank space). (PDF) [file pone.0184306.s008.pdf]

Supplemental Table S2
Lean and Obese Characteristics for F3 Generation Control and Atrazine Lineage Animals

(A) F3Generation Control Lineage Males

Animal ID	Weight Abnormality	BMI Abnormality	Adipocyte Area Lean	Adipocyte Area Obese	Abnormal Abdominal Adiposity
CM1	+	+	-	-	-
CM2	-	-	-	-	-
CM3	-	-	-	-	-
CM4	-	-	-	-	-
CM5	-	-	-	-	-
CM6	-	+	-	-	-
CM7	-	-	+	-	-
CM8	-	-	-	-	-
CM9	-	-	+	-	-
CM10	+	+	-	-	-
CM11	-	-	-	-	-
CM12	-	-	-	-	-
CM13	-	-			-
CM14	-	-	-	-	-
CM16	-	-	-	-	-
CM17	+	+	-	-	-
CM18	-	-	-	-	-
CM19	-	-	-	-	+
CM20					
CM21	-	-	-	-	-
CM22	-	-	-	-	-
CM23	-	-	-	-	-
CM24	-	-	-	-	-
CM25	-	-	-	-	-
CM26	-	-	-	+	+
CM27	-	-	-	-	+

(B) F3 Generation AtrazineLineage Males

Animal ID	Weight Abnormality	BMI Abnormality	Adipocyte Area Lean	Adipocyte Area Obese	Abnormal Abdominal Adiposity
AM1	-	-	-	-	-
AM2	-	-	-	-	-
AM3	-	-	-	-	-
AM4	-	-	+	-	-
AM5	-	-	-	-	-
AM6	-	-	+	-	-
AM7	-	-	-	-	
AM8	-	-	-	-	-
AM9	-	-	-	-	-
AM10	-	-	-	-	
AM11	-	-	+	-	-
AM12	-	-	-	-	-
AM13					
AM14	-	-	+	-	-
AM15	-	-	-	-	
AM16	-	-	-	-	-
AM17	-	-	-	-	-
AM18			-	-	-
AM19	+	-	-	-	+
AM20	+	-	-	+	+
AM21	-		+	-	-
AM22	-	-	-	-	
AM23	-	+	-	-	

AM24	-	-	-	-	-
AM25	-	-	-	-	-
AM26	-	-	-	-	-
AM27	-	-	+	-	-
AM28					
AM29	-		-	-	-
AM30	-	-	-	-	+
AM31	-	-	-	-	-
AM32	-	-	-	-	-
AM33	-	-	-	-	-
AM34					
AM35					
AM36	-	-	-	-	-
AM37	-	+	-	-	-
AM38	-	-	-	-	-
AM39	-	-	-	-	-
AM40	-	-	-	-	-
AM41	-	-	-	-	-
AM42	-	-	+	-	-
AM43	+	-	-	-	-
AM44	-	-	-	+	-
AM45	-	-	-	-	-
AM46			+	-	-
AM47	-	-	+	-	-
AM48	-	-	-	-	-
AM49	-	-	-	-	-
AM50	-	-	+	-	-
AM51	-	-			-
AM52	-	-	+	-	-
AM53	-	-	+	-	-
AM54	-	-	+	-	-
AM55	-	-	-	-	-

(C) F3 Generation Control Lineage Females

Animal ID	Weight Abnormality	BMI Abnormality	Adipocyte Area		Abnormal Abdominal Adipocity
			Lean	Obese	
CF1	-	-	-	-	-
CF2	-	-	-	-	-
CF3	-	-	-	-	-
CF4	-	-	-	-	-
CF5	-	-	-	+	-
CF6	-	-	-	-	-
CF7	-	-	-	-	-
CF8	-	-	-	-	-
CF9					
CF10	-	-	-	-	-
CF11	-	-	-	-	-
CF12	-	-	-	-	-
CF13	-	-	-	-	-
CF14	-	-	-	-	-
CF15	-	-	-	-	-
CF16	-	-	-	-	-
CF17	-	-	-	-	-
CF18	-	-	-	-	-
CF19	-	-	-	-	-
CF20	-	-	+	-	-
CF21	-	-	-	+	-
CF22	-	-	-	-	-
CF23	-	-	-	-	-
CF24	-	-	-	-	-

CF25	-	-	-	-	-
CF26	-	-	-	-	-
CF27	+	+	-	-	-
CF28	-	-	-	-	-
CF29	-	-	-	-	-
CF30	-	-	-	-	-
CF31	-	-	-	-	-
CF32	-	-	-	-	-
CF33	-	-	-	-	-
CF34	-	+	-	-	-
CF35	-	-			-
CF36	-	-			-
CF37	-	+			-
CF38	-	-			-
CF39	-	-			-
CF40	-	+			-
CF41	-	-			-
CF42	-	-			-
CF43	-	-			-
CF44	-	-			-
CF45	-	-			-
CF46	-	-			-
CF47	+	-			-
CF48	-	-			-
CF49	+	+			-
CF50	-	+			-
CF51	+	+			+

(D) F3 Generation Atrazine Lineage Females

Animal ID	Weight Abnormality	BMI Abnormality	Adipocyte Area Lean	Obese	Abnormal Abdominal Adipocity
AF1	-	-	-	-	-
AF2	-	-	-	-	-
AF3	-	-	+	-	+
AF4	-	-	-	-	-
AF5	+	-	+	-	-
AF6	-	-	+	-	-
AF7	-	-	+	-	-
AF8	-	-	+	-	-
AF9	-		+	-	-
AF10	-	-	-	-	-
AF11	-	-	-	-	-
AF12	+	-	-	-	-
AF13	-	-	+	-	-
AF14	-	-	-	-	-
AF15	-	-	-	-	-
AF16	-	-	-	-	-
AF17	+	-	-	-	-
AF18	-	-	+	-	-
AF19					
AF20					
AF21	-	-	+	-	-
AF22	-	-			+
AF23	-				+
AF24	-		+	-	-
AF25	-		+	-	-
AF26	-		+	-	-
AF27	-	-	+	-	-
AF28	-	-	+	-	-
AF29	-	+	+	-	-

AF30	-	-	-	-	-
AF31	-	-	-	-	-
AF32	-	-	+	-	-
AF33	+	+	+	-	+
AF34	-	-	+	-	-
AF35					
AF36					
AF37					
AF38	-	-	-	-	-
AF39	-	-	-	-	-
AF40	+	-	+	-	+
AF41	-	-	-	-	-
AF42	-	+	+	-	-
AF43	-	+	-	-	+
AF44	-	-	-	-	-
AF45	-	-	+	-	-
AF46	-	-	-	-	-
AF47	-	-	-	-	-
AF48	-	-	-	-	-
AF49	-	-	-	-	-
AF50	-	-	+	-	-
AF51	-	-	+	-	-
AF52	-	-	-	-	-
AF53	+	-	+	-	+
AF54	-	-	+	-	-
AF55	-	-	+	-	-
AF56	-	-	+	-	-
AF57	-	-	+	-	-
AF58	-	-	+	-	+
AF59	-	-	+	-	-
