# Supplementary material for: Atrazine induced epigenetic transgenerational inheritance of disease, lean phenotype and sperm epimutation pathology biomarkers
Source: PLoS One. 2017 Sep 20;12(9):e0184306. doi: 10.1371/journal.pone.0184306 (PMC5606923; doi:10.1371/journal.pone.0184306)
Supplement: S3 Table — The DMR name, chromosome, start site, length (bp), number # significant windows, minimum p-value, CpG number, CpG % density, associated gene and gene category are presented. (PDF) [file pone.0184306.s009.pdf]

Supplemental Table S3
DMR Table F1 Generation DMR ($p<10^{-6}$) List

DMR Name	Chr	Start	Length	# SigWin	Min p-value	CpG Num	CpG (%) Density	Associated Gene	Gene Category
DMR1:2642601	1	2642601	600	1	5.05E-07	19	3.16		
DMR1:8150501	1	8150501	1500	1	4.58E-07	15	1		
DMR1:11132701	1	11132701	800	1	5.47E-07	3	0.37		
DMR1:11903501	1	11903501	10600	8	3.64E-08	1283	12.1	AABR07000390.1	Unknown
DMR1:11963601	1	11963601	14300	7	7.28E-12	1432	10.01	AABR07000398.2	Unknown
DMR1:29563601	1	29563601	400	1	1.14E-07	7	1.75		
DMR1:34971001	1	34971001	300	1	9.37E-07	4	1.33		
DMR1:36286501	1	36286501	1300	1	6.09E-09	10	0.76	Nsun2	Epigenetic
DMR1:48351301	1	48351301	300	1	7.75E-07	6	2	Slc22a2	Transport
DMR1:52506001	1	52506001	300	1	4.50E-07	10	3.33	Pde10a	Signaling
DMR1:54035301	1	54035301	8700	1	8.03E-07	72	0.82	AABR07001592.1	Unknown
DMR1:54118901	1	54118901	300	1	2.03E-08	1	0.33	AABR07001599.1	Unknown
DMR1:54414601	1	54414601	1200	1	9.78E-07	18	1.5		
DMR1:54979001	1	54979001	1600	1	9.31E-07	18	1.12		
DMR1:60151601	1	60151601	2300	1	1.25E-07	18	0.78		
DMR1:66676801	1	66676801	1000	1	6.57E-07	11	1.1	Vom1r57	Receptor
DMR1:70978501	1	70978501	700	1	9.45E-07	2	0.28		
DMR1:71518501	1	71518501	4600	1	1.11E-07	35	0.76		
DMR1:75307501	1	75307501	2100	1	9.53E-07	12	0.57	Vom1r61	Unknown
DMR1:79353601	1	79353601	700	4	5.49E-17	1	0.14	LOC102557244	Unknown
DMR1:91668201	1	91668201	200	1	6.82E-08	2	1	Cep89	Unknown
DMR1:93789501	1	93789501	1000	1	9.57E-08	1	0.1		
DMR1:102187801	1	102187801	2100	1	2.91E-07	49	2.33	Abcc8	Transport
DMR1:102318401	1	102318401	1600	1	2.82E-07	29	1.81	Otog	Unknown
DMR1:102764601	1	102764601	2700	2	3.83E-09	105	3.88	Hps5	Protein Binding
DMR1:103732801	1	103732801	3200	2	4.44E-07	29	0.9	Mrgprx2	Receptor
DMR1:108755301	1	108755301	300	1	3.00E-07	2	0.66		
DMR1:123394201	1	123394201	2700	1	2.85E-12	21	0.77		
DMR1:135433401	1	135433401	2100	1	6.76E-07	39	1.85		
DMR1:139606701	1	139606701	1400	1	5.57E-07	19	1.35	AABR07004375.1	Unknown
DMR1:151293301	1	151293301	2000	1	7.06E-08	8	0.4		
DMR1:157584701	1	157584701	2000	1	2.24E-07	13	0.65	Rab30	Signaling
DMR1:158851201	1	158851201	400	1	7.75E-07	7	1.75		
DMR1:163839701	1	163839701	6400	1	3.70E-07	133	2.07	Uvrag	Unknown
DMR1:165024101	1	165024101	300	1	8.79E-07	5	1.66	Chrdl2	Signaling
DMR1:172496401	1	172496401	2400	1	6.75E-08	10	0.41		
DMR1:176142601	1	176142601	300	1	2.15E-08	9	3		
DMR1:176573701	1	176573701	300	1	7.52E-10	1	0.33	Galnt18	Unknown
DMR1:179683701	1	179683701	4600	1	4.10E-08	12	0.26		
DMR1:190724501	1	190724501	2700	1	8.26E-08	41	1.51	Vwa3a	Unknown
DMR1:204404001	1	204404001	200	2	2.98E-07	0	0		
DMR1:206290201	1	206290201	1900	1	3.88E-07	10	0.52	Adam12	Protease
DMR1:217100601	1	217100601	500	1	6.79E-07	3	0.6		
DMR1:218109901	1	218109901	100	1	3.85E-07	0	0	Ccnd1	Cell Cycle
DMR1:218234501	1	218234501	600	1	9.60E-07	1	0.16		
DMR1:237950301	1	237950301	1400	1	1.22E-07	17	1.21		
DMR1:247878501	1	247878501	500	1	1.42E-08	3	0.6	Mlana	Metabolism
DMR1:248084901	1	248084901	900	1	1.38E-09	12	1.33		
DMR1:249349601	1	249349601	2900	1	5.01E-07	44	1.51		
DMR2:5612201	2	5612201	3800	1	7.35E-08	33	0.86		
DMR2:8362401	2	8362401	1500	1	7.75E-07	14	0.93		
DMR2:16161901	2	16161901	3300	1	5.09E-08	25	0.75		
DMR2:19305301	2	19305301	500	1	4.97E-07	3	0.6		
DMR2:25312001	2	25312001	2100	1	5.10E-08	34	1.61		
DMR2:33267901	2	33267901	1400	1	6.12E-07	43	3.07		
DMR2:39171801	2	39171801	1200	1	1.90E-07	3	0.25		
DMR2:49140901	2	49140901	1500	1	8.77E-07	9	0.6	Parp8	Unknown
DMR2:57510901	2	57510901	1900	1	1.87E-08	34	1.78	Nipbl	Epigenetic
DMR2:64355301	2	64355301	1300	1	2.63E-07	10	0.76		
DMR2:64938001	2	64938001	700	1	8.10E-07	2	0.28		
DMR2:68241801	2	68241801	400	1	6.56E-07	4	1	AABR07008646.1	Unknown
DMR2:74865301	2	74865301	500	1	1.28E-07	2	0.4		

DMR2:77539001	2	77539001	2500	1	3.43E-11	14	0.56		
DMR2:78492301	2	78492301	3100	1	2.69E-07	20	0.64		
DMR2:122126901	2	122126901	400	1	1.66E-07	2	0.5		
DMR2:130557301	2	130557301	800	1	8.43E-07	3	0.37		
DMR2:135140601	2	135140601	1600	1	4.60E-07	11	0.68		
DMR2:142111401	2	142111401	1000	1	5.59E-07	20	2		
DMR2:146380201	2	146380201	300	2	2.23E-08	14	4.66		
DMR2:147464401	2	147464401	3700	1	8.91E-07	43	1.16	AC129365.1	Unknown
DMR2:165986001	2	165986001	900	2	1.56E-13	9	1		
DMR2:166767301	2	166767301	1000	1	2.33E-07	4	0.4	AABR07011705.1	Unknown
DMR2:169651901	2	169651901	500	1	4.68E-07	0	0		
DMR2:170957601	2	170957601	1100	2	1.24E-10	6	0.54		
DMR2:187158101	2	187158101	400	2	7.89E-08	11	2.75	Ntrk1	Receptor
DMR2:194567001	2	194567001	1200	1	2.68E-07	1	0.08	LOC100911679	Unknown
DMR2:195020301	2	195020301	1300	1	1.56E-07	8	0.61		
DMR2:197209101	2	197209101	3800	1	6.70E-07	168	4.42	AABR07012535.1	Unknown
DMR2:206002301	2	206002301	3400	1	1.27E-07	31	0.91		
DMR2:215440101	2	215440101	700	1	8.00E-07	7	1		
DMR2:217714101	2	217714101	1400	1	7.27E-07	8	0.57	AABR07012994.1	Unknown
DMR2:221633501	2	221633501	1100	1	3.29E-07	8	0.72		
DMR2:228601001	2	228601001	7100	1	9.57E-08	88	1.23		
DMR2:231263601	2	231263601	300	1	3.40E-07	3	1	Ank2	Cytoskeleton
DMR2:233157401	2	233157401	1400	1	2.27E-07	9	0.64		
DMR2:236426501	2	236426501	2800	1	1.68E-07	53	1.89	Cyp2u1	Metabolism
DMR2:250642801	2	250642801	1300	1	6.78E-07	14	1.07	15-Sep	Unknown
DMR2:252166001	2	252166001	1400	1	4.53E-08	13	0.92	Lpar3	Receptor
DMR2:263632301	2	263632301	1300	1	1.01E-08	22	1.69	Negr1	Extracellular Matrix;Unknown
DMR3:2655901	3	2655901	6300	1	1.43E-07	178	2.82	Abca2	Transport;Metabolism
DMR3:9610501	3	9610501	700	1	6.31E-07	1	0.14		
DMR3:13693601	3	13693601	600	1	8.65E-07	8	1.33	Mapkap1	Signaling
DMR3:14324101	3	14324101	1100	1	1.50E-07	7	0.63	Cntrl	Unknown
DMR3:17045501	3	17045501	2300	1	2.40E-07	23	1		
DMR3:33581701	3	33581701	300	1	1.63E-08	8	2.66		
DMR3:33743201	3	33743201	400	1	9.92E-08	7	1.75	Epc2	Epigenetic
DMR3:36093501	3	36093501	400	1	7.98E-08	5	1.25		
DMR3:37962301	3	37962301	2800	1	4.58E-07	27	0.96	Cacnb4	Transport
DMR3:41242701	3	41242701	1000	2	7.76E-08	8	0.8		
DMR3:64736501	3	64736501	800	1	6.61E-07	8	1	Cwc22	Translation
DMR3:77823601	3	77823601	1200	1	6.02E-08	3	0.25	Olr675	Receptor
DMR3:111160701	3	111160701	500	1	4.88E-07	7	1.4	Chac1	Metabolism
DMR3:118675601	3	118675601	500	1	3.52E-07	8	1.6	Atp8b4	Transport
DMR3:121117201	3	121117201	2800	1	5.94E-07	28	1	LOC499886	Unknown
DMR3:123071201	3	123071201	1100	1	2.33E-07	43	3.9	Ptpra	Signaling
DMR3:124286301	3	124286301	1900	1	3.24E-07	16	0.84		
DMR3:130229201	3	130229201	300	1	5.90E-07	0	0		
DMR3:136826501	3	136826501	4600	1	8.89E-08	43	0.93	Kif16b	Cytoskeleton;Unknown
DMR3:144245501	3	144245501	3600	1	4.27E-08	30	0.83		
DMR3:145337001	3	145337001	800	2	8.83E-15	0	0		
DMR3:147378801	3	147378801	3100	1	5.88E-07	60	1.93	Rspo4	Growth Factors & Cytokines
DMR3:150169401	3	150169401	700	1	3.81E-07	12	1.71		
DMR3:162107601	3	162107601	200	1	1.74E-07	1	0.5	5S_rRNA	Translation;Transport
DMR3:167248101	3	167248101	1200	1	4.75E-08	7	0.58		
DMR3:171427901	3	171427901	1700	1	9.79E-07	40	2.35		
DMR3:175596201	3	175596201	2300	1	4.49E-08	35	1.52	Lama5	Extracellular Matrix
DMR4:6574701	4	6574701	2200	1	1.06E-08	50	2.27	Prkag2	Signaling
DMR4:26098201	4	26098201	1200	1	9.77E-07	27	2.25	Cdk14	Cell Cycle
DMR4:30750801	4	30750801	200	1	7.42E-07	0	0		
DMR4:37577401	4	37577401	100	1	5.47E-08	0	0		
DMR4:41227901	4	41227901	400	1	7.07E-07	3	0.75		
DMR4:52048101	4	52048101	1100	1	7.45E-07	6	0.54		
DMR4:65919901	4	65919901	4400	1	6.53E-07	59	1.34	RGD1306271	Unknown
DMR4:86249201	4	86249201	3300	1	8.18E-08	24	0.72		
DMR4:96343701	4	96343701	900	2	5.05E-18	4	0.44		
DMR4:97393901	4	97393901	2500	1	7.79E-07	17	0.68		
DMR4:98089301	4	98089301	1400	1	6.15E-08	16	1.14	Il12rb2	Receptor
DMR4:106902001	4	106902001	3000	1	7.73E-07	60	2		
DMR4:110671701	4	110671701	1400	1	5.13E-07	10	0.71		

DMR4:116653401	4	116653401	1400	1	6.68E-07	13	0.92	Exoc6b	Transport
DMR4:117693301	4	117693301	1200	1	8.22E-08	19	1.58	Figla	Transcription
DMR4:118037601	4	118037601	2000	1	8.91E-07	23	1.15	Tgfa	Growth Factors & Cytokines
DMR4:118117401	4	118117401	1400	1	3.25E-07	8	0.57		
DMR4:127462301	4	127462301	1200	2	2.94E-07	17	1.41		
DMR4:137584601	4	137584601	800	1	5.85E-08	6	0.75		
DMR4:147849101	4	147849101	1100	1	9.60E-07	18	1.63	Plxnd1	Receptor;Epigenetic
DMR4:149245701	4	149245701	300	1	5.58E-07	0	0		
DMR4:157992301	4	157992301	2200	1	3.71E-07	43	1.95	Cd9	Extracellular Matrix
DMR4:166661701	4	166661701	2500	2	3.95E-07	171	6.84	AABR07062274.1	Unknown
DMR4:170359701	4	170359701	800	1	9.82E-07	49	6.12	LOC500350	Unknown
DMR4:170706801	4	170706801	400	1	2.11E-08	1	0.25	Gucy2c	Metabolism
DMR4:171634001	4	171634001	600	1	7.78E-07	2	0.33		
DMR4:177340601	4	177340601	4300	1	5.11E-07	73	1.69	C2cd5	Signaling
DMR4:180515101	4	180515101	1800	1	9.58E-07	15	0.83	AABR07062512.1	Unknown
DMR4:180677501	4	180677501	400	1	5.74E-07	7	1.75	AABR07062513.1	Unknown
DMR4:181916901	4	181916901	800	1	7.10E-07	10	1.25	Ccdc91	Unknown
DMR4:182929701	4	182929701	300	1	7.60E-08	3	1		
DMR5:191301	5	191301	1000	1	3.47E-07	6	0.6	LOC102557117	Unknown
DMR5:7637101	5	7637101	1200	1	4.02E-08	14	1.16	Prex2	Signaling
DMR5:21996001	5	21996001	800	1	3.60E-07	10	1.25		
DMR5:31829201	5	31829201	2200	1	3.22E-07	14	0.63		
DMR5:36846401	5	36846401	700	1	4.23E-09	6	0.85		
DMR5:39072301	5	39072301	200	1	1.25E-07	0	0	Klhl32	Transcription
DMR5:47127201	5	47127201	2400	1	6.55E-07	15	0.62		
DMR5:50369101	5	50369101	4700	1	3.14E-07	173	3.68	Cga	Hormone;Transcription;Unknown
DMR5:63632301	5	63632301	1800	1	2.71E-07	48	2.66		
DMR5:64407401	5	64407401	900	1	5.43E-07	19	2.11	Tmeff1	Receptor;Immune
DMR5:79600701	5	79600701	4500	1	9.60E-08	50	1.11		
DMR5:87352501	5	87352501	1200	1	7.61E-07	5	0.41	RGD1560539	Unknown
DMR5:91126801	5	91126801	11200	2	1.54E-09	1418	12.66	AABR07048785.1	Unknown
DMR5:104808301	5	104808301	2500	1	2.81E-08	46	1.84	Fam154a	Unknown
DMR5:105404901	5	105404901	300	1	4.28E-07	7	2.33	Slc24a2	Transport
DMR5:120627601	5	120627601	500	1	6.40E-07	2	0.4	Leprot	Receptor
DMR5:126837301	5	126837301	300	1	4.04E-08	11	3.66	Lrrc42	Unknown
DMR5:129430601	5	129430601	600	1	2.49E-07	3	0.5	AABR07049579.1	Unknown
DMR5:149122801	5	149122801	500	1	4.77E-07	8	1.6		
DMR5:151994801	5	151994801	200	1	7.92E-09	1	0.5		
DMR5:158009701	5	158009701	300	1	2.34E-08	4	1.33		
DMR5:161707501	5	161707501	200	1	1.72E-07	0	0		
DMR5:169087701	5	169087701	400	1	8.37E-07	6	1.5		
DMR5:169746301	5	169746301	5600	1	1.32E-07	131	2.33	Nphp4	Development
DMR5:172456301	5	172456301	1900	1	9.94E-07	19	1		
DMR6:1686001	6	1686001	500	2	3.40E-12	15	3	Qpct	Metabolism
DMR6:5772501	6	5772501	6500	1	4.92E-07	62	0.95		
DMR6:10465701	6	10465701	2200	2	5.17E-07	96	4.36		
DMR6:10805601	6	10805601	11000	2	1.87E-08	452	4.1		
DMR6:11744501	6	11744501	1200	1	5.41E-12	10	0.83		
DMR6:26168801	6	26168801	3800	1	6.35E-07	58	1.52		
DMR6:29316201	6	29316201	500	1	4.50E-07	26	5.2	Klhl29	Transcription
DMR6:29568101	6	29568101	400	1	5.79E-07	1	0.25		
DMR6:30628101	6	30628101	7300	11	2.05E-10	774	10.6	AABR07063421.1	Unknown
DMR6:30638001	6	30638001	1300	3	1.87E-08	144	11.07	AABR07063421.1	Unknown
DMR6:30640301	6	30640301	2100	3	9.26E-14	180	8.57	AABR07063421.1	Unknown
DMR6:31048801	6	31048801	2100	5	4.86E-11	169	8.04	AABR07063462.1	Unknown
DMR6:42127501	6	42127501	300	1	7.15E-07	6	2		
DMR6:57084401	6	57084401	900	1	1.38E-07	8	0.88	Agmo	Metabolism
DMR6:68616901	6	68616901	3200	1	9.32E-07	25	0.78		
DMR6:100953401	6	100953401	3400	1	7.16E-07	40	1.17		
DMR6:105146801	6	105146801	3700	1	7.06E-07	44	1.18	Adam4	Proteolysis
DMR6:107693101	6	107693101	1500	1	1.26E-07	27	1.8		
DMR6:119579301	6	119579301	200	1	7.54E-10	0	0	Flrt2	Cytoskeleton
DMR6:123872001	6	123872001	1500	1	3.35E-07	30	2	Efcab11	Signaling
DMR6:130845901	6	130845901	400	1	2.49E-07	0	0		
DMR6:132111701	6	132111701	1100	1	6.84E-09	20	1.81	AABR07065498.1	Unknown;Cell Cycle
DMR6:142893001	6	142893001	3800	1	5.56E-08	34	0.89	NA	NA
DMR6:143713301	6	143713301	100	1	5.55E-08	1	1		

DMR7:710701	7	710701	4400	2	3.82E-09	253	5.75	AABR07055221.1	Unknown
DMR7:2377801	7	2377801	1400	1	2.26E-07	8	0.57		
DMR7:5876901	7	5876901	200	1	2.80E-07	1	0.5		
DMR7:11183401	7	11183401	1300	2	1.64E-07	6	0.46	Nfic;Smim24	Transcription;Unknown;Metabolism
DMR7:11376501	7	11376501	2600	2	1.69E-07	39	1.5	Nmrk2	Signaling;Development
DMR7:15200501	7	15200501	400	1	5.33E-07	6	1.5	Cyp4f1	Metabolism
DMR7:16975301	7	16975301	10100	1	3.96E-07	111	1.09	Vom2r54	Receptor
DMR7:22385101	7	22385101	2900	2	1.14E-09	17	0.58		
DMR7:27264201	7	27264201	1800	1	6.35E-07	28	1.55	LOC362863	Unknown
DMR7:44358301	7	44358301	6600	1	1.93E-08	66	1		
DMR7:54958601	7	54958601	3400	1	3.88E-07	30	0.88		
DMR7:56599101	7	56599101	400	1	2.15E-07	2	0.5		
DMR7:56608001	7	56608001	900	1	1.31E-07	2	0.22		
DMR7:59324601	7	59324601	300	1	3.32E-08	14	4.66	Ptprr	Signaling
DMR7:59369601	7	59369601	2300	1	5.51E-07	43	1.86	Ptprb	Signaling
DMR7:63962901	7	63962901	900	1	1.40E-07	4	0.44	Srgap1	Signaling
DMR7:77649801	7	77649801	1000	1	4.23E-07	8	0.8	5S_rRNA	Translation
DMR7:83091001	7	83091001	200	1	4.20E-07	0	0		
DMR7:85690301	7	85690301	600	1	1.45E-07	2	0.33		
DMR7:98824901	7	98824901	2000	1	1.78E-08	40	2	Ndufb9	Metabolism;Cytoskeleton
DMR7:99521601	7	99521601	7400	1	2.51E-07	448	6.05		
DMR7:115169901	7	115169901	1500	3	8.85E-13	12	0.8		
DMR7:119768001	7	119768001	500	1	9.18E-10	17	3.4	Sstr3	Receptor
DMR7:123634401	7	123634401	1200	1	1.03E-07	19	1.58	AC107527.1	Unknown;Electron Transport
DMR7:124188501	7	124188501	300	1	9.75E-07	5	1.66	Arfgap3	Signaling
DMR7:134893901	7	134893901	800	2	6.93E-09	15	1.87		
DMR7:137336101	7	137336101	1400	1	3.98E-07	21	1.5	Ano6	Signaling
DMR7:143623001	7	143623001	2300	1	6.06E-07	24	1.04	Krt18	Cytoskeleton;Unknown
DMR7:145070701	7	145070701	1300	1	8.74E-07	14	1.07	Nckap1l	Development;Unknown
DMR8:3366701	8	3366701	1800	1	2.87E-08	12	0.66		
DMR8:14259301	8	14259301	600	2	7.61E-08	27	4.5	Slc36a4	Transport
DMR8:26556001	8	26556001	300	1	4.32E-08	3	1		
DMR8:33594601	8	33594601	400	1	4.29E-07	4	1	Fli1	Transcription
DMR8:36532901	8	36532901	700	1	8.47E-07	6	0.85		
DMR8:43809901	8	43809901	400	1	4.88E-07	5	1.25	Olr1331	Receptor
DMR8:47236701	8	47236701	500	1	7.84E-08	42	8.4		
DMR8:47451001	8	47451001	400	1	8.23E-08	1	0.25	Pou2f3	Transcription
DMR8:50692001	8	50692001	600	1	8.24E-07	3	0.5		
DMR8:52438601	8	52438601	1400	1	8.12E-07	37	2.64		
DMR8:59009901	8	59009901	500	1	9.88E-08	1	0.2	Dmxl2	Unknown
DMR8:82747301	8	82747301	1600	1	9.90E-07	29	1.81		
DMR8:88525301	8	88525301	200	2	6.46E-09	1	0.5		
DMR8:90673401	8	90673401	3200	1	4.45E-08	43	1.34	Hmgn3	Epigenetic
DMR8:95991201	8	95991201	300	1	2.78E-07	9	3	Nt5e	Signaling
DMR8:98699401	8	98699401	900	1	4.87E-09	5	0.55		
DMR8:114333101	8	114333101	600	2	7.37E-08	12	2	Pik3r4	Signaling
DMR8:116876101	8	116876101	1000	1	9.99E-08	32	3.2	Apeh	Protease;Unknown;Extracellular Matrix
DMR8:122529201	8	122529201	700	1	1.35E-07	9	1.28	Ccr4	Unknown
DMR8:127299301	8	127299301	500	1	4.18E-07	2	0.4	Itga9	Extracellular Matrix
DMR8:127653101	8	127653101	400	1	4.47E-09	5	1.25		
DMR8:128295701	8	128295701	700	1	6.90E-09	2	0.28	Scn10a	Transport
DMR8:130649401	8	130649401	500	2	2.34E-08	5	1		
DMR8:132125801	8	132125801	5200	1	9.15E-07	42	0.8	Tgm4	Metabolism
DMR9:10630901	9	10630901	1600	1	8.92E-07	75	4.68	Ptprs	Unknown;Signaling
DMR9:12360401	9	12360401	3000	1	3.28E-07	35	1.16		
DMR9:28479901	9	28479901	500	1	9.93E-07	11	2.2	Rims1	Signaling
DMR9:29526701	9	29526701	300	1	6.04E-08	15	5		
DMR9:43491301	9	43491301	900	1	4.41E-07	10	1.11	AABR07067403.1	Unknown
DMR9:49017801	9	49017801	400	1	2.32E-07	4	1	AABR07067491.1	Unknown
DMR9:49143801	9	49143801	200	1	1.84E-07	1	0.5	AABR07067491.2	Unknown
DMR9:49853101	9	49853101	400	1	6.84E-07	13	3.25	Tgfbrap1	Signaling
DMR9:59339801	9	59339801	400	1	7.56E-07	14	3.5		
DMR9:67774601	9	67774601	300	1	6.36E-09	11	3.66	Icos	Immune
DMR9:74504801	9	74504801	1500	1	3.78E-08	59	3.93		
DMR9:82658101	9	82658101	600	1	4.32E-08	8	1.33	Asic4	Transport
DMR9:96730901	9	96730901	600	2	4.38E-09	9	1.5	Agap1	Signaling
DMR9:105433001	9	105433001	1100	1	8.51E-07	12	1.09		

DMR9:111721901	9	111721901	1400	1	2.07E-07	20	1.42	Fer	Signaling
DMR9:117790701	9	117790701	2000	1	4.65E-08	43	2.15	Akain1	Signaling
DMR9:118974401	9	118974401	400	1	9.45E-07	5	1.25	Dlgap1	Signaling
DMR10:5280001	10	5280001	2000	1	8.13E-07	27	1.35		
DMR10:11291001	10	11291001	1800	2	2.43E-08	71	3.94	Srl	
DMR10:11948001	10	11948001	3100	1	5.49E-07	38	1.22	Olr1356	Receptor
DMR10:21797001	10	21797001	1500	1	7.32E-07	9	0.6		
DMR10:53202301	10	53202301	900	1	4.21E-07	6	0.66		
DMR10:56141601	10	56141601	3100	1	5.05E-07	28	0.9	Dnah2	Cytoskeleton
DMR10:68968701	10	68968701	2400	1	2.40E-07	25	1.04	Mir6327	Epigenetic
DMR10:76826001	10	76826001	900	1	2.62E-07	14	1.55		
DMR10:82711801	10	82711801	3400	1	2.70E-07	40	1.17		
DMR10:85533101	10	85533101	400	1	1.42E-09	2	0.5		
DMR10:85706101	10	85706101	400	1	5.14E-09	18	4.5	Cwc25	Translation;Unknown
DMR10:86825801	10	86825801	500	1	1.33E-07	17	3.4	Cdc6	Cell Cycle
DMR10:88798501	10	88798501	3500	1	1.84E-07	50	1.42	Stat5a	Transcription
DMR10:97580601	10	97580601	400	1	3.49E-07	7	1.75	Rgs9	Signaling
DMR10:105063901	10	105063901	800	1	8.04E-07	16	2	Ten1	Unknown
DMR10:108326201	10	108326201	2500	2	5.06E-08	52	2.08	Tbc1d16	Signaling
DMR10:111398801	10	111398801	3700	1	9.96E-08	23	0.62	AABR07030977.1	Unknown
DMR11:891201	11	891201	800	1	3.44E-07	6	0.75		
DMR11:1694801	11	1694801	500	1	7.94E-07	0	0		
DMR11:4916201	11	4916201	200	1	2.18E-07	0	0		
DMR11:24052401	11	24052401	400	1	8.77E-07	1	0.25	AABR07033492.2	Unknown
DMR11:31862301	11	31862301	2100	1	6.44E-08	5	0.23	Cryzl1	Metabolism
DMR11:32846601	11	32846601	600	1	3.22E-08	4	0.66	Runx1	Transcription
DMR11:33105101	11	33105101	2600	1	2.18E-07	51	1.96		
DMR11:39445701	11	39445701	200	1	6.52E-11	1	0.5	AABR07033817.1	Unknown
DMR11:42610801	11	42610801	2200	1	7.44E-07	20	0.9	Epha6	Receptor
DMR11:43270601	11	43270601	1900	1	3.34E-07	15	0.78	Olr1538	Receptor
DMR11:56101401	11	56101401	300	1	2.68E-07	1	0.33	AABR07034182.1	Unknown
DMR11:63478401	11	63478401	300	1	7.21E-07	1	0.33		
DMR11:65326301	11	65326301	1900	1	3.81E-08	20	1.05	Gpr156	Receptor
DMR11:65684201	11	65684201	1100	1	6.85E-08	6	0.54		
DMR11:66698401	11	66698401	300	1	9.51E-07	2	0.66	Polq	Transcription
DMR11:78723501	11	78723501	600	1	8.47E-07	0	0	Tprg1	Unknown
DMR11:82427601	11	82427601	3100	1	1.61E-07	49	1.58		
DMR11:90448601	11	90448601	200	2	2.87E-18	0	0		
DMR12:2250901	12	2250901	1800	1	1.03E-07	34	1.88	AABR07034964.1	Unknown;Immune
DMR12:6268901	12	6268901	400	1	1.10E-07	5	1.25	Wdr95	Unknown
DMR12:8094801	12	8094801	700	1	2.02E-08	21	3	Slc7a1	Transport
DMR12:8461501	12	8461501	800	1	7.20E-07	10	1.25		
DMR12:8749301	12	8749301	400	2	3.41E-08	14	3.5	Slc46a3	Transport;Proteolysis
DMR12:10648301	12	10648301	500	1	3.50E-07	4	0.8	Cyp3a18	Unknown;Metabolism
DMR12:16392201	12	16392201	1400	1	6.98E-07	24	1.71	Snx8	Transport;Metabolism;Epigenetic
DMR12:16730501	12	16730501	1400	2	1.10E-07	19	1.35		
DMR12:30588901	12	30588901	1800	1	2.82E-08	15	0.83		
DMR12:32079501	12	32079501	3200	1	2.47E-07	42	1.31	Tmem132d	Unknown
DMR12:34566701	12	34566701	600	1	2.95E-07	3	0.5		
DMR12:38010501	12	38010501	5600	1	5.03E-07	113	2.01	Hip1r	Cytoskeleton;Proteolysis
DMR12:42940301	12	42940301	2700	1	9.29E-07	43	1.59		
DMR12:44561001	12	44561001	1000	2	2.34E-08	22	2.2	Ksr2	Signaling
DMR12:45946801	12	45946801	1100	1	1.83E-08	19	1.72	AABR07036514.1	Unknown
DMR12:46445401	12	46445401	4100	1	7.97E-07	85	2.07	Cit	Signaling
DMR12:46966301	12	46966301	800	1	8.28E-07	8	1	AABR07036532.1	Unknown
DMR13:2801501	13	2801501	1800	1	7.14E-09	11	0.61		
DMR13:11104801	13	11104801	100	1	1.05E-08	0	0		
DMR13:18900201	13	18900201	3800	1	2.19E-07	42	1.1		
DMR13:19612501	13	19612501	3200	1	3.72E-07	32	1		
DMR13:20176501	13	20176501	1400	1	6.65E-08	7	0.5		
DMR13:24384901	13	24384901	1700	1	3.16E-07	7	0.41		
DMR13:34314801	13	34314801	600	1	4.68E-07	1	0.16		
DMR13:35227701	13	35227701	1700	1	4.12E-08	14	0.82		
DMR13:39484001	13	39484001	2300	1	4.57E-07	17	0.73	Dpp10	Transport
DMR13:41312501	13	41312501	200	1	7.95E-07	2	1		
DMR13:45326101	13	45326101	3100	1	1.64E-07	50	1.61	Cxcr4	Unknown;Receptor
DMR13:48911701	13	48911701	1500	1	3.16E-07	12	0.8	Cdk18	Cell Cycle

DMR13:49597001	13	49597001	1400	2	1.75E-07	33	2.35	AABR07072325.1	Unknown
DMR13:50557201	13	50557201	3000	1	2.20E-08	67	2.23	Golt1a	Golgi;Unknown
DMR13:50753801	13	50753801	3100	1	2.02E-07	50	1.61	Prelp	Extracellular Matrix
DMR13:57122301	13	57122301	1100	1	7.30E-07	5	0.45	Kcnt2	Transport
DMR13:69384201	13	69384201	4300	1	7.13E-09	99	2.3	RGD1309104	Unknown
DMR13:70521301	13	70521301	500	1	7.42E-07	4	0.8	Nmnat2	Metabolism
DMR13:72610301	13	72610301	200	1	1.16E-07	5	2.5		
DMR13:91616801	13	91616801	600	1	4.11E-07	7	1.16		
DMR13:100011801	13	100011801	1600	1	4.70E-07	17	1.06		
DMR13:100867401	13	100867401	3400	1	3.11E-08	196	5.76	Capn2	Protease;DNA Repair
DMR13:101295501	13	101295501	700	1	2.46E-07	14	2	Susd4	Development
DMR13:102603301	13	102603301	900	2	1.85E-08	7	0.77		
DMR13:103639401	13	103639401	500	1	4.87E-07	2	0.4		
DMR13:106560601	13	106560601	1900	1	4.95E-08	36	1.89	Esrrg	Receptor;Transcription
DMR13:110328201	13	110328201	1700	1	1.04E-07	26	1.52		
DMR13:110988001	13	110988001	800	1	9.08E-07	3	0.37	Kcnh1	Transport
DMR14:3771901	14	3771901	700	1	4.70E-07	14	2		
DMR14:4700401	14	4700401	2500	1	4.37E-07	23	0.92		
DMR14:5321701	14	5321701	3300	1	7.58E-08	51	1.54	Lrrc8c	Unknown
DMR14:7234101	14	7234101	4100	1	5.11E-07	98	2.39	Aff1	Transcription
DMR14:8165001	14	8165001	300	1	4.70E-07	0	0	Mapk10	Signaling
DMR14:8238201	14	8238201	1100	1	6.88E-07	25	2.27	Mapk10	Signaling
DMR14:10690101	14	10690101	800	1	7.90E-08	8	1	Plac8	Development;Unknown
DMR14:12031401	14	12031401	1500	1	3.57E-07	10	0.66	Rasgef1b	Signaling
DMR14:13475101	14	13475101	900	1	4.82E-07	3	0.33	AABR07014384.1	Unknown
DMR14:15225201	14	15225201	600	2	2.52E-10	25	4.16		
DMR14:17715301	14	17715301	1000	1	9.90E-07	28	2.8		
DMR14:18535601	14	18535601	2400	1	2.72E-11	19	0.79	Areg	Growth Factors & Cytokines
DMR14:20615501	14	20615501	200	1	9.05E-10	2	1	Slc4a4	Transport
DMR14:22236801	14	22236801	1200	1	2.49E-07	58	4.83	Ugt2a3	Metabolism
DMR14:33231501	14	33231501	4200	1	4.72E-07	19	0.45	AC097433.1	Unknown
DMR14:39036101	14	39036101	200	1	4.68E-07	1	0.5	Gabrb1	Receptor
DMR14:39390401	14	39390401	3600	1	9.18E-08	121	3.36		
DMR14:42366701	14	42366701	4500	1	3.48E-08	68	1.51		
DMR14:46516401	14	46516401	2000	4	4.63E-09	137	6.85	AABR07015055.2	Unknown
DMR14:46521201	14	46521201	7000	9	5.11E-12	738	10.54	AABR07015055.2	Unknown
DMR14:46588201	14	46588201	900	2	1.78E-07	97	10.77	LOC257642	Unknown
DMR14:46590101	14	46590101	500	3	3.09E-12	49	9.8	LOC257642	Unknown
DMR14:46635101	14	46635101	21500	7	3.52E-09	2269	10.55	Rn5-8s	Translation;Unknown
DMR14:46679301	14	46679301	9300	1	2.56E-07	1040	11.18	AABR07015089.1	Unknown
DMR14:50841701	14	50841701	200	1	1.12E-10	0	0		
DMR14:51784401	14	51784401	1700	1	9.36E-08	11	0.64		
DMR14:54501101	14	54501101	300	1	1.25E-07	14	4.66		
DMR14:56518201	14	56518201	200	2	6.81E-07	0	0		
DMR14:60233701	14	60233701	300	1	9.69E-07	4	1.33	Sel1l3	Signaling
DMR14:67192901	14	67192901	2300	1	8.54E-07	30	1.3		
DMR14:67731701	14	67731701	2900	1	7.64E-07	10	0.34		
DMR14:78206901	14	78206901	400	1	5.27E-09	9	2.25	Evc	Development
DMR14:81228201	14	81228201	200	1	3.72E-07	6	3	Htt	Development
DMR14:81877101	14	81877101	300	1	7.46E-07	0	0	Poln	Transcription
DMR14:94354301	14	94354301	2600	1	1.28E-07	11	0.42		
DMR14:115001601	14	115001601	1900	1	8.20E-07	9	0.47	Acyp2	Signaling
DMR15:2140701	15	2140701	300	1	2.81E-07	2	0.66		
DMR15:2850801	15	2850801	300	1	1.20E-07	5	1.66	Kat6b	Epigenetic
DMR15:4499801	15	4499801	700	1	1.37E-07	7	1	LOC102554608	Unknown
DMR15:4588201	15	4588201	2200	2	3.35E-07	55	2.5	Kcnk5	Transport
DMR15:15103001	15	15103001	1300	1	3.56E-07	15	1.15		
DMR15:16436401	15	16436401	500	2	2.85E-09	5	1		
DMR15:18498101	15	18498101	3400	1	2.19E-07	30	0.88	Kctd6	Transport
DMR15:18600201	15	18600201	1800	1	8.52E-08	51	2.83	Pxk	Signaling
DMR15:21295801	15	21295801	1900	1	3.64E-08	27	1.42		
DMR15:33389701	15	33389701	1200	1	6.14E-07	18	1.5	Slc7a8	Transport
DMR15:34104801	15	34104801	1500	1	7.50E-07	51	3.4	AC116285.1	Unknown
DMR15:39222201	15	39222201	2500	1	9.85E-07	23	0.92		
DMR15:41418101	15	41418101	500	1	7.42E-07	8	1.6	AABR07018124.2	Unknown
DMR15:42222001	15	42222001	1800	1	7.19E-08	8	0.44		
DMR15:44000301	15	44000301	1200	1	7.59E-07	25	2.08	Ebf2	Immune

DMR15:44340001	15	44340001	2000	1	6.50E-07	20	1		
DMR15:53040901	15	53040901	1300	1	6.57E-07	11	0.84		
DMR15:54276501	15	54276501	300	2	3.11E-08	7	2.33		
DMR15:60077201	15	60077201	700	1	6.70E-07	4	0.57	Epsti1	Development
DMR15:74488201	15	74488201	200	1	9.60E-07	1	0.5		
DMR15:75291701	15	75291701	200	1	2.49E-07	1	0.5		
DMR15:82101201	15	82101201	100	1	1.20E-14	0	0	Dach1	Transcription
DMR15:83725201	15	83725201	1100	1	6.24E-08	13	1.18	Klf5	Transcription
DMR15:100724301	15	100724301	2200	1	5.82E-07	17	0.77		
DMR15:104206101	15	104206101	2100	1	1.87E-07	29	1.38	Dnajc3	Protein Binding
DMR15:105291001	15	105291001	4200	1	9.39E-10	54	1.28		
DMR15:105675901	15	105675901	800	3	9.85E-09	13	1.62	Mbnl2	Transcription
DMR15:106188901	15	106188901	1900	2	3.45E-11	33	1.73	AABR07019449.1	Unknown
DMR15:106514401	15	106514401	2400	2	1.79E-07	67	2.79	Farp1	Signaling
DMR15:108207701	15	108207701	1100	1	1.82E-07	20	1.81	Dock9	Cell Cycle
DMR15:108618501	15	108618501	200	1	1.22E-07	3	1.5	Clybl	Metabolism
DMR16:1778301	16	1778301	700	1	1.48E-07	14	2	Zmiz1	Metabolism
DMR16:6515701	16	6515701	900	1	9.35E-07	5	0.55		
DMR16:13028801	16	13028801	400	1	7.00E-07	0	0		
DMR16:15110201	16	15110201	2700	2	3.06E-09	40	1.48	LOC100913033	Unknown
DMR16:21735901	16	21735901	900	2	4.18E-09	4	0.44		
DMR16:21841301	16	21841301	2800	1	2.93E-07	23	0.82		
DMR16:22068001	16	22068001	4400	1	4.60E-07	36	0.81	RGD1563748	Unknown
DMR16:33102201	16	33102201	3300	1	1.52E-07	35	1.06		
DMR16:34729501	16	34729501	500	1	2.82E-08	4	0.8		
DMR16:71506901	16	71506901	900	1	4.55E-07	20	2.22		
DMR16:71546501	16	71546501	600	1	2.72E-07	14	2.33		
DMR16:75915601	16	75915601	1400	1	6.75E-07	14	1	Mcph1	DNA Repair
DMR16:81376801	16	81376801	1200	2	1.88E-08	20	1.66	Rasa3	Signaling
DMR16:85225001	16	85225001	1300	1	7.63E-07	13	1		
DMR16:88896301	16	88896301	3200	1	9.05E-08	18	0.56		
DMR17:3042201	17	3042201	5400	1	5.47E-09	41	0.75		
DMR17:8851001	17	8851001	3300	1	2.09E-07	68	2.06		
DMR17:12552501	17	12552501	300	1	1.03E-07	7	2.33		
DMR17:13573001	17	13573001	300	1	7.88E-07	3	1	Secisbp2	Binding Protein
DMR17:18991801	17	18991801	2300	1	4.07E-07	28	1.21		
DMR17:19307601	17	19307601	2200	1	3.13E-07	43	1.95	Atxn1	Transcription;DNA Repair
DMR17:19330101	17	19330101	2200	1	2.50E-07	44	2	Atxn1	Transcription;DNA Repair
DMR17:36320501	17	36320501	500	1	3.72E-08	0	0		
DMR17:39231401	17	39231401	2100	1	2.06E-07	26	1.23	Prl8a5	Hormone
DMR17:52869101	17	52869101	400	1	3.94E-07	6	1.5		
DMR17:57644901	17	57644901	2400	1	7.26E-07	20	0.83		
DMR17:64618401	17	64618401	300	1	3.12E-07	1	0.33		
DMR17:72782801	17	72782801	300	3	5.10E-08	4	1.33		
DMR17:76513301	17	76513301	1100	2	2.14E-10	16	1.45		
DMR17:81939901	17	81939901	300	1	9.69E-07	3	1	Cacnb2	Transport
DMR17:84934301	17	84934301	200	1	5.26E-07	10	5	Mllt10	Transcription
DMR18:4167301	18	4167301	300	1	2.18E-07	1	0.33	Osbpl1a	Binding Protein
DMR18:12699401	18	12699401	2700	1	6.35E-07	40	1.48		
DMR18:27783701	18	27783701	5700	1	1.72E-08	74	1.29		
DMR18:41906401	18	41906401	3200	1	5.35E-07	27	0.84	RGD1561627	Unknown
DMR18:42552901	18	42552901	300	1	2.32E-09	1	0.33		
DMR18:44322401	18	44322401	2900	1	5.94E-07	21	0.72		
DMR18:46848101	18	46848101	3300	1	4.24E-07	26	0.78		
DMR18:46956601	18	46956601	2000	1	3.81E-07	15	0.75		
DMR18:53164601	18	53164601	500	1	7.02E-07	17	3.4	Fbn2	Extracellular Matrix
DMR18:56703901	18	56703901	800	1	1.78E-07	8	1	Ppargc1b	Transcription
DMR18:60436501	18	60436501	600	2	3.40E-07	20	3.33	Nedd4l	Protease
DMR18:60654601	18	60654601	2000	2	7.27E-08	92	4.6	Nedd4l	Protease
DMR18:61811501	18	61811501	600	1	3.44E-07	5	0.83		
DMR18:71373601	18	71373601	2900	1	1.93E-07	32	1.1		
DMR18:79781201	18	79781201	2500	1	3.51E-07	63	2.52	Zfp516	Transcription
DMR18:83102801	18	83102801	200	2	9.48E-07	0	0		
DMR18:84276801	18	84276801	1100	1	1.65E-07	7	0.63		
DMR19:9915701	19	9915701	800	1	4.95E-07	7	0.87	Ccdc113	Unknown;Protease
DMR19:12859801	19	12859801	2600	1	2.38E-07	27	1.03	Large	Golgi
DMR19:17277601	19	17277601	3700	1	5.69E-08	64	1.72	Aktip	Proteolysis

DMR19:23544601	19	23544601	200	1	1.01E-08	2	1	Il15	Growth Factors & Cytokines
DMR19:25457701	19	25457701	900	1	2.61E-07	13	1.44		
DMR19:32512801	19	32512801	400	1	7.94E-07	16	4	Zfp827	Transcription
DMR19:40907601	19	40907601	400	1	2.08E-08	22	5.5	Mtss1l;Il34	Cytoskeleton;Growth Factors & Cytokines
DMR19:42216301	19	42216301	300	1	8.89E-08	2	0.66		
DMR19:44289101	19	44289101	1600	1	3.69E-07	9	0.56	AC117869.2	Unknown
DMR19:44950201	19	44950201	400	1	5.81E-07	2	0.5	Cntnap4	Signaling
DMR19:46793701	19	46793701	400	1	4.93E-07	18	4.5		
DMR19:46968301	19	46968301	800	1	6.13E-08	8	1		
DMR19:48180801	19	48180801	1600	1	9.76E-07	26	1.62		
DMR19:51829101	19	51829101	1900	1	1.04E-08	29	1.52	Cdh13	Extracellular Matrix
DMR19:52275101	19	52275101	300	1	8.07E-07	5	1.66	Kcng4	Transport
DMR19:55226901	19	55226901	400	1	3.78E-07	27	6.75	Zc3h18	Transcription
DMR19:58823401	19	58823401	200	1	6.51E-13	0	0	Kcnk1	Transport
DMR19:60395801	19	60395801	700	1	2.37E-07	16	2.28	Pard3	Cell Junction
DMR20:1727601	20	1727601	3400	1	9.42E-07	24	0.7	Olr1734	Unknown;Receptor
DMR20:2584601	20	2584601	2500	1	5.21E-07	17	0.68	Rn50_20_0026.4	Unknown
DMR20:3928401	20	3928401	1900	1	2.47E-07	40	2.1	RT1-DMa	Immune
DMR20:5190601	20	5190601	1400	1	6.59E-07	43	3.07	AC094348.1;Tnf	Unknown;Growth Factors & Cytokines
DMR20:5193201	20	5193201	300	1	4.62E-07	7	2.33	AC094348.1;Tnf	Unknown;Growth Factors & Cytokines
DMR20:7261601	20	7261601	6500	1	1.42E-07	106	1.63		
DMR20:9750901	20	9750901	2200	1	1.74E-07	51	2.31	Abcg1	Transport
DMR20:13606501	20	13606501	5600	1	4.19E-07	109	1.94	LOC103694884	Unknown
DMR20:13723101	20	13723101	400	1	1.71E-07	9	2.25	Mif	Growth Factors & Cytokines
DMR20:14171101	20	14171101	1100	1	6.26E-08	21	1.9	Upb1;AABR07044642.	Metabolism;Unknown
DMR20:14494701	20	14494701	1300	1	8.95E-08	31	2.38	Bcr	Signaling
DMR20:18017401	20	18017401	700	1	1.64E-07	3	0.42		
DMR20:32029601	20	32029601	300	1	6.59E-07	7	2.33	LOC100364027	Unknown
DMR20:32047601	20	32047601	400	1	2.72E-07	9	2.25	LOC100364027;Supv3	Unknown;Transcription
DMR20:36293901	20	36293901	400	1	8.26E-07	5	1.25		
DMR20:46843101	20	46843101	1400	3	1.31E-08	33	2.35	Armc2	Transcription
DMR20:48964701	20	48964701	900	1	8.23E-07	18	2	Aim1	Development
DMR20:50536901	20	50536901	5400	1	5.79E-07	169	3.12	Lin28b	Transcription
DMRX:12247201	X	12247201	500	1	6.86E-07	3	0.6		
DMRX:12411501	X	12411501	1700	1	2.56E-07	22	1.29		
DMRX:14287201	X	14287201	1800	1	8.31E-08	28	1.55	Rpgr	Signaling
DMRX:15731601	X	15731601	2100	1	4.97E-07	45	2.14	Cacna1f;Ccdc22	Transport;Unknown
DMRX:24092701	X	24092701	2800	1	9.07E-07	101	3.6		
DMRX:25780401	X	25780401	200	2	2.03E-09	0	0		
DMRX:61240401	X	61240401	3300	1	4.69E-07	27	0.81	Mageb18	Unknown
DMRX:63564801	X	63564801	27200	1	4.31E-07	227	0.83	Apoo	Extracellular Matrix
DMRX:64569901	X	64569901	900	1	7.64E-07	9	1		
DMRX:66271901	X	66271901	100	1	1.24E-09	1	1		
DMRX:81167501	X	81167501	100	1	2.35E-08	0	0		
DMRX:81181401	X	81181401	200	2	1.63E-14	0	0		
DMRX:90462701	X	90462701	200	1	4.87E-07	0	0		
DMRX:94662101	X	94662101	1100	1	8.16E-07	17	1.54		
DMRX:108075301	X	108075301	800	1	2.04E-07	3	0.37		
DMRX:124309601	X	124309601	100	1	4.32E-07	0	0		
DMRX:126684301	X	126684301	800	2	7.42E-09	70	8.75		
DMRX:130550301	X	130550301	300	1	4.11E-07	4	1.33	AABR07041449.1	Unknown
DMRX:141134201	X	141134201	200	2	6.78E-10	1	0.5		
DMRX:142171801	X	142171801	200	1	5.06E-07	0	0	Fgf13	Growth Factors & Cytokines
DMRX:150127601	X	150127601	2100	1	4.60E-07	9	0.42		
DMRY:1838201	Y	1838201	23200	3	7.48E-08	757	3.26		
