# Supplementary material for: Atrazine induced epigenetic transgenerational inheritance of disease, lean phenotype and sperm epimutation pathology biomarkers
Source: PLoS One. 2017 Sep 20;12(9):e0184306. doi: 10.1371/journal.pone.0184306 (PMC5606923; doi:10.1371/journal.pone.0184306)
Supplement: S4 Table — The DMR name, chromosome, start site, length (bp), number # significant windows, minimum p-value, CpG number, CpG % density, associated gene and gene category are presented. (PDF) [file pone.0184306.s010.pdf]

Supplemental Table S4
DMR F2 Generation DMR ($p<10^{-5}$) List

DMR Name	Chr	Start	Length	# Sig Win	Min p-value	CpG Num	CpG (%) Density	Associated Gene	Gene Category
DMR1:7189501	1	7189501	400	1	5.53E-06	2	0.5		
DMR1:20706501	1	20706501	300	1	1.91E-08	0	0		
DMR1:23278801	1	23278801	800	1	7.46E-06	12	1.5		
DMR1:23588701	1	23588701	200	1	7.72E-06	1	0.5	Eya4	Transcription
DMR1:26631101	1	26631101	1100	1	5.00E-06	4	0.36		
DMR1:29033301	1	29033301	900	1	9.28E-06	35	3.88		
DMR1:36466901	1	36466901	700	1	8.18E-06	6	0.85		
DMR1:38081701	1	38081701	700	1	2.27E-06	3	0.42		
DMR1:60130601	1	60130601	100	1	7.64E-06	0	0		
DMR1:68870401	1	68870401	200	1	9.17E-06	0	0		
DMR1:71337801	1	71337801	1000	1	4.98E-06	9	0.9	Zscan5b	Transcription
DMR1:77302501	1	77302501	1800	1	8.60E-06	6	0.33		
DMR1:90938001	1	90938001	1800	1	2.06E-07	28	1.55	AABR07002973.1;Sdhaf1	Unknown;Metabolism
DMR1:102818201	1	102818201	2700	1	8.41E-06	52	1.92	Hps5	Development
DMR1:105757501	1	105757501	1400	1	2.57E-06	12	0.85	Nell1	Development
DMR1:125693701	1	125693701	300	1	9.12E-06	1	0.33	Fam189a1	Unknown
DMR1:126981701	1	126981701	300	1	3.74E-07	1	0.33	Snrpa1;Vimp	Translation;Unknown
DMR1:133679801	1	133679801	300	1	3.07E-07	1	0.33		
DMR1:141076101	1	141076101	200	1	5.74E-06	4	2	AABR07004394.1;Abhd2	Unknown;Metabolism
DMR1:161668201	1	161668201	300	1	7.37E-06	5	1.66	Tenm4	Signaling
DMR1:171242101	1	171242101	600	1	9.17E-06	7	1.16		
DMR1:175890201	1	175890201	1100	1	1.84E-06	13	1.18	Eif4g2	Translation
DMR1:194849201	1	194849201	300	1	7.58E-07	7	2.33	AABR07005702.1	Unknown
DMR1:194915401	1	194915401	1400	2	2.57E-07	13	0.92	AABR07005709.8	Unknown
DMR1:206930501	1	206930501	1800	1	1.41E-06	35	1.94	Dock1	Signaling
DMR1:216964201	1	216964201	300	1	6.66E-06	0	0	Mrgpre	Receptor
DMR1:235241801	1	235241801	1300	1	6.48E-06	9	0.69	Gna14	Signaling
DMR1:242744701	1	242744701	1400	2	8.79E-08	12	0.85	Pgm5	Metabolism
DMR1:245961001	1	245961001	200	1	1.95E-06	1	0.5	Rfx3	Transcription
DMR1:248485201	1	248485201	2700	1	7.99E-06	23	0.85		
DMR1:250896801	1	250896801	400	1	1.14E-06	4	1	Sgms1	Metabolism
DMR1:253367201	1	253367201	300	1	3.93E-06	1	0.33		
DMR1:261309401	1	261309401	800	1	7.84E-06	2	0.25	Hoga1	Metabolism
DMR1:262799401	1	262799401	400	1	7.90E-06	4	1		
DMR1:264522801	1	264522801	200	1	5.59E-06	5	2.5	Pax2	Transcription
DMR1:266966501	1	266966501	400	1	4.90E-06	7	1.75	Neurl1	Protease
DMR1:275508301	1	275508301	200	1	2.48E-06	0	0		
DMR1:278449301	1	278449301	3200	3	8.43E-08	35	1.09		
DMR1:278644101	1	278644101	300	2	5.73E-07	5	1.66	Atrnl1	Signaling
DMR1:278964901	1	278964901	800	1	4.15E-06	6	0.75		
DMR1:279937301	1	279937301	200	1	8.41E-06	2	1	AABR07007083.1;Hspa12a	Unknown;Protein Binding
DMR2:2882101	2	2882101	200	1	1.63E-06	2	1	Arsk	Metabolism
DMR2:13371801	2	13371801	200	1	7.64E-06	1	0.5		
DMR2:15637201	2	15637201	300	1	1.67E-07	1	0.33		
DMR2:26788801	2	26788801	100	1	7.35E-07	2	2		
DMR2:28083401	2	28083401	2100	1	6.19E-06	33	1.57		
DMR2:29215701	2	29215701	200	1	8.99E-06	5	2.5		
DMR2:30135701	2	30135701	500	1	9.01E-06	9	1.8	Cartpt	Development
DMR2:35603601	2	35603601	2100	1	8.46E-06	22	1.04		
DMR2:40016501	2	40016501	200	1	9.11E-06	1	0.5	Depdc1b	Transcription
DMR2:51352001	2	51352001	400	1	5.42E-06	6	1.5		
DMR2:53388501	2	53388501	500	1	4.83E-06	1	0.2	Ghr	Receptor
DMR2:60036601	2	60036601	500	1	9.01E-07	4	0.8		
DMR2:62105901	2	62105901	300	1	6.42E-06	7	2.33		
DMR2:68393001	2	68393001	200	1	1.10E-07	0	0		
DMR2:83313001	2	83313001	200	1	3.04E-06	1	0.5		
DMR2:88978901	2	88978901	300	1	7.18E-06	8	2.66		
DMR2:94079701	2	94079701	700	1	3.48E-06	8	1.14		
DMR2:95065001	2	95065001	1400	1	2.35E-06	4	0.28	Mrps28	Translation
DMR2:102478501	2	102478501	2000	1	3.65E-06	13	0.65		
DMR2:123940801	2	123940801	900	1	5.67E-06	17	1.88		
DMR2:127226801	2	127226801	300	2	1.20E-07	2	0.66		
DMR2:156894801	2	156894801	2500	1	8.73E-06	10	0.4		
DMR2:172785601	2	172785601	200	1	8.28E-06	0	0		

DMR2:179101701	2	179101701	700	1	1.47E-06	2	0.28		
DMR2:180918201	2	180918201	1100	1	3.37E-07	11	1	AABR07011997.1;Tdo2	Unknown;Metabolism
DMR2:182241201	2	182241201	700	1	3.72E-06	9	1.28		
DMR2:185221401	2	185221401	1400	1	8.84E-06	22	1.57		
DMR2:197209301	2	197209301	2700	1	7.47E-06	154	5.7	AABR07012535.1	Unknown
DMR2:198175901	2	198175901	300	1	2.37E-06	4	1.33	Vps45	Transport
DMR2:214618201	2	214618201	400	1	8.04E-06	4	1		
DMR2:225187801	2	225187801	1400	1	1.09E-06	17	1.21		
DMR2:227722601	2	227722601	1200	1	5.17E-06	25	2.08	Prss12;AABR07013208.1	Proteolysis;Unknown
DMR2:240880201	2	240880201	3600	1	5.71E-06	49	1.36		
DMR2:246099301	2	246099301	100	1	3.16E-06	0	0		
DMR2:246140701	2	246140701	1200	1	8.30E-07	5	0.41		
DMR2:247759901	2	247759901	2200	1	6.40E-06	25	1.13		
DMR2:254359001	2	254359001	900	1	3.99E-06	15	1.66		
DMR2:255068801	2	255068801	400	1	3.82E-06	8	2	AABR07013762.2	Unknown
DMR2:259937201	2	259937201	1700	1	9.32E-06	22	1.29		
DMR2:262569501	2	262569501	600	1	1.48E-06	4	0.66		
DMR3:15122701	3	15122701	1600	1	8.06E-06	43	2.68	Ttll11	Protease
DMR3:15147401	3	15147401	1600	1	4.58E-06	30	1.87	Ttll11	Protease
DMR3:17086201	3	17086201	400	1	6.90E-06	2	0.5	AABR07051562.1	Unknown
DMR3:19888001	3	19888001	300	1	4.72E-08	0	0		
DMR3:21216401	3	21216401	300	1	8.59E-10	1	0.33	Olr427	Receptor
DMR3:21909701	3	21909701	1400	1	1.71E-07	9	0.64	Strbp	Translation
DMR3:51115101	3	51115101	1000	1	7.83E-06	21	2.1		
DMR3:52070001	3	52070001	5600	1	8.93E-06	69	1.23	Csrnp3	Unknown
DMR3:61841001	3	61841001	200	1	2.39E-06	2	1		
DMR3:62022301	3	62022301	200	1	4.28E-06	3	1.5		
DMR3:62960801	3	62960801	300	1	6.00E-07	0	0	Pde11a	Signaling
DMR3:67102601	3	67102601	1700	1	5.98E-08	12	0.7		
DMR3:70235601	3	70235601	900	1	8.88E-06	33	3.66		
DMR3:71723001	3	71723001	400	1	8.04E-07	6	1.5		
DMR3:74812201	3	74812201	400	2	3.63E-08	0	0	LOC100912540	Unknown
DMR3:82043001	3	82043001	300	1	1.49E-06	2	0.66		
DMR3:84523601	3	84523601	400	1	8.21E-06	2	0.5	AABR07052926.1	Unknown
DMR3:87624301	3	87624301	400	1	7.44E-06	2	0.5		
DMR3:88813601	3	88813601	300	1	3.95E-07	1	0.33		
DMR3:88851001	3	88851001	800	1	3.61E-06	16	2		
DMR3:91095101	3	91095101	400	1	3.04E-06	10	2.5	RGD1309730	Unknown
DMR3:96079201	3	96079201	300	1	2.84E-06	2	0.66		
DMR3:107598701	3	107598701	3600	2	4.33E-06	41	1.13	Meis2	Transcription
DMR3:110176401	3	110176401	300	1	8.46E-06	5	1.66	Eif2ak4	Signaling
DMR3:113339201	3	113339201	800	1	5.57E-06	14	1.75	Ppip5k1;Strc	Signaling;Extracellular Matrix
DMR3:117501101	3	117501101	500	1	9.23E-06	2	0.4	Slc12a1	Transport
DMR3:132491501	3	132491501	400	1	8.28E-06	4	1		
DMR3:140764501	3	140764501	1300	1	7.03E-07	21	1.61		
DMR3:147946401	3	147946401	2400	1	4.53E-06	35	1.45	Defb20	Unknown
DMR3:150713201	3	150713201	1300	1	7.70E-07	4	0.3	Itch	Proteolysis
DMR3:155391501	3	155391501	1600	1	6.44E-07	24	1.5		
DMR3:166306601	3	166306601	1600	1	9.92E-06	31	1.93		
DMR3:166832801	3	166832801	500	1	7.73E-08	11	2.2	AABR07054680.1	Unknown
DMR3:175194701	3	175194701	2300	1	4.12E-06	46	2	Cdh4	Extracellular Matrix
DMR4:12467001	4	12467001	400	1	3.13E-07	6	1.5	AABR07059258.1	Unknown
DMR4:45153601	4	45153601	700	1	2.98E-06	8	1.14	ST7	Miscellaneous
DMR4:45171501	4	45171501	500	1	4.27E-06	3	0.6	ST7	Miscellaneous
DMR4:71349701	4	71349701	200	1	2.42E-06	0	0	Sval2	Development
DMR4:71386901	4	71386901	200	1	4.90E-06	1	0.5	Sval1	Development
DMR4:73703301	4	73703301	100	1	7.64E-06	0	0		
DMR4:99286901	4	99286901	400	1	4.67E-06	4	1	Rmnd5a	Development
DMR4:100371901	4	100371901	200	1	1.04E-06	3	1.5	Sh2d6	Unknown
DMR4:111040201	4	111040201	200	1	1.90E-06	2	1	Lrrtm4	Receptor
DMR4:115173701	4	115173701	200	1	6.48E-06	2	1	Dguok	Signaling
DMR4:120889501	4	120889501	400	1	1.31E-07	4	1	AC117122.1	Unknown
DMR4:123506401	4	123506401	1600	1	9.17E-06	17	1.06	Slc41a3;Aldh1l1	Transport;Metabolism
DMR4:124371501	4	124371501	800	1	5.59E-06	5	0.62		
DMR4:132606501	4	132606501	900	1	9.63E-06	56	6.22		
DMR4:153704401	4	153704401	100	1	9.64E-06	0	0		
DMR4:168507501	4	168507501	1300	1	3.35E-06	11	0.84	Dusp16	Signaling
DMR4:177263301	4	177263301	1100	1	7.63E-07	13	1.18	C2cd5	Signaling

DMR4:177489301	4	177489301	4000	1	9.12E-06	57	1.42		
DMR5:170301	5	170301	1400	1	2.24E-07	9	0.64	Tipinl1	Development
DMR5:4628801	5	4628801	1200	1	2.05E-06	15	1.25		
DMR5:17062301	5	17062301	400	1	9.42E-06	4	1	Penk;AABR07047044.2;AA	Signaling;Unknown
DMR5:22125601	5	22125601	300	1	4.46E-07	17	5.66		
DMR5:25635201	5	25635201	2000	1	8.78E-06	38	1.9		
DMR5:32049701	5	32049701	300	1	2.05E-06	1	0.33		
DMR5:34787801	5	34787801	1100	1	5.98E-06	3	0.27	Nkain3	Transport
DMR5:42760301	5	42760301	500	1	7.17E-08	3	0.6		
DMR5:57222201	5	57222201	700	1	6.48E-06	11	1.57		
DMR5:59121801	5	59121801	400	1	7.26E-06	1	0.25	AC121204.4;Npr2	Unknown;Receptor
DMR5:59590201	5	59590201	1300	1	2.07E-06	17	1.3		
DMR5:68987401	5	68987401	500	1	1.03E-08	3	0.6		
DMR5:71198101	5	71198101	400	2	4.19E-07	4	1		
DMR5:74410701	5	74410701	300	1	6.57E-06	0	0		
DMR5:78777901	5	78777901	200	1	8.41E-06	0	0		
DMR5:121137501	5	121137501	300	1	8.29E-06	1	0.33		
DMR5:124851701	5	124851701	400	1	1.54E-06	4	1		
DMR5:126906101	5	126906101	200	1	6.09E-07	2	1	Dio1	Metabolism
DMR5:129675301	5	129675301	300	1	2.84E-06	2	0.66	Faf1	Apoptosis
DMR5:142355001	5	142355001	1000	1	3.21E-06	11	1.1	LOC100909856	Unknown
DMR5:148401601	5	148401601	400	1	3.04E-06	3	0.75	Tinagl1	Signaling
DMR5:166731001	5	166731001	1200	1	7.46E-06	20	1.66	Slc25a33	Transport
DMR5:167108201	5	167108201	1700	1	3.04E-06	72	4.23	Gpr157	Receptor
DMR5:169813501	5	169813501	400	1	3.09E-06	6	1.5		
DMR6:7285301	6	7285301	2400	1	5.23E-07	53	2.2		
DMR6:11894801	6	11894801	1800	1	1.60E-06	19	1.05		
DMR6:27034301	6	27034301	500	1	3.14E-06	7	1.4	AABR07063286.1	Unknown
DMR6:33353501	6	33353501	1400	1	1.18E-06	9	0.64		
DMR6:34058701	6	34058701	100	1	5.95E-06	0	0	Laptm4a	Transport
DMR6:44430901	6	44430901	1000	1	1.47E-06	11	1.1		
DMR6:48957201	6	48957201	200	1	5.00E-06	0	0	Pxdn	Metabolism
DMR6:50429301	6	50429301	300	1	7.56E-07	5	1.66		
DMR6:51072901	6	51072901	1400	1	5.48E-06	10	0.71	Cog5	Golgi
DMR6:61996301	6	61996301	300	1	5.44E-06	4	1.33		
DMR6:70397601	6	70397601	700	1	3.63E-08	3	0.42		
DMR6:85128301	6	85128301	200	1	9.01E-06	3	1.5		
DMR6:87393701	6	87393701	300	1	1.91E-07	2	0.66	AABR07064622.2	Unknown
DMR6:110514801	6	110514801	200	1	5.71E-06	2	1	AABR07065115.1	Unknown
DMR6:117957901	6	117957901	300	1	1.95E-06	0	0	AABR07065265.1	Unknown
DMR6:130832201	6	130832201	1600	1	5.98E-06	14	0.87		
DMR6:134514801	6	134514801	1500	1	5.59E-06	27	1.8		
DMR6:136876201	6	136876201	2600	1	3.77E-06	57	2.19		
DMR6:138935101	6	138935101	300	1	4.46E-07	2	0.66	LOC100359993	Unknown
DMR6:142894801	6	142894801	1200	1	5.62E-06	5	0.41	NA	NA
DMR7:1950201	7	1950201	300	1	1.23E-06	3	1		
DMR7:2147501	7	2147501	2300	1	5.91E-06	7	0.3		
DMR7:5158801	7	5158801	2500	1	1.63E-06	22	0.88	Olr894	Receptor
DMR7:13637601	7	13637601	1400	1	3.97E-06	10	0.71	Olr1084	Receptor
DMR7:16467401	7	16467401	200	2	4.12E-08	0	0	Olr1055	Receptor
DMR7:17058701	7	17058701	1400	1	5.48E-06	13	0.92		
DMR7:22026001	7	22026001	800	1	9.31E-06	4	0.5		
DMR7:22360601	7	22360601	2600	1	4.78E-06	14	0.53		
DMR7:25239501	7	25239501	200	1	2.81E-07	2	1		
DMR7:34522301	7	34522301	900	1	8.76E-06	8	0.88		
DMR7:35002301	7	35002301	400	1	5.01E-06	5	1.25	Fgd6	Signaling
DMR7:37748801	7	37748801	700	1	5.98E-08	3	0.42		
DMR7:40061201	7	40061201	3500	1	4.94E-06	29	0.82		
DMR7:40976201	7	40976201	600	1	2.57E-06	12	2		
DMR7:41390501	7	41390501	1000	1	9.37E-06	11	1.1	Poc1b	Unknown
DMR7:50521901	7	50521901	200	1	8.17E-07	0	0	Syt1	Transport
DMR7:53073301	7	53073301	1100	1	9.61E-10	7	0.63		
DMR7:54391401	7	54391401	800	1	6.66E-06	23	2.87		
DMR7:61271901	7	61271901	900	1	9.47E-07	7	0.77		
DMR7:61301101	7	61301101	400	2	3.38E-10	2	0.5		
DMR7:61354101	7	61354101	200	1	1.54E-06	0	0		
DMR7:65165201	7	65165201	200	1	1.29E-06	1	0.5	Hmga2	Transcription
DMR7:69769801	7	69769801	300	1	9.49E-06	4	1.33		

DMR7:80695501	7	80695501	500	1	8.31E-06	7	1.4	Oxr1	Development
DMR7:80750101	7	80750101	200	1	9.40E-06	1	0.5	Oxr1	Development
DMR7:101971201	7	101971201	300	1	1.32E-07	1	0.33		
DMR7:115750901	7	115750901	200	1	7.46E-06	0	0		
DMR7:117193701	7	117193701	400	1	8.76E-06	5	1.25	LOC680875	Unknown
DMR7:118384401	7	118384401	300	1	1.09E-06	5	1.66	Rbfox2	Epigenetic
DMR7:127202901	7	127202901	200	1	4.28E-06	3	1.5	Tbc1d22a	Signaling
DMR7:140457101	7	140457101	300	2	2.59E-08	1	0.33	Wnt10b;Wnt1	Signaling
DMR7:141568501	7	141568501	2200	1	2.57E-06	28	1.27	AABR07058884.2	Unknown
DMR7:144589801	7	144589801	300	1	5.57E-06	12	4	Hoxc9;Mir196a;Hoxc10	Transcription;Epigenetic
DMR8:12319801	8	12319801	1000	1	5.95E-06	10	1	Mtmr2	Signaling
DMR8:13593701	8	13593701	1700	1	6.84E-06	26	1.52	Panx1	Cell Junction
DMR8:18428701	8	18428701	300	1	6.35E-07	2	0.66	Muc16;Mbd3l1	Cytoskeleton;Epigenetic
DMR8:21275601	8	21275601	700	1	6.66E-06	7	1	LOC100361194	Receptor
DMR8:34135401	8	34135401	1200	1	1.07E-06	8	0.66		
DMR8:37139801	8	37139801	300	1	4.78E-07	0	0		
DMR8:38063101	8	38063101	200	1	4.28E-06	1	0.5		
DMR8:45760601	8	45760601	300	1	5.74E-06	4	1.33	Mir3596a	Epigenetic
DMR8:52956201	8	52956201	1000	1	5.76E-06	13	1.3		
DMR8:53470101	8	53470101	400	2	4.40E-06	3	0.75		
DMR8:77248301	8	77248301	2300	2	3.16E-07	27	1.17		
DMR8:77551501	8	77551501	2800	1	3.04E-06	31	1.1	Aqp9	Transport
DMR8:83510701	8	83510701	1300	1	6.21E-06	6	0.46		
DMR8:84508301	8	84508301	1500	1	2.25E-06	18	1.2	Mlip	Unknown
DMR8:98877401	8	98877401	300	1	3.01E-06	2	0.66		
DMR8:111944801	8	111944801	4000	1	4.15E-06	59	1.47	5S_rRNA;Bfsp2	Translation;Cytoskeleton
DMR8:126807301	8	126807301	1500	1	5.65E-06	8	0.53		
DMR8:130555901	8	130555901	2300	1	9.91E-06	50	2.17	Cyp8b1	Metabolism
DMR9:1402101	9	1402101	1100	1	2.52E-07	8	0.72	Tbc1d5	Signaling
DMR9:4026101	9	4026101	300	1	4.20E-06	1	0.33		
DMR9:4157201	9	4157201	1200	1	5.03E-06	7	0.58	Sult1c2a;Sult1c2	Metabolism
DMR9:15286101	9	15286101	1200	1	8.21E-06	15	1.25		
DMR9:21269301	9	21269301	200	1	2.55E-07	0	0	Ptchd4	Unknown
DMR9:21926101	9	21926101	400	1	9.01E-06	3	0.75		
DMR9:28757401	9	28757401	1800	1	2.02E-06	15	0.83	Rims1	Signaling
DMR9:36859501	9	36859501	300	1	9.01E-06	3	1		
DMR9:50192201	9	50192201	300	1	6.80E-06	4	1.33	AABR07067507.2	Unknown
DMR9:62274501	9	62274501	200	1	8.28E-06	6	3		
DMR9:62614801	9	62614801	1100	1	6.37E-08	4	0.36		
DMR9:65751801	9	65751801	200	1	5.76E-06	2	1	Trak2	Transport
DMR9:74188701	9	74188701	1300	1	1.62E-06	13	1	Cps1	Unknown
DMR9:74854701	9	74854701	400	1	7.46E-06	5	1.25		
DMR9:79907001	9	79907001	400	1	3.99E-06	7	1.75	4-Mar	Metabolism
DMR9:84170201	9	84170201	200	2	9.90E-07	0	0		
DMR9:85401801	9	85401801	800	2	4.87E-07	2	0.25	Ap1s3	Transport
DMR9:105116701	9	105116701	2000	1	5.65E-06	37	1.85		
DMR9:106262201	9	106262201	200	1	8.76E-06	2	1		
DMR9:110818701	9	110818701	300	1	9.01E-06	6	2	Fbxl17	Proteolysis
DMR9:111161501	9	111161501	2400	1	8.16E-06	41	1.7	Pam	Metabolism
DMR10:3742101	10	3742101	800	1	4.67E-06	24	3	Cpped1	Signaling
DMR10:18536301	10	18536301	200	1	7.64E-06	2	1		
DMR10:19833701	10	19833701	3600	1	8.76E-06	56	1.55		
DMR10:43734701	10	43734701	300	1	8.01E-06	3	1	RGD1308564;Zfp692	Unknown;Transcription
DMR10:47223201	10	47223201	2400	1	6.48E-06	28	1.16		
DMR10:51927801	10	51927801	1200	1	6.54E-06	11	0.91		
DMR10:55962001	10	55962001	2400	1	4.40E-06	33	1.37	Chd3	Epigenetic
DMR10:61205401	10	61205401	3700	1	2.46E-06	31	0.83	Rap1gap2	Signaling
DMR10:63890301	10	63890301	600	1	2.74E-06	7	1.16	Ywhae	Metabolism
DMR10:80522201	10	80522201	800	1	9.01E-06	6	0.75		
DMR10:85216101	10	85216101	1000	1	3.04E-07	8	0.8	Npepps	Protease
DMR10:96641001	10	96641001	200	1	8.41E-06	3	1.5	Apoh	Binding Protein
DMR10:98695901	10	98695901	1100	1	2.52E-08	14	1.27	Map2k6	Signaling
DMR10:106578601	10	106578601	900	1	5.67E-06	18	2	LOC102547817	Unknown
DMR10:109127201	10	109127201	100	1	5.67E-06	2	2	Baiap2	Receptor
DMR11:13375001	11	13375001	200	1	5.65E-06	0	0		
DMR11:24558001	11	24558001	400	1	9.01E-06	2	0.5	App	Signaling
DMR11:30323201	11	30323201	200	1	5.97E-06	1	0.5		
DMR11:37632401	11	37632401	900	1	9.14E-07	7	0.77		

DMR11:38015101	11	38015101	1500	1	9.49E-06	22	1.46		
DMR11:42398501	11	42398501	200	1	4.67E-06	2	1	Epha6	Signaling
DMR11:47427101	11	47427101	200	1	7.95E-06	0	0		
DMR11:58502601	11	58502601	300	1	6.57E-06	1	0.33		
DMR11:66791701	11	66791701	900	1	7.15E-06	16	1.77	Golgb1	Golgi
DMR11:68720601	11	68720601	1200	1	8.21E-06	7	0.58	Adcy5	Signaling
DMR12:8920001	12	8920001	2000	1	1.56E-10	46	2.3		
DMR12:13325101	12	13325101	300	1	5.42E-06	6	2	AC126572.1;Zfp853	Unknown;Transcription
DMR12:14969701	12	14969701	300	1	2.54E-06	4	1.33	Sdk1	Unknown
DMR12:15350801	12	15350801	200	1	4.90E-06	1	0.5	Rn50_12_0174.1	Unknown
DMR12:20081501	12	20081501	1400	1	5.17E-06	7	0.5		
DMR12:22019601	12	22019601	300	1	4.15E-06	4	1.33	Nyap1;LOC102550456	Signaling;Transcription
DMR12:24463901	12	24463901	200	1	4.25E-06	4	2	Fzd9	Signaling
DMR12:26868601	12	26868601	800	1	3.46E-06	9	1.12		
DMR12:41493601	12	41493601	200	1	1.05E-09	3	1.5	Ddx54;Iqcd;Rita1	Transcription;Unknown;Cytoskelet[illegible]
DMR12:47613501	12	47613501	700	1	4.86E-06	14	2	Git2	Signaling
DMR12:49009001	12	49009001	600	1	4.61E-06	5	0.83		
DMR13:7675301	13	7675301	1200	1	3.22E-06	20	1.66		
DMR13:19510401	13	19510401	300	1	1.03E-06	1	0.33		
DMR13:26868001	13	26868001	500	1	9.01E-06	4	0.8		
DMR13:35967501	13	35967501	1100	1	3.24E-06	11	1	Cfap221	Development
DMR13:44597201	13	44597201	1300	1	8.15E-06	11	0.84		
DMR13:46539501	13	46539501	200	1	4.99E-06	0	0	Thsd7b	Extracellular Matrix
DMR13:47235401	13	47235401	1300	1	2.96E-06	9	0.69		
DMR13:51569901	13	51569901	800	1	4.15E-06	6	0.75	Syt2	Transport
DMR13:53582501	13	53582501	200	1	3.48E-06	6	3		
DMR13:58077501	13	58077501	300	1	9.32E-06	1	0.33		
DMR13:71376001	13	71376001	500	1	7.61E-11	7	1.4		
DMR13:71955701	13	71955701	400	1	3.62E-06	1	0.25	Cacna1e	Transport
DMR13:85794501	13	85794501	1300	1	6.19E-06	15	1.15		
DMR13:87892301	13	87892301	500	1	1.71E-07	2	0.4		
DMR13:95331301	13	95331301	400	1	1.95E-07	4	1	Akt3	Signaling
DMR13:104767201	13	104767201	300	1	1.69E-06	6	2		
DMR13:106087101	13	106087101	300	1	8.67E-06	4	1.33		
DMR13:107435901	13	107435901	1600	1	7.46E-06	30	1.87	Kctd3;Ush2a	Transport;Extracellular Matrix
DMR13:112074101	13	112074101	300	2	2.21E-06	2	0.66	Camk1g;Lamb3	Signaling;Extracellular Matrix
DMR14:3747501	14	3747501	300	1	8.91E-06	2	0.66		
DMR14:4753001	14	4753001	1200	1	8.46E-06	20	1.66		
DMR14:11232701	14	11232701	400	1	4.37E-07	3	0.75		
DMR14:11891701	14	11891701	1400	1	4.15E-06	18	1.28		
DMR14:16944401	14	16944401	400	1	6.84E-06	1	0.25	Ccdc158	Unknown
DMR14:21009201	14	21009201	2400	1	1.03E-06	36	1.5		
DMR14:30356701	14	30356701	200	1	7.88E-08	1	0.5		
DMR14:31841101	14	31841101	1700	1	5.65E-06	14	0.82		
DMR14:36929001	14	36929001	1400	1	3.79E-06	8	0.57		
DMR14:37783301	14	37783301	2000	1	7.83E-09	30	1.5		
DMR14:39373501	14	39373501	1300	1	9.17E-06	25	1.92	Cox7b2	Metabolism
DMR14:43054701	14	43054701	900	1	5.65E-06	12	1.33	Limch1	Cytoskeleton
DMR14:44668601	14	44668601	500	1	6.63E-06	16	3.2	Rfc1	Transcription
DMR14:45715601	14	45715601	300	1	1.70E-06	4	1.33	Tbc1d1	Signaling
DMR14:46672801	14	46672801	500	1	8.54E-06	13	2.6	AABR07015087.1	Unknown
DMR14:49884401	14	49884401	100	1	5.67E-06	3	3		
DMR14:59828001	14	59828001	1900	1	5.33E-06	33	1.73		
DMR14:60537801	14	60537801	1600	1	6.19E-06	39	2.43	Zcchc4	Transcription
DMR14:70923501	14	70923501	100	1	4.21E-06	3	3	Ldb2	Transcription
DMR14:78946601	14	78946601	400	1	1.45E-06	4	1	Mrfap1;Man2b2	Unknown;Metabolism
DMR14:79721201	14	79721201	400	1	5.00E-06	2	0.5	Sorcs2;Psapl1	Receptor;Unknown
DMR14:81772001	14	81772001	1400	1	7.64E-06	17	1.21	Zfyve28	Transcription
DMR14:84637301	14	84637301	300	1	1.00E-06	1	0.33	Hormad2	Transcription
DMR14:89420201	14	89420201	400	1	5.33E-06	0	0	Abca13	Transport
DMR14:96163101	14	96163101	400	1	9.17E-06	8	2		
DMR14:96744501	14	96744501	900	1	2.63E-06	7	0.77		
DMR14:101034701	14	101034701	3300	1	6.19E-07	19	0.57		
DMR15:10042301	15	10042301	300	1	6.62E-06	0	0		
DMR15:21923101	15	21923101	300	1	8.27E-06	3	1		
DMR15:23225401	15	23225401	800	1	5.53E-06	3	0.37		
DMR15:31425901	15	31425901	200	1	3.21E-06	0	0	AABR07017824.2	Unknown
DMR15:38024401	15	38024401	1900	1	6.69E-06	15	0.78		

DMR15:43537601	15	43537601	200	1	6.62E-06	3	1.5	AABR07018155.1;Dpysl2	Unknown;Metabolism
DMR15:54356401	15	54356401	1500	1	9.34E-06	7	0.46		
DMR15:56299501	15	56299501	1000	1	1.40E-06	44	4.4		
DMR15:56625401	15	56625401	800	1	4.08E-06	18	2.25		
DMR15:57179501	15	57179501	600	1	1.55E-06	11	1.83	Lrrc63;Esco2-ps2	Miscellaneous;Unknown
DMR15:67141601	15	67141601	200	1	4.15E-06	5	2.5		
DMR15:74455801	15	74455801	400	1	5.42E-06	3	0.75		
DMR15:78506001	15	78506001	400	1	9.48E-07	2	0.5		
DMR15:80623401	15	80623401	300	1	6.35E-07	2	0.66	Klhl1	Cytoskeleton
DMR15:89480601	15	89480601	200	1	5.19E-06	1	0.5		
DMR15:95503601	15	95503601	300	1	2.30E-07	4	1.33	Slitrk6	Receptor
DMR15:102238101	15	102238101	300	1	1.24E-07	6	2	Gpc6	Extracellular Matrix
DMR15:106389901	15	106389901	600	1	2.48E-08	12	2		
DMR15:106514301	15	106514301	2700	1	6.18E-07	73	2.7	Farp1	Signaling
DMR16:6836401	16	6836401	700	1	2.57E-06	14	2	Sfmbt1	Transcription
DMR16:15103401	16	15103401	1800	1	8.74E-06	7	0.38	LOC100913033	Unknown
DMR16:19844301	16	19844301	300	1	2.93E-06	7	2.33		
DMR16:22017901	16	22017901	3600	1	1.55E-07	48	1.33	RGD1563748	Unknown
DMR16:45907401	16	45907401	100	1	1.61E-06	2	2		
DMR16:47258001	16	47258001	400	1	9.01E-06	4	1		
DMR16:57747301	16	57747301	1100	1	4.50E-06	12	1.09		
DMR16:69248501	16	69248501	1100	1	3.51E-06	14	1.27	Zfp703	Transcription
DMR16:72208301	16	72208301	300	1	2.17E-06	0	0	Adam18;Ido1	Protease;Signaling
DMR16:74587701	16	74587701	200	1	3.77E-06	3	1.5	Tpte2;AABR07026382.1	Signaling;Unknown
DMR16:79920001	16	79920001	1200	1	7.46E-06	5	0.41	Dlgap2	Receptor
DMR16:82450301	16	82450301	300	1	1.58E-06	5	1.66		
DMR16:83881601	16	83881601	1400	1	6.23E-07	19	1.35		
DMR17:1688901	17	1688901	400	1	9.87E-06	2	0.5	Habp4	Miscellaneous
DMR17:6455801	17	6455801	2000	1	1.51E-09	33	1.65	Slc28a3	Transport
DMR17:13451401	17	13451401	2100	1	5.33E-06	39	1.85		
DMR17:13702701	17	13702701	200	1	5.33E-06	1	0.5	Shc3	Signaling
DMR17:15830201	17	15830201	400	1	2.51E-06	5	1.25	Susd3	Development
DMR17:19790301	17	19790301	700	1	4.51E-07	6	0.85	AABR07027213.1	Unknown
DMR17:22653201	17	22653201	1200	1	2.60E-06	17	1.41	LOC100362172;Adtrp	Unknown;Development
DMR17:33594901	17	33594901	300	1	5.94E-07	3	1	Gmds	Metabolism
DMR17:48948201	17	48948201	300	1	9.92E-06	2	0.66		
DMR17:53447201	17	53447201	200	1	9.57E-06	7	3.5	Hecw1	Protease
DMR17:58197501	17	58197501	200	1	2.23E-06	2	1	Adarb2	Metabolism
DMR17:61783501	17	61783501	1300	1	1.90E-06	8	0.61		
DMR18:11772001	18	11772001	400	1	3.20E-06	16	4	Dsc3;AABR07031381.1	Unknown
DMR18:14522201	18	14522201	900	1	7.36E-07	6	0.66	Dtna	Cytoskeleton
DMR18:35948501	18	35948501	300	1	9.30E-06	0	0		
DMR18:44450501	18	44450501	200	1	7.70E-07	0	0		
DMR18:56771401	18	56771401	1100	1	1.69E-06	3	0.27		
DMR18:59177201	18	59177201	600	1	4.17E-06	9	1.5	Wdr7	Unknown
DMR18:62839401	18	62839401	1900	1	6.19E-06	26	1.36	Gnal	Signaling
DMR18:65343101	18	65343101	2100	1	4.58E-08	39	1.85	Tcf4	Transcription
DMR18:70621201	18	70621201	1300	2	3.03E-06	21	1.61	Myo5b	Cytoskeleton
DMR19:190001	19	190001	1400	1	1.48E-06	2	0.14		
DMR19:193801	19	193801	1200	1	6.87E-06	7	0.58		
DMR19:313001	19	313001	1700	1	4.40E-07	9	0.52	AABR07042611.1	Unknown
DMR19:14729801	19	14729801	1900	1	3.84E-06	38	2		
DMR19:23960801	19	23960801	800	1	5.92E-07	3	0.37		
DMR19:26483401	19	26483401	2100	2	1.23E-06	30	1.42		
DMR19:28171401	19	28171401	900	1	8.82E-06	19	2.11		
DMR19:28649001	19	28649001	1600	1	5.43E-07	6	0.37	AABR07043449.1	Unknown
DMR19:28659601	19	28659601	1800	1	4.05E-07	37	2.05	AABR07043453.1	Unknown
DMR19:33326001	19	33326001	300	1	7.53E-07	2	0.66	Ttc29	Development
DMR19:35941901	19	35941901	400	1	7.13E-06	2	0.5		
DMR19:41177601	19	41177601	2300	1	1.86E-09	26	1.13	Hydin	Unknown
DMR19:42523001	19	42523001	300	2	5.02E-06	2	0.66	AABR07043825.1	Unknown
DMR19:50508601	19	50508601	300	1	1.32E-06	4	1.33		
DMR19:54171701	19	54171701	600	1	3.25E-06	8	1.33	5S_rRNA;Gins2;AC118833.	Translation;Transcription;Unknowr
DMR19:54997301	19	54997301	600	1	3.81E-06	19	3.16		
DMR20:2123901	20	2123901	1100	1	2.44E-06	12	1.09	LOC102554813	Unknown
DMR20:2793401	20	2793401	600	5	3.30E-11	4	0.66	Rn60_20_0028.1	Unknown
DMR20:2849401	20	2849401	600	3	2.85E-11	5	0.83	Btnl8	Immune
DMR20:2971901	20	2971901	200	1	6.29E-06	1	0.5	AABR07044346.1	Unknown

DMR20:3163601	20	3163601	1000	1	8.18E-06	8	0.8	AABR07044362.1;Rn50_2(	Unknown;Immune
DMR20:4021601	20	4021601	500	2	1.63E-09	4	0.8	RT1-DOb	Immune
DMR20:4026001	20	4026001	1200	1	1.65E-06	14	1.16	RT1-DOb	Immune
DMR20:4047001	20	4047001	5700	2	1.34E-08	68	1.19	RT1-Bb	Immune
DMR20:4055901	20	4055901	600	2	2.09E-06	9	1.5	RT1-Bb;RT1-Ba	Immune
DMR20:4058301	20	4058301	700	4	2.67E-15	9	1.28	RT1-Bb;RT1-Ba	Immune
DMR20:4064101	20	4064101	800	3	1.19E-10	10	1.25	RT1-Ba	Immune
DMR20:4281501	20	4281501	200	1	8.75E-06	2	1	Cyp21a1-ps;RGD1624210	Unknown;Immune
DMR20:4897901	20	4897901	900	1	8.41E-06	4	0.44	AABR07044408.1;RT1-CE4	Unknown;Immune
DMR20:5205301	20	5205301	2900	2	7.08E-08	45	1.55	AABR07044414.1	Unknown
DMR20:15103801	20	15103801	3600	1	7.83E-06	42	1.16	Pcdh15	Extracellular Matrix
DMR20:16182101	20	16182101	1500	2	5.59E-06	42	2.8		
DMR20:19346301	20	19346301	2000	2	1.02E-08	30	1.5	Phyhipl	Unknown
DMR20:20976201	20	20976201	200	1	1.02E-07	4	2	AABR07044799.1	Unknown
DMR20:32557801	20	32557801	200	1	8.15E-06	1	0.5	Kpna5	Binding Protein
DMR20:36670301	20	36670301	2100	1	3.27E-08	33	1.57		
DMR20:42093101	20	42093101	100	1	2.44E-06	0	0		
DMR20:45170801	20	45170801	300	1	9.10E-07	2	0.66	Slc16a10	Transport
DMR20:48581601	20	48581601	400	1	5.04E-06	6	1.5	Mettl24;Cdc40	Epigenetic;Cell Cycle
DMR20:50310601	20	50310601	300	1	1.42E-06	2	0.66		
DMRX:17848401	X	17848401	2500	1	9.32E-06	23	0.92		
DMRX:21137501	X	21137501	400	1	3.35E-06	0	0	Phf8	Cell Cycle
DMRX:50759801	X	50759801	300	1	3.04E-06	2	0.66		
DMRX:74784901	X	74784901	300	1	2.44E-06	4	1.33		
DMRX:92626401	X	92626401	300	1	5.32E-07	3	1	Pcdh11x	Extracellular Matrix
DMRX:100671401	X	100671401	300	1	1.05E-07	6	2		
DMRX:118227501	X	118227501	200	1	7.46E-06	2	1	Htr2c	Receptor
