# Supplementary material for: Atrazine induced epigenetic transgenerational inheritance of disease, lean phenotype and sperm epimutation pathology biomarkers
Source: PLoS One. 2017 Sep 20;12(9):e0184306. doi: 10.1371/journal.pone.0184306 (PMC5606923; doi:10.1371/journal.pone.0184306)
Supplement: S5 Table — The DMR name, chromosome, start site, length (bp), number # significant windows, minimum p-value, CpG number, CpG % density, associated gene and gene category are presented. (PDF) [file pone.0184306.s011.pdf]

Supplemental Table S5
DMR F3 Generation DMR ($p<10^{-9}$) List

DMR Name	Chr	Start	Length	# Sig Win	Min p-value	CpG Num	CpG (%) Density	Associated Gene	Gene Category
DMR1:2426701	1	2426701	4600	2	6.10E-12	45	0.97	Ust	Metabolism
DMR1:6287201	1	6287201	8600	1	1.65E-13	75	0.87	AABR07000248.1	Unknown
DMR1:14251201	1	14251201	2200	2	7.61E-12	18	0.81		
DMR1:15910601	1	15910601	8600	1	2.79E-11	105	1.22	Pde7b	Signaling
DMR1:16209201	1	16209201	800	2	1.55E-12	0	0	Pde7b	Signaling
DMR1:16962601	1	16962601	4200	2	7.91E-11	13	0.3	AABR07000533.2	Unknown
DMR1:18708201	1	18708201	2000	1	3.05E-10	16	0.8	Lama2	Extracellular Matrix
DMR1:19030801	1	19030801	800	1	8.32E-10	6	0.75	Lama2	Extracellular Matrix
DMR1:20229301	1	20229301	6600	1	1.32E-14	42	0.63	AABR07000629.2	Unknown
DMR1:21710101	1	21710101	2800	1	8.77E-12	22	0.78	AC127189.1	Unknown
DMR1:22666401	1	22666401	2800	1	2.91E-10	19	0.67	Vnn3	Metabolism
DMR1:25013301	1	25013301	5000	1	3.16E-11	59	1.18		
DMR1:26356301	1	26356301	3000	1	9.04E-10	14	0.46		
DMR1:29821301	1	29821301	1600	1	7.70E-10	13	0.81		
DMR1:33499601	1	33499601	4200	1	3.39E-10	31	0.73		
DMR1:38337601	1	38337601	1600	1	5.52E-11	20	1.25		
DMR1:41983401	1	41983401	1100	1	1.71E-10	6	0.54	Syne1	Development
DMR1:44642601	1	44642601	600	2	1.54E-13	2	0.33		
DMR1:45370501	1	45370501	2100	1	6.01E-10	25	1.19		
DMR1:46255201	1	46255201	5100	1	1.37E-10	69	1.35		
DMR1:48560301	1	48560301	2300	1	7.59E-11	14	0.6	Plg;AABR07001512.1	Protease;Unknown
DMR1:52111001	1	52111001	6000	2	6.78E-11	59	0.98		
DMR1:58674001	1	58674001	2900	1	2.39E-10	18	0.62		
DMR1:64600501	1	64600501	500	1	9.41E-10	2	0.4		
DMR1:72950801	1	72950801	2000	1	2.33E-10	14	0.7	Rdh13;Eps8l1	Metabolism;Receptor
DMR1:75746501	1	75746501	2700	1	4.23E-10	8	0.29	Bsph1	Development
DMR1:78699001	1	78699001	2200	1	8.10E-10	16	0.72	Slc1a5	Transport
DMR1:82004201	1	82004201	2200	1	2.52E-10	89	4.04	Pou2f2	Transcription
DMR1:89434101	1	89434101	3200	2	1.55E-10	19	0.59		
DMR1:93812301	1	93812301	3200	1	7.89E-14	30	0.9		
DMR1:93960001	1	93960001	4000	1	8.44E-13	38	0.95		
DMR1:94205701	1	94205701	1300	1	2.47E-11	4	0.3	AABR07003033.1	Unknown
DMR1:99114201	1	99114201	900	1	3.63E-10	5	0.55	Vom2r38	Receptor
DMR1:100327801	1	100327801	5000	1	4.71E-10	79	1.58	Rn60_1_1004.2;Shank1	Unknown;Development
DMR1:109791401	1	109791401	2500	2	2.18E-11	8	0.32		
DMR1:112121601	1	112121601	500	1	4.86E-11	3	0.6		
DMR1:119683801	1	119683801	3500	1	3.49E-12	12	0.34		
DMR1:119837801	1	119837801	3300	1	2.63E-10	20	0.6		
DMR1:128403801	1	128403801	4000	1	9.32E-10	45	1.12	LOC103691157	Unknown
DMR1:129366701	1	129366701	1800	1	2.93E-10	9	0.5		
DMR1:130924301	1	130924301	800	1	2.31E-12	3	0.37		
DMR1:136999101	1	136999101	2100	1	4.87E-10	17	0.8		
DMR1:138693801	1	138693801	300	1	1.78E-11	1	0.33	Agbl1	Signaling
DMR1:140047001	1	140047001	6700	1	4.68E-11	54	0.8	Ntrk3	Receptor
DMR1:151067401	1	151067401	2600	1	8.13E-10	17	0.65	Tyr	Metabolism
DMR1:162246501	1	162246501	2200	1	4.92E-10	3	0.13	Gab2	Receptor
DMR1:166496901	1	166496901	10200	1	4.05E-13	110	1.07	Arap1	Signaling
DMR1:170850801	1	170850801	4500	1	9.70E-12	21	0.46	Olr213;Olr214	Receptor
DMR1:175278301	1	175278301	7200	1	3.55E-10	46	0.63	Sbf2	Epigenetic
DMR1:178896201	1	178896201	5700	1	1.42E-10	81	1.42	Spon1	Growth Factors & Cytokines
DMR1:179064701	1	179064701	1000	1	1.37E-11	9	0.9		
DMR1:179556401	1	179556401	4000	1	2.57E-11	31	0.77		
DMR1:179621301	1	179621301	500	1	1.89E-11	3	0.6		
DMR1:179747201	1	179747201	1500	1	5.76E-10	8	0.53		
DMR1:190007001	1	190007001	1000	1	1.58E-12	4	0.4	Abca14	Transport
DMR1:191076201	1	191076201	4000	1	5.99E-12	41	1.02	5S_rRNA;Otoa	Unknown;Extracellular Matrix
DMR1:191092701	1	191092701	2000	3	4.46E-14	14	0.7	5S_rRNA;Otoa	Unknown;Extracellular Matrix
DMR1:195528701	1	195528701	3700	1	4.06E-13	15	0.4		
DMR1:203309401	1	203309401	6200	1	8.18E-10	31	0.5		
DMR1:207420701	1	207420701	1000	1	1.72E-10	1	0.1	Dock1	Signaling
DMR1:211744901	1	211744901	4500	1	4.50E-11	39	0.86	Inpp5a	Signaling
DMR1:215067901	1	215067901	3500	1	1.78E-12	31	0.88	NA	NA
DMR1:215633201	1	215633201	5200	1	2.03E-10	24	0.46	Lsp1	Cytoskeleton

DMR1:218237101	1	218237101	4000	1	9.19E-12	11	0.27		
DMR1:222047201	1	222047201	3000	1	6.09E-11	36	1.2		
DMR1:224212301	1	224212301	2300	1	6.13E-10	23	1		
DMR1:230320401	1	230320401	1900	1	8.34E-10	5	0.26	AC117862.1	Unknown
DMR1:231054901	1	231054901	1300	1	3.58E-10	7	0.53		
DMR1:236136001	1	236136001	2000	1	2.37E-12	12	0.6	Pcsk5	Protease
DMR1:237353301	1	237353301	7000	1	4.48E-12	35	0.5		
DMR1:241641601	1	241641601	2400	1	1.43E-10	5	0.2	Apba1	Development
DMR1:245067601	1	245067601	2100	1	4.76E-11	13	0.61		
DMR1:251829701	1	251829701	1400	1	4.64E-10	9	0.64	Rnls;AC123495.3	Metabolism;Unknown
DMR1:253368601	1	253368601	1100	1	6.30E-11	12	1.09		
DMR1:255440501	1	255440501	1300	1	3.49E-11	14	1.07		
DMR1:256360201	1	256360201	1500	1	8.86E-11	11	0.73	Cyp26c1;Exoc6	Metabolism;Transport
DMR1:261959601	1	261959601	8200	1	2.30E-11	131	1.59	Pyroxd2	Metabolism
DMR1:263282501	1	263282501	2200	3	3.78E-12	24	1.09		
DMR1:264052601	1	264052601	1800	1	3.96E-10	21	1.16	Scd2	Metabolism
DMR1:268949701	1	268949701	1800	1	9.75E-10	6	0.33		
DMR1:273531401	1	273531401	3100	1	4.46E-10	45	1.45		
DMR1:278123401	1	278123401	700	1	9.60E-10	4	0.57		
DMR1:278680401	1	278680401	7100	1	3.95E-11	70	0.98	Atrnl1	Signaling
DMR1:280686201	1	280686201	2500	1	3.49E-15	25	1		
DMR1:281947501	1	281947501	2600	5	3.74E-15	36	1.38		
DMR1:281951401	1	281951401	2200	2	4.77E-13	20	0.9		
DMR1:282343901	1	282343901	2700	1	9.60E-10	15	0.55	Grk5	Signaling
DMR2:2721101	2	2721101	3200	1	7.01E-10	21	0.65	AABR07072671.1;Spata9	Unknown;Development
DMR2:4669101	2	4669101	1500	1	2.04E-10	8	0.53		
DMR2:15642101	2	15642101	3100	4	1.09E-10	11	0.35		
DMR2:18068101	2	18068101	700	1	7.25E-11	0	0	Edil3	Extracellular Matrix
DMR2:24728801	2	24728801	12700	1	1.88E-10	119	0.93	Pde8b	Signaling
DMR2:28272801	2	28272801	2700	1	1.37E-11	39	1.44	AABR07007789.1	Unknown
DMR2:29247601	2	29247601	2400	1	3.67E-10	32	1.33	LOC684444	Unknown
DMR2:32283501	2	32283501	2500	1	1.02E-10	19	0.76		
DMR2:34115301	2	34115301	5300	1	1.94E-11	50	0.94	Nln	Protease
DMR2:34526101	2	34526101	3600	1	3.37E-11	17	0.47	Adamts6	Protease
DMR2:36737701	2	36737701	1500	1	1.37E-12	6	0.4		
DMR2:38406201	2	38406201	900	2	2.93E-10	5	0.55		
DMR2:41657401	2	41657401	700	1	1.03E-10	2	0.28	Rab3c	Signaling
DMR2:41674301	2	41674301	3700	1	1.89E-10	25	0.67	Rab3c	Signaling
DMR2:53737201	2	53737201	4500	1	6.26E-10	43	0.95		
DMR2:54695701	2	54695701	1600	1	7.45E-11	10	0.62	Mroh2b;AABR07008372.1	Development;Unknown
DMR2:56276201	2	56276201	700	1	4.20E-10	4	0.57		
DMR2:56731601	2	56731601	2300	2	8.79E-14	9	0.39		
DMR2:57279101	2	57279101	1400	1	4.03E-10	4	0.28	RGD1310081	Unknown
DMR2:61170801	2	61170801	700	1	1.72E-10	0	0	Adamts12	Protease
DMR2:75254901	2	75254901	2100	1	4.16E-11	11	0.52		
DMR2:76322501	2	76322501	2400	1	8.80E-10	10	0.41		
DMR2:77861301	2	77861301	4100	1	1.63E-12	43	1.04	Myo10	Cytoskeleton
DMR2:81327101	2	81327101	1700	1	5.32E-10	8	0.47		
DMR2:82501701	2	82501701	1800	1	9.01E-12	9	0.5	AABR07008991.1	Unknown
DMR2:86804301	2	86804301	2800	1	1.88E-11	7	0.25		
DMR2:90062201	2	90062201	4900	1	1.10E-10	23	0.46		
DMR2:100381601	2	100381601	1300	1	4.01E-11	3	0.23	Hnf4g	Transcription
DMR2:104705401	2	104705401	4500	1	2.62E-10	16	0.35		
DMR2:111541101	2	111541101	1600	1	2.68E-10	16	1	Nlgn1	Signaling
DMR2:113033001	2	113033001	1100	2	3.39E-11	3	0.27	Tnfsf10	Apoptosis
DMR2:114231401	2	114231401	5800	1	4.38E-10	50	0.86	Tnik	Signaling
DMR2:114916401	2	114916401	700	1	3.35E-10	6	0.85		
DMR2:115382201	2	115382201	500	1	2.58E-10	1	0.2		
DMR2:116876001	2	116876001	3700	1	3.61E-10	22	0.59	Mir551b	Epigenetic
DMR2:116907601	2	116907601	800	1	1.59E-11	5	0.62		
DMR2:121350001	2	121350001	3000	2	2.28E-10	22	0.73	Rn50_2_1411.1	Unknown
DMR2:125109901	2	125109901	500	1	6.21E-10	2	0.4		
DMR2:130557201	2	130557201	800	1	3.55E-12	2	0.25		
DMR2:138802901	2	138802901	3400	1	3.63E-11	37	1.08		
DMR2:139531801	2	139531801	1800	2	2.32E-11	9	0.5	AABR07010544.1;Slc7a11	Unknown;Transport
DMR2:140945801	2	140945801	2300	1	3.47E-10	20	0.86		
DMR2:141750401	2	141750401	4300	1	3.27E-10	40	0.93		

DMR2:144024101	2	144024101	1200	1	2.49E-10	9	0.75		
DMR2:144330501	2	144330501	1400	1	1.34E-10	18	1.28	Sertm1	Unknown
DMR2:146235601	2	146235601	2500	2	1.24E-10	19	0.76		
DMR2:148522501	2	148522501	1100	1	5.42E-10	4	0.36		
DMR2:150963101	2	150963101	700	1	6.76E-10	3	0.42		
DMR2:154694901	2	154694901	3800	1	4.40E-16	21	0.55	Vom2r46	Receptor
DMR2:155566601	2	155566601	4200	2	1.75E-11	12	0.28		
DMR2:158082601	2	158082601	500	1	1.49E-13	2	0.4	Veph1	Unknown
DMR2:159736301	2	159736301	900	1	5.57E-11	5	0.55		
DMR2:164798301	2	164798301	1700	1	8.00E-10	5	0.29		
DMR2:168006001	2	168006001	800	1	9.80E-10	2	0.25	AABR07011746.1	Unknown
DMR2:174981001	2	174981001	1300	1	6.01E-10	3	0.23	Fstl5	Hormone
DMR2:178902701	2	178902701	1200	1	1.49E-10	14	1.16		
DMR2:183639601	2	183639601	4900	3	6.04E-13	48	0.97		
DMR2:184475801	2	184475801	600	1	4.02E-10	0	0		
DMR2:184613101	2	184613101	3400	1	8.14E-10	33	0.97	Gatb	Metabolism
DMR2:186272601	2	186272601	600	1	2.00E-10	1	0.16		
DMR2:186538101	2	186538101	3600	1	6.22E-10	33	0.91		
DMR2:189908501	2	189908501	1400	1	6.44E-10	7	0.5	S100a1;S100a13;Chtop	Signaling;Epigenetic
DMR2:195282101	2	195282101	1600	1	1.61E-11	4	0.25	RGD1559714	Unknown
DMR2:205821701	2	205821701	1000	1	4.58E-10	9	0.9	Trim33	Transcription
DMR2:206121901	2	206121901	3400	1	3.25E-10	75	2.2	Syt6	Transport
DMR2:206609801	2	206609801	700	2	7.53E-12	2	0.28	Magi3	Signaling
DMR2:207705701	2	207705701	5300	1	2.52E-10	41	0.77		
DMR2:208443301	2	208443301	2200	1	7.54E-10	21	0.95	LOC100911453	Epigenetic;Receptor
DMR2:215058701	2	215058701	1400	1	6.24E-10	3	0.21		
DMR2:215108101	2	215108101	1600	1	7.72E-10	11	0.68		
DMR2:215244801	2	215244801	1700	1	3.59E-11	10	0.58		
DMR2:218782201	2	218782201	500	1	6.24E-10	27	5.4		
DMR2:223328201	2	223328201	5500	2	1.30E-12	46	0.83	Ptbp2	Translation
DMR2:228968501	2	228968501	3000	1	1.41E-10	18	0.6		
DMR2:231812401	2	231812401	1200	1	1.74E-10	15	1.25		
DMR2:231815001	2	231815001	5700	4	2.61E-13	59	1.03		
DMR2:232630301	2	232630301	5200	6	4.93E-18	48	0.92	AABR07013304.1	Unknown
DMR2:232756201	2	232756201	2600	1	1.82E-10	37	1.42		
DMR2:235496901	2	235496901	1600	1	1.23E-14	4	0.25		
DMR2:239109401	2	239109401	3600	2	2.32E-10	40	1.11		
DMR2:239701601	2	239701601	5800	1	3.06E-13	33	0.56		
DMR2:240758201	2	240758201	400	2	4.70E-10	6	1.5	Manba	Golgi
DMR2:243312701	2	243312701	5700	4	3.37E-15	93	1.63		
DMR2:243526001	2	243526001	900	1	5.14E-11	5	0.55	Adh7	Unknown
DMR2:243833101	2	243833101	2500	2	4.83E-11	13	0.52	Eif4e	Translation
DMR2:246328401	2	246328401	2700	1	1.09E-10	12	0.44		
DMR2:249657701	2	249657701	900	2	2.22E-13	6	0.66		
DMR2:255663301	2	255663301	2200	1	3.88E-10	13	0.59		
DMR2:255750001	2	255750001	2200	2	6.28E-18	1	0.04		
DMR2:259013401	2	259013401	1900	1	3.10E-10	18	0.94		
DMR2:260753401	2	260753401	3700	2	3.89E-13	32	0.86		
DMR2:260959301	2	260959301	4000	1	6.72E-11	28	0.7		
DMR2:261000501	2	261000501	2000	2	2.05E-10	19	0.95	Erich3	Unknown
DMR2:261360701	2	261360701	20200	2	6.50E-12	620	3.06	Fpgt	Signaling
DMR2:263529001	2	263529001	3800	1	8.57E-10	31	0.81	AABR07013922.2;Negr1	Unknown;Growth Factors & Cytokir
DMR2:265103601	2	265103601	300	1	7.10E-11	4	1.33	Lrrc7	Unknown
DMR3:8832601	3	8832601	2100	1	3.60E-10	24	1.14	Lrrc8a;Phyhd1	Unknown;Metabolism
DMR3:14002801	3	14002801	1300	1	2.71E-12	18	1.38	Traf1	Signaling
DMR3:15086601	3	15086601	6100	2	3.33E-10	69	1.13	Ttll11	Cytoskeleton
DMR3:16882501	3	16882501	900	1	3.72E-16	3	0.33	LOC100361009	Unknown
DMR3:17575501	3	17575501	500	1	5.63E-10	7	1.4		
DMR3:18638701	3	18638701	2300	2	9.99E-11	18	0.78		
DMR3:29681301	3	29681301	1200	1	2.56E-10	8	0.66	Gtdc1	Metabolism
DMR3:30185301	3	30185301	6100	1	1.69E-13	38	0.62		
DMR3:33877901	3	33877901	1600	1	4.17E-11	7	0.43		
DMR3:35479901	3	35479901	5200	1	3.22E-15	54	1.03		
DMR3:35527801	3	35527801	4800	2	3.15E-13	32	0.66	AABR07052082.1	Unknown
DMR3:37521701	3	37521701	1600	1	7.20E-10	16	1		
DMR3:41676001	3	41676001	2900	1	4.77E-10	6	0.2		
DMR3:43070401	3	43070401	2100	1	2.15E-10	15	0.71		

DMR3:46584701	3	46584701	7700	2	3.23E-10	71	0.92	Pla2r1	Receptor
DMR3:50944601	3	50944601	3300	1	2.51E-10	24	0.72	Grb14	Receptor
DMR3:60049901	3	60049901	1800	1	9.08E-10	25	1.38	Gpr155;Scrn3	Receptor;Unknown
DMR3:61263101	3	61263101	2700	1	8.98E-11	29	1.07		
DMR3:69076801	3	69076801	500	1	3.37E-10	5	1		
DMR3:71103101	3	71103101	300	1	8.64E-11	1	0.33	AABR07052730.2	Unknown
DMR3:72589201	3	72589201	2200	1	3.88E-10	18	0.81	Lrrc55	Unknown
DMR3:73554501	3	73554501	6500	1	5.15E-10	35	0.53	Olr486	Receptor
DMR3:78394301	3	78394301	1600	2	2.97E-12	6	0.37		
DMR3:81977801	3	81977801	2100	1	5.14E-11	9	0.42		
DMR3:83355401	3	83355401	1400	1	9.88E-10	17	1.21		
DMR3:87487201	3	87487201	3200	1	1.14E-12	9	0.28		
DMR3:91825401	3	91825401	2600	1	3.84E-11	30	1.15	Ldlrad3	Receptor
DMR3:97697001	3	97697001	1900	1	2.43E-10	12	0.63		
DMR3:105739001	3	105739001	1700	1	1.10E-11	3	0.17		
DMR3:114335701	3	114335701	1400	1	4.06E-10	5	0.35		
DMR3:114705401	3	114705401	3000	2	1.96E-13	26	0.86	Gatm	Metabolism
DMR3:115472101	3	115472101	1900	2	2.31E-12	15	0.78		
DMR3:117375901	3	117375901	1200	1	3.50E-10	13	1.08	Myef2	Development
DMR3:117752101	3	117752101	2700	1	2.12E-10	49	1.81	Fbn1	Development
DMR3:120824001	3	120824001	1000	1	1.58E-10	4	0.4		
DMR3:121966701	3	121966701	1900	1	8.64E-11	7	0.36	RGD1566226	Unknown
DMR3:129986101	3	129986101	2500	1	2.47E-16	29	1.16	Slx4ip	Unknown
DMR3:133835601	3	133835601	1200	1	2.64E-11	5	0.41		
DMR3:137227601	3	137227601	2500	1	1.56E-10	15	0.6		
DMR3:140204301	3	140204301	2100	1	3.37E-14	18	0.85	Rn50_3_1467.1	Unknown
DMR3:154184901	3	154184901	900	1	5.36E-11	6	0.66		
DMR3:158497801	3	158497801	900	1	2.16E-11	6	0.66		
DMR3:160808001	3	160808001	2100	1	1.73E-10	25	1.19	Slpi	Protease
DMR3:166085001	3	166085001	1500	1	3.27E-10	26	1.73		
DMR3:166379801	3	166379801	5000	1	4.73E-10	57	1.14		
DMR3:166657201	3	166657201	4200	1	7.70E-10	86	2.04		
DMR3:168717501	3	168717501	3100	1	8.26E-10	34	1.09		
DMR3:168888801	3	168888801	1500	1	1.62E-12	21	1.4		
DMR3:170155701	3	170155701	3400	1	2.36E-10	47	1.38		
DMR3:170495401	3	170495401	5200	1	2.03E-10	86	1.65		
DMR3:172077701	3	172077701	4300	1	2.93E-10	51	1.18		
DMR3:174281701	3	174281701	1500	1	2.85E-11	14	0.93		
DMR3:175174101	3	175174101	1800	1	6.82E-10	19	1.05	Cdh4	Extracellular Matrix
DMR4:1936601	4	1936601	1700	1	3.45E-10	5	0.29		
DMR4:5271401	4	5271401	3300	1	2.55E-10	27	0.81		
DMR4:9239201	4	9239201	1100	3	6.03E-17	3	0.27		
DMR4:9407001	4	9407001	800	1	6.84E-11	3	0.37	Reln	Protease
DMR4:22733601	4	22733601	4800	1	4.72E-11	65	1.35		
DMR4:25520701	4	25520701	2300	1	4.50E-11	44	1.91	Steap2	Golgi
DMR4:25783801	4	25783801	6300	1	4.59E-10	45	0.71		
DMR4:32814401	4	32814401	6600	1	1.52E-11	45	0.68		
DMR4:33798701	4	33798701	800	1	9.26E-15	4	0.5		
DMR4:36035701	4	36035701	6300	1	7.38E-10	23	0.36		
DMR4:37863101	4	37863101	4100	1	5.03E-10	36	0.87		
DMR4:40449601	4	40449601	1400	1	7.70E-10	11	0.78		
DMR4:42825201	4	42825201	900	1	8.27E-10	4	0.44	Cftr	Transport
DMR4:44469701	4	44469701	6700	1	8.68E-10	41	0.61		
DMR4:45800801	4	45800801	500	1	3.15E-10	2	0.4		
DMR4:49903101	4	49903101	800	2	1.66E-11	4	0.5		
DMR4:56279601	4	56279601	2300	1	1.57E-11	27	1.17		
DMR4:58316801	4	58316801	4100	1	4.87E-12	43	1.04		
DMR4:67622001	4	67622001	5100	2	8.27E-13	37	0.72		
DMR4:67903001	4	67903001	1500	1	8.27E-12	16	1.06		
DMR4:73375101	4	73375101	1000	1	6.93E-11	8	0.8		
DMR4:77153301	4	77153301	400	1	2.00E-10	3	0.75		
DMR4:82500901	4	82500901	1300	1	1.44E-10	6	0.46		
DMR4:87753901	4	87753901	200	1	1.15E-11	0	0	Vom1r73	Receptor
DMR4:87756101	4	87756101	200	1	4.20E-12	0	0	Vom1r73	Receptor
DMR4:91091901	4	91091901	600	1	2.12E-11	6	1		
DMR4:93129201	4	93129201	4800	1	2.90E-12	25	0.52		
DMR4:105349501	4	105349501	4500	1	1.32E-10	15	0.33		

DMR4:111922301	4	111922301	2100	1	7.31E-11	8	0.38	AABR07061251.1	Unknown
DMR4:112995301	4	112995301	700	1	2.58E-10	5	0.71		
DMR4:123672601	4	123672601	3000	1	9.76E-10	25	0.83	Slc6a6	Transport
DMR4:126781301	4	126781301	1300	1	7.43E-12	18	1.38		
DMR4:130474101	4	130474101	1300	2	3.73E-11	13	1		
DMR4:130504501	4	130504501	1000	1	7.75E-10	3	0.3		
DMR4:136596201	4	136596201	1800	2	4.94E-11	10	0.55	Cntn6	Extracellular Matrix
DMR4:137359701	4	137359701	300	1	4.30E-12	5	1.66		
DMR4:154018601	4	154018601	900	1	4.22E-10	3	0.33	Iqsec3	Signaling
DMR4:158921201	4	158921201	1300	3	7.38E-12	1	0.07		
DMR4:159242601	4	159242601	900	2	1.28E-11	1	0.11		
DMR4:159962701	4	159962701	5400	1	2.29E-10	43	0.79		
DMR4:160314501	4	160314501	3200	1	1.45E-10	36	1.12	Prmt8	Epigenetic
DMR4:165078201	4	165078201	1100	1	2.22E-10	7	0.63		
DMR4:165616801	4	165616801	2700	2	4.91E-11	46	1.7	Ybx3;AABR07062228.1	Unknown
DMR4:167434001	4	167434001	8500	1	8.70E-10	82	0.96	LOC100363344	Unknown
DMR4:168765601	4	168765601	1000	1	9.12E-11	9	0.9	Ddx47	Transcription
DMR4:171521001	4	171521001	1200	1	1.80E-10	20	1.66	Eps8	Signaling
DMR4:171568701	4	171568701	3200	1	3.34E-12	42	1.31	Eps8	Signaling
DMR4:171586701	4	171586701	5200	1	2.15E-10	82	1.57	Eps8	Signaling
DMR4:171657101	4	171657101	4900	1	2.57E-10	62	1.26		
DMR4:171970701	4	171970701	3300	1	1.99E-10	27	0.81	Slc15a5	Transport
DMR4:175319301	4	175319301	700	1	4.49E-10	5	0.71		
DMR4:175402201	4	175402201	3200	1	7.67E-10	31	0.96		
DMR4:175422801	4	175422801	4100	2	3.04E-12	44	1.07	Pde3a	Signaling
DMR4:176451001	4	176451001	1500	1	3.91E-10	5	0.33	AC144687.1;Slco1a2	Unknown;Transport
DMR4:176665901	4	176665901	2900	1	3.42E-10	37	1.27	Gys2	Metabolism
DMR4:177629301	4	177629301	1400	1	2.52E-11	10	0.71		
DMR4:180806101	4	180806101	2400	1	4.93E-10	19	0.79	AABR07062513.3	Unknown
DMR4:182153401	4	182153401	1400	1	6.62E-12	26	1.85		
DMR4:182306901	4	182306901	6100	1	1.75E-12	73	1.19		
DMR4:182336301	4	182336301	1300	1	3.08E-11	8	0.61	AABR07062544.1	Unknown
DMR5:3629601	5	3629601	3200	1	4.81E-10	14	0.43	AABR07046725.1	Unknown
DMR5:7638601	5	7638601	4800	1	8.43E-10	26	0.54	Prex2	Signaling
DMR5:9199501	5	9199501	2000	1	1.81E-10	16	0.8		
DMR5:16367001	5	16367001	10800	1	1.86E-11	75	0.69	Tmem68	Unknown
DMR5:22247601	5	22247601	800	1	7.98E-13	0	0	AABR07047174.1	Unknown
DMR5:27545101	5	27545101	1000	1	1.07E-10	3	0.3		
DMR5:42115301	5	42115301	1100	1	1.27E-10	2	0.18		
DMR5:43322701	5	43322701	1500	1	1.02E-10	24	1.6		
DMR5:45960601	5	45960601	1000	1	8.75E-10	3	0.3		
DMR5:52736301	5	52736301	2800	1	8.83E-10	19	0.67	LOC298111	Immune
DMR5:53542801	5	53542801	1800	1	3.52E-11	5	0.27		
DMR5:55678001	5	55678001	1300	1	6.83E-10	6	0.46		
DMR5:56676401	5	56676401	4100	3	2.95E-18	59	1.43	AABR07048010.1	Unknown
DMR5:58220201	5	58220201	4800	2	3.80E-13	62	1.29	AC110351.2	Unknown
DMR5:62383801	5	62383801	1600	1	4.50E-12	14	0.87	Gabbr2	Receptor
DMR5:63459301	5	63459301	3900	1	7.87E-11	43	1.1		
DMR5:65463601	5	65463601	2900	1	6.70E-18	21	0.72		
DMR5:70786001	5	70786001	1700	2	1.31E-11	17	1		
DMR5:70834901	5	70834901	600	1	1.23E-10	0	0	AABR07048281.1	Unknown
DMR5:74897201	5	74897201	100	1	3.77E-10	5	5	Rn50_5_0791.1;Akap2	Unknown;Signaling
DMR5:75782701	5	75782701	1100	1	2.05E-11	4	0.36		
DMR5:77935401	5	77935401	5900	2	5.10E-10	46	0.77	Zfp37	Transcription
DMR5:78065901	5	78065901	1200	1	1.52E-12	2	0.16	Rn50_5_0814.8	Unknown
DMR5:81734201	5	81734201	2100	1	5.10E-11	9	0.42	AABR07048540.1;Astn2	Unknown
DMR5:86436601	5	86436601	1500	1	4.93E-10	15	1	Cdk5rap2	Signaling
DMR5:86794001	5	86794001	7200	1	8.52E-10	21	0.29		
DMR5:86815201	5	86815201	9500	1	1.85E-10	39	0.41		
DMR5:86863201	5	86863201	4800	2	1.32E-12	56	1.16	AABR07048653.1	Unknown
DMR5:87607101	5	87607101	2800	1	8.49E-10	9	0.32		
DMR5:91690401	5	91690401	3600	1	2.74E-10	8	0.22		
DMR5:96212101	5	96212101	3600	1	8.32E-10	11	0.3		
DMR5:96669401	5	96669401	1700	1	1.07E-10	11	0.64		
DMR5:97547001	5	97547001	4100	1	4.44E-13	24	0.58		
DMR5:98682501	5	98682501	4400	1	3.17E-13	64	1.45	AABR07048957.1	Unknown
DMR5:98715901	5	98715901	7300	1	2.81E-10	46	0.63	AABR07048966.1	Unknown

DMR5:98726801	5	98726801	1800	1	4.68E-12	17	0.94	AABR07048966.1	Unknown
DMR5:105217201	5	105217201	7400	1	8.38E-12	75	1.01	Acer2;LOC100911372	Metabolism;Translation
DMR5:106375601	5	106375601	1500	1	4.69E-10	9	0.6		
DMR5:108735001	5	108735001	700	3	2.14E-15	1	0.14		
DMR5:114122201	5	114122201	3200	1	3.74E-10	17	0.53	AABR07049288.1	Unknown
DMR5:114817501	5	114817501	1200	1	4.98E-10	1	0.08	Fggy	Signaling
DMR5:117051701	5	117051701	2500	1	7.59E-10	18	0.72	Patj	Development
DMR5:117648101	5	117648101	4700	1	3.44E-12	24	0.51	Dock7	Signaling
DMR5:123369501	5	123369501	1300	1	4.33E-11	5	0.38		
DMR5:123388001	5	123388001	3000	1	7.64E-10	18	0.6		
DMR5:123512801	5	123512801	2800	1	7.20E-10	14	0.5		
DMR5:128331701	5	128331701	1800	1	4.11E-11	5	0.27	Zfyve9	Transcription
DMR5:130764601	5	130764601	2300	1	5.47E-10	7	0.3		
DMR5:136361501	5	136361501	3700	1	6.29E-10	39	1.05		
DMR5:141469601	5	141469601	2800	1	3.55E-11	24	0.85		
DMR5:143391901	5	143391901	600	1	8.26E-10	2	0.33		
DMR5:150786301	5	150786301	1900	1	3.89E-11	14	0.73	Eya3	Signaling
DMR5:151603901	5	151603901	3300	1	1.57E-10	40	1.21	Slc9a1	Transport
DMR5:154150801	5	154150801	2200	1	1.57E-10	28	1.27	Myom3	Cytoskeleton
DMR5:155483101	5	155483101	2600	1	7.26E-10	27	1.03		
DMR5:157021501	5	157021501	600	1	3.35E-10	13	2.16	Vwa5b1	Development
DMR5:157104701	5	157104701	2100	1	8.52E-11	29	1.38		
DMR5:159373201	5	159373201	1700	1	7.09E-11	21	1.23	Padi1	Metabolism
DMR5:161428901	5	161428901	1800	1	3.76E-12	34	1.88		
DMR5:167745001	5	167745001	2100	1	9.65E-10	11	0.52		
DMR5:170764301	5	170764301	4100	1	3.94E-10	61	1.48	AABR07050600.1	Unknown
DMR5:170937801	5	170937801	2400	1	2.05E-11	20	0.83		
DMR5:172107801	5	172107801	1300	1	5.85E-11	7	0.53		
DMR5:172126901	5	172126901	5600	1	2.22E-10	64	1.14		
DMR5:172222901	5	172222901	4800	1	3.60E-10	57	1.18		
DMR5:172780801	5	172780801	1300	1	3.75E-10	8	0.61	AABR07050652.1	Unknown
DMR6:2667001	6	2667001	8800	4	8.35E-16	61	0.69	AABR07062672.2	Unknown
DMR6:4344601	6	4344601	4700	1	4.01E-11	35	0.74	Slc8a1	Transport
DMR6:5627101	6	5627101	800	1	9.82E-10	0	0		
DMR6:7134801	6	7134801	3000	1	8.52E-11	30	1		
DMR6:12659801	6	12659801	400	1	8.52E-11	4	1		
DMR6:17291001	6	17291001	2100	2	2.87E-12	13	0.61		
DMR6:17940501	6	17940501	1000	1	2.61E-13	3	0.3		
DMR6:18379701	6	18379701	1100	1	6.12E-10	5	0.45		
DMR6:29928201	6	29928201	900	1	6.74E-10	1	0.11	5S_rRNA	Unknown
DMR6:35189101	6	35189101	2300	1	6.11E-10	21	0.91		
DMR6:35740101	6	35740101	1000	1	4.15E-10	1	0.1		
DMR6:37891101	6	37891101	400	1	1.67E-10	7	1.75		
DMR6:39013401	6	39013401	700	1	2.03E-11	2	0.28		
DMR6:41209301	6	41209301	3100	1	1.91E-13	28	0.9		
DMR6:42403401	6	42403401	500	1	8.67E-10	4	0.8		
DMR6:46352901	6	46352901	900	1	1.65E-10	2	0.22		
DMR6:46373601	6	46373601	500	1	1.43E-10	5	1		
DMR6:49985601	6	49985601	2400	1	3.17E-10	13	0.54		
DMR6:50481001	6	50481001	1400	2	2.26E-11	6	0.42		
DMR6:50972201	6	50972201	2400	1	5.79E-11	12	0.5	Cog5	Golgi
DMR6:56742701	6	56742701	1500	1	4.41E-14	2	0.13	Vom2r49;AABR07063940.1	Receptor;Unknown
DMR6:58247501	6	58247501	3000	1	6.41E-10	19	0.63	Dgkb	Signaling
DMR6:70341101	6	70341101	400	1	5.78E-10	2	0.5		
DMR6:77150101	6	77150101	3400	1	4.14E-10	26	0.76	AABR07064369.1	Unknown
DMR6:77595401	6	77595401	2200	1	3.75E-10	24	1.09		
DMR6:80382001	6	80382001	8300	1	3.25E-10	65	0.78		
DMR6:86543601	6	86543601	600	1	1.26E-10	3	0.5		
DMR6:88087001	6	88087001	3400	1	3.43E-11	15	0.44	AABR07064635.1	Unknown
DMR6:96923401	6	96923401	3100	2	6.53E-13	39	1.25		
DMR6:105320901	6	105320901	3200	1	2.71E-11	14	0.43	AABR07065042.1;Med6	Unknown;Transcription
DMR6:110504501	6	110504501	4700	1	2.07E-10	32	0.68	AABR07065115.1	Unknown
DMR6:113631201	6	113631201	3000	1	8.22E-10	26	0.86	Rn60_6_1137.1;Nrxn3	Unknown;Receptor
DMR6:116696301	6	116696301	400	1	2.36E-11	2	0.5		
DMR6:118107901	6	118107901	2000	1	5.39E-12	9	0.45		
DMR6:118512401	6	118512401	1100	1	3.17E-10	1	0.09	AABR07065274.1	Unknown
DMR6:130188101	6	130188101	1400	1	4.88E-10	16	1.14	AABR07065476.2	Unknown

DMR6:131338601	6	131338601	2000	2	5.54E-11	12	0.6		
DMR6:131443101	6	131443101	2400	2	1.41E-12	13	0.54		
DMR6:132742601	6	132742601	1200	1	1.68E-10	8	0.66	Mir345;Slc25a29	Epigenetic;Transport
DMR6:134447101	6	134447101	1400	1	1.57E-10	12	0.85		
DMR6:134711101	6	134711101	6200	1	8.23E-13	66	1.06	AABR07065566.1	Unknown
DMR6:140191801	6	140191801	2600	1	2.41E-11	9	0.34		
DMR6:142855501	6	142855501	3700	1	4.72E-12	14	0.37	AABR07065821.1	Unknown
DMR7:12754101	7	12754101	3900	1	2.84E-10	156	4	Grin3b;Palm;Abca7	Transport;Development
DMR7:18000601	7	18000601	4000	1	8.63E-11	14	0.35		
DMR7:20256101	7	20256101	2600	1	4.82E-10	12	0.46	LOC300308	Unknown
DMR7:21299901	7	21299901	5400	1	9.73E-10	25	0.46		
DMR7:21307201	7	21307201	1400	1	1.62E-10	8	0.57		
DMR7:21470201	7	21470201	500	1	7.38E-10	4	0.8		
DMR7:21620701	7	21620701	1900	2	2.90E-12	32	1.68		
DMR7:21686701	7	21686701	10400	1	1.19E-11	102	0.98		
DMR7:21704401	7	21704401	6900	1	2.99E-10	22	0.31		
DMR7:21753101	7	21753101	13900	1	2.96E-10	94	0.67		
DMR7:24695101	7	24695101	1500	2	3.45E-10	19	1.26		
DMR7:25106801	7	25106801	8100	1	4.38E-10	168	2.07	Nuak1	Signaling
DMR7:26663701	7	26663701	3500	1	1.21E-10	51	1.45	Chst11	Metabolism
DMR7:31359001	7	31359001	1800	1	6.72E-10	7	0.38	Anks1b	Receptor
DMR7:33346001	7	33346001	4500	1	3.63E-10	36	0.8		
DMR7:34347201	7	34347201	3900	1	2.17E-10	66	1.69	Hal	Metabolism
DMR7:35971201	7	35971201	5400	1	2.06E-10	72	1.33	Plxnc1	Signaling
DMR7:36433701	7	36433701	3200	1	6.44E-10	40	1.25		
DMR7:37778701	7	37778701	4700	1	6.72E-10	50	1.06		
DMR7:37923601	7	37923601	1200	2	2.03E-10	6	0.5		
DMR7:39943701	7	39943701	800	1	7.93E-16	0	0		
DMR7:40057401	7	40057401	13400	13	8.64E-14	100	0.74		
DMR7:40158201	7	40158201	5900	2	1.20E-12	39	0.66	Tmtc3	Metabolism
DMR7:40165601	7	40165601	900	1	6.70E-10	9	1	Tmtc3	Metabolism
DMR7:41731501	7	41731501	1800	1	6.82E-10	6	0.33		
DMR7:42440701	7	42440701	1600	1	7.68E-11	15	0.93		
DMR7:43606701	7	43606701	1000	1	1.16E-13	7	0.7	NA	NA
DMR7:45823101	7	45823101	1800	1	7.54E-10	11	0.61		
DMR7:51859601	7	51859601	1000	1	3.43E-12	8	0.8	Otogl	Unknown
DMR7:53461601	7	53461601	600	1	1.68E-11	6	1		
DMR7:54369201	7	54369201	2600	1	8.82E-10	11	0.42		
DMR7:55217801	7	55217801	2000	1	1.58E-10	6	0.3		
DMR7:60737401	7	60737401	500	1	1.36E-18	1	0.2	Mdm2	Metabolism
DMR7:62713701	7	62713701	2600	1	9.47E-12	36	1.38		
DMR7:65141501	7	65141501	5800	1	4.72E-10	64	1.1		
DMR7:66420801	7	66420801	1900	1	1.43E-10	8	0.42		
DMR7:67984801	7	67984801	7000	1	7.17E-11	44	0.6		
DMR7:70247401	7	70247401	2100	1	9.75E-11	12	0.57	AABR07057421.3	Unknown
DMR7:71770601	7	71770601	600	1	6.38E-10	3	0.5	Cpq	Metabolism
DMR7:74346001	7	74346001	700	1	4.23E-10	5	0.71	AABR07057495.1	Unknown
DMR7:76002201	7	76002201	4400	3	2.51E-13	51	1.15		
DMR7:77417701	7	77417701	7400	1	9.58E-10	77	1.04		
DMR7:82819201	7	82819201	2400	1	2.63E-10	14	0.58		
DMR7:83552401	7	83552401	3300	1	5.74E-11	10	0.3	Pkhd1l1;Ebag9	Development;Receptor
DMR7:88119201	7	88119201	1100	1	2.06E-12	0	0		
DMR7:88925301	7	88925301	700	1	2.84E-10	4	0.57		
DMR7:95072201	7	95072201	1400	1	6.04E-10	5	0.35	Col14a1	Cytoskeleton
DMR7:98451501	7	98451501	5800	1	1.25E-10	39	0.67	Fer1l6;AABR07058015.1	Transport;Unknown
DMR7:112374601	7	112374601	6100	1	2.10E-11	50	0.81		
DMR7:115098501	7	115098501	2100	2	3.77E-11	22	1.04		
DMR7:115110201	7	115110201	1100	1	4.81E-10	2	0.18	AABR07058412.3	Unknown
DMR7:115172601	7	115172601	6100	1	1.37E-10	89	1.45		
DMR7:115437601	7	115437601	900	1	6.98E-10	5	0.55		
DMR7:116008201	7	116008201	800	1	2.71E-10	3	0.37	AABR07058423.3	Unknown
DMR7:122371801	7	122371801	2600	1	1.21E-11	31	1.19	Mkl1	Unknown
DMR7:122507401	7	122507401	2200	1	8.01E-11	24	1.09		
DMR7:125085701	7	125085701	5000	1	3.04E-11	60	1.2	Samm50	Mitochondria
DMR7:135725401	7	135725401	4200	1	5.80E-10	42	1		
DMR7:136498801	7	136498801	2300	1	1.58E-10	16	0.69	AABR07058758.1	Unknown
DMR7:136589501	7	136589501	500	1	2.99E-11	1	0.2	Nell2	Signaling

DMR7:136641001	7	136641001	2900	1	6.78E-10	26	0.89	Nell2	Signaling
DMR7:138185101	7	138185101	4800	1	1.19E-10	67	1.39		
DMR7:138666001	7	138666001	4600	2	1.42E-11	45	0.97		
DMR7:142389801	7	142389801	2800	1	4.43E-12	44	1.57	Slc4a8	Transport
DMR7:142412201	7	142412201	2100	1	8.56E-10	18	0.85	Slc4a8	Transport
DMR7:143258101	7	143258101	3900	2	3.84E-12	17	0.43	Rn50_7_1411.2	Unknown
DMR8:5299901	8	5299901	600	1	2.38E-11	0	0	Dync2h1	Cytoskeleton
DMR8:15401301	8	15401301	1000	1	1.03E-10	5	0.5		
DMR8:16521701	8	16521701	300	1	2.41E-10	1	0.33		
DMR8:18517701	8	18517701	2600	1	8.85E-11	9	0.34	Muc16;AABR07069371.2	Cytoskeleton;Unknown
DMR8:18539801	8	18539801	1900	1	8.19E-11	3	0.15		
DMR8:21719601	8	21719601	4700	1	4.78E-11	50	1.06	Olfm2	Receptor
DMR8:31410701	8	31410701	2100	1	2.38E-11	23	1.09		
DMR8:43990101	8	43990101	2700	1	5.69E-12	14	0.51	Tmem225	Unknown
DMR8:45502601	8	45502601	700	1	1.79E-11	4	0.57		
DMR8:46673501	8	46673501	4600	2	1.43E-10	53	1.15	Tecta	Extracellular Matrix
DMR8:51270501	8	51270501	3600	1	1.53E-10	41	1.13		
DMR8:52290901	8	52290901	2700	1	1.35E-11	29	1.07		
DMR8:52478201	8	52478201	1700	1	1.94E-11	5	0.29		
DMR8:54654101	8	54654101	4500	1	2.40E-12	45	1		
DMR8:58590001	8	58590001	3300	1	7.15E-11	31	0.93	Tnfaip8l3	Apoptosis
DMR8:58597701	8	58597701	2400	1	7.42E-13	18	0.75	Tnfaip8l3	Apoptosis
DMR8:65768501	8	65768501	1900	1	1.57E-10	20	1.05	Uaca	Unknown
DMR8:70130501	8	70130501	4400	1	2.84E-11	59	1.34	Megf11	Extracellular Matrix
DMR8:71065101	8	71065101	1400	1	9.66E-10	9	0.64	Ankdd1a	Unknown
DMR8:73092501	8	73092501	2300	1	7.41E-11	30	1.3	Tln2	Cytoskeleton
DMR8:77752801	8	77752801	1700	1	2.09E-10	7	0.41		
DMR8:85135501	8	85135501	4800	7	1.60E-16	40	0.83		
DMR8:90693201	8	90693201	5600	1	1.55E-10	33	0.58		
DMR8:94622301	8	94622301	2100	1	1.29E-12	1	0.04		
DMR8:95225601	8	95225601	6800	1	2.54E-13	58	0.85		
DMR8:97774001	8	97774001	5100	1	6.03E-10	25	0.49		
DMR8:97783501	8	97783501	4900	1	6.85E-10	66	1.34		
DMR8:100162701	8	100162701	600	1	9.26E-10	3	0.5		
DMR8:102663401	8	102663401	3000	1	1.70E-10	12	0.4	Slc9a9	Transport
DMR8:103066101	8	103066101	3500	3	4.20E-13	19	0.54		
DMR8:107071001	8	107071001	3700	1	8.90E-10	43	1.16		
DMR8:109379801	8	109379801	2900	1	4.26E-10	35	1.2	Pccb	Metabolism
DMR8:113050201	8	113050201	4300	1	4.25E-10	49	1.13		
DMR8:114745601	8	114745601	3800	1	3.51E-11	19	0.5	Col6a4	Cytoskeleton
DMR8:114975401	8	114975401	3300	1	6.96E-11	33	1	Poc1a	Unknown
DMR8:119851201	8	119851201	3100	2	2.60E-12	33	1.06	Stac	Unknown
DMR8:119918501	8	119918501	1400	1	2.60E-11	9	0.64		
DMR8:125573501	8	125573501	2600	1	4.06E-10	41	1.57	Rbms3	Epigenetic
DMR8:126188001	8	126188001	1900	1	6.23E-10	17	0.89		
DMR8:128492501	8	128492501	6300	1	8.67E-10	75	1.19	Scn11a	Transport
DMR8:129323301	8	129323301	2900	1	9.51E-10	27	0.93		
DMR9:1431001	9	1431001	3000	1	1.90E-11	98	3.26	Tbc1d5	Signaling
DMR9:3731401	9	3731401	2200	3	2.61E-11	21	0.95		
DMR9:4080501	9	4080501	2300	1	2.64E-10	7	0.3	LOC100910057;Sult1c2	Metabolism
DMR9:18807501	9	18807501	800	1	1.89E-10	1	0.12	AABR07066838.1	Unknown
DMR9:19767001	9	19767001	1600	1	9.32E-10	14	0.87		
DMR9:19865801	9	19865801	800	1	3.34E-11	3	0.37	Cyp39a1	Metabolism
DMR9:20375701	9	20375701	2300	1	7.00E-11	26	1.13		
DMR9:26484801	9	26484801	4200	2	1.64E-10	26	0.61		
DMR9:35179601	9	35179601	1300	1	2.58E-10	2	0.15		
DMR9:37897101	9	37897101	3600	1	6.00E-10	53	1.47	Dst	Cell Junction
DMR9:38510101	9	38510101	5300	1	4.66E-11	59	1.11		
DMR9:39950801	9	39950801	1300	1	2.22E-10	0	0	Khdrbs2	Translation
DMR9:40205501	9	40205501	1800	1	2.91E-11	13	0.72		
DMR9:42711201	9	42711201	2200	1	4.98E-14	18	0.81	Uggt1	Metabolism
DMR9:43767901	9	43767901	2700	1	2.82E-10	31	1.14	Vwa3b	Development
DMR9:46157801	9	46157801	1800	1	8.67E-10	17	0.94	Tbc1d8	Signaling
DMR9:46620401	9	46620401	400	1	4.22E-11	3	0.75		
DMR9:49936601	9	49936601	900	1	7.21E-10	3	0.33	Fhl2	Transcription
DMR9:51654801	9	51654801	3100	1	8.16E-11	17	0.54		
DMR9:53847001	9	53847001	1200	1	2.72E-11	4	0.33		

DMR9:62281801	9	62281801	2100	1	7.62E-10	8	0.38	Plcl1	Signaling
DMR9:81146701	9	81146701	1600	3	4.44E-10	5	0.31		
DMR9:82836801	9	82836801	1100	1	1.76E-10	12	1.09	AABR07068085.2	Unknown
DMR9:95248701	9	95248701	300	1	1.65E-11	3	1	Ugt1a2	Metabolism
DMR9:97313401	9	97313401	4700	1	9.75E-10	37	0.78		
DMR9:98734601	9	98734601	2000	1	1.30E-11	33	1.65		
DMR9:100201401	9	100201401	3200	1	8.63E-10	61	1.9	Kif1a	Cytoskeleton
DMR9:101605201	9	101605201	5400	1	6.10E-13	26	0.48		
DMR9:103493101	9	103493101	1200	1	1.38E-12	5	0.41		
DMR9:104914901	9	104914901	1200	1	3.09E-11	3	0.25		
DMR9:111194901	9	111194901	1400	1	2.21E-10	5	0.35	Gin1	Development
DMR9:111332101	9	111332101	2000	1	6.47E-10	17	0.85	RGD1562136	Unknown
DMR9:116796701	9	116796701	5700	1	5.67E-11	64	1.12	L3mbtl4	Epigenetic
DMR9:118242601	9	118242601	1700	1	1.29E-10	14	0.82		
DMR9:118589301	9	118589301	2300	1	7.07E-10	14	0.6	Dlgap1	Signaling
DMR9:119444601	9	119444601	1600	1	1.50E-12	23	1.43	Myom1	Unknown
DMR9:120067701	9	120067701	1300	1	3.99E-11	8	0.61		
DMR10:2346201	10	2346201	2800	2	6.10E-14	5	0.17		
DMR10:4198001	10	4198001	3000	1	3.73E-10	33	1.1	Snx29	Cytoskeleton
DMR10:7036301	10	7036301	3600	1	3.34E-10	16	0.44	;Carhsp1	Unknown;Translation
DMR10:16390101	10	16390101	1800	1	5.92E-10	16	0.88		
DMR10:17949601	10	17949601	700	1	4.89E-10	3	0.42		
DMR10:19773501	10	19773501	3700	1	2.34E-10	47	1.27		
DMR10:20272501	10	20272501	4200	1	2.72E-10	76	1.8		
DMR10:20774601	10	20774601	200	1	2.43E-12	5	2.5	Wwc1	Unknown
DMR10:23799701	10	23799701	3100	1	1.03E-10	43	1.38	Ebf1	Transcription
DMR10:25226301	10	25226301	600	1	1.85E-10	6	1		
DMR10:27586701	10	27586701	1600	2	3.57E-12	7	0.43		
DMR10:29061001	10	29061001	1100	1	2.61E-10	19	1.72	C1qtnf2	Immune
DMR10:32214601	10	32214601	700	1	1.39E-11	8	1.14	Sgcd	Cytoskeleton
DMR10:38478501	10	38478501	6800	2	2.04E-10	91	1.33	Fstl4	Hormone
DMR10:39094501	10	39094501	4200	1	5.95E-10	46	1.09	AC135771.1	Unknown
DMR10:39220101	10	39220101	3200	2	5.41E-13	21	0.65	Slc22a5	Transport
DMR10:41268001	10	41268001	3600	1	5.41E-10	14	0.38		
DMR10:42466001	10	42466001	5700	1	8.51E-11	48	0.84		
DMR10:42598401	10	42598401	3400	1	1.60E-14	12	0.35		
DMR10:42664601	10	42664601	4200	2	1.53E-12	29	0.69	Gria1	Receptor
DMR10:43348801	10	43348801	4200	1	2.32E-10	77	1.83		
DMR10:44262901	10	44262901	6300	1	3.68E-11	114	1.8	Olr1432;Trim58	Receptor;Protease
DMR10:44664301	10	44664301	3400	3	1.12E-12	11	0.32	RGD1559534	Unknown
DMR10:45452901	10	45452901	5900	5	1.58E-12	97	1.64	Obscn	Unknown
DMR10:47709901	10	47709901	1200	2	2.44E-11	12	1	Rnf112	Signaling
DMR10:47760401	10	47760401	1500	1	1.46E-10	19	1.26	Mfap4;Mapk7	Development;Signaling
DMR10:47826301	10	47826301	1700	1	3.67E-13	6	0.35	Epn2	Transport
DMR10:54245301	10	54245301	3300	1	5.04E-10	49	1.48	Rcvrn;Gas7	Signaling;Transcription
DMR10:56974001	10	56974001	2900	1	5.04E-11	20	0.68		
DMR10:58037701	10	58037701	2400	1	2.24E-11	22	0.91		
DMR10:58047001	10	58047001	2800	1	2.41E-10	20	0.71		
DMR10:67205301	10	67205301	5200	2	2.25E-11	50	0.96		
DMR10:68470001	10	68470001	1400	1	2.30E-10	8	0.57	Asic2	Transport
DMR10:68614201	10	68614201	3700	1	1.77E-13	68	1.83		
DMR10:73417801	10	73417801	3600	1	8.17E-10	36	1		
DMR10:74611201	10	74611201	5100	1	5.58E-15	29	0.56	Ppm1e	Signaling
DMR10:76061001	10	76061001	200	1	7.39E-10	0	0		
DMR10:76641501	10	76641501	2800	1	2.66E-10	22	0.78	AABR07030221.1	Unknown
DMR10:77016801	10	77016801	1900	1	3.78E-12	27	1.42		
DMR10:77859601	10	77859601	3400	1	5.13E-10	64	1.88	Hlf	Transcription
DMR10:80617301	10	80617301	500	1	2.27E-11	1	0.2		
DMR10:81107501	10	81107501	500	1	6.65E-10	2	0.4	Car10	Metabolism
DMR10:82929901	10	82929901	2900	1	1.41E-13	39	1.34	Dlx3	Transcription
DMR10:83969501	10	83969501	1900	1	4.72E-13	33	1.73	Ttll6	Cytoskeleton
DMR10:85162001	10	85162001	900	1	1.93E-10	9	1	Npepps	Unknown
DMR10:87465401	10	87465401	2200	2	1.20E-11	18	0.81	Rn50_10_0873.4;Krtap3-3;I	Unknown;Cytoskeleton
DMR10:98019901	10	98019901	4300	1	6.10E-11	61	1.41	Fam20a	Unknown
DMR10:101600401	10	101600401	2200	1	1.14E-10	31	1.4		
DMR10:102125701	10	102125701	3000	1	7.63E-10	27	0.9	Sstr2	Receptor
DMR10:107053201	10	107053201	5800	1	7.21E-11	173	2.98	Dnah17	Cytoskeleton

DMR10:110015001	10	110015001	600	2	9.32E-13	4	0.66	Fasn;Ccdc57	Metabolism;Unknown
DMR11:933301	11	933301	2200	1	5.24E-10	16	0.72		
DMR11:5552201	11	5552201	500	1	7.69E-11	1	0.2		
DMR11:15660701	11	15660701	1300	1	1.57E-10	2	0.15	AABR07033285.1	Unknown
DMR11:16476301	11	16476301	2700	1	9.74E-11	15	0.55		
DMR11:16480401	11	16480401	9000	1	2.36E-10	86	0.95	AABR07033296.1	Unknown
DMR11:17215201	11	17215201	800	1	8.59E-14	11	1.37		
DMR11:17879001	11	17879001	3800	2	3.25E-14	17	0.44		
DMR11:17893501	11	17893501	1800	2	1.12E-13	7	0.38		
DMR11:26833501	11	26833501	1400	1	4.58E-12	12	0.85		
DMR11:27955501	11	27955501	900	1	1.14E-12	4	0.44	Grik1	Receptor;Transport
DMR11:35687601	11	35687601	4000	1	2.44E-10	37	0.92	Erg	Transcription
DMR11:45138201	11	45138201	300	1	5.55E-10	1	0.33		
DMR11:46266701	11	46266701	500	1	4.30E-10	0	0	Abi3bp	Unknown
DMR11:46454101	11	46454101	1800	1	1.03E-10	7	0.38		
DMR11:47344701	11	47344701	2300	1	1.16E-10	19	0.82		
DMR11:47462901	11	47462901	1200	1	5.87E-13	3	0.25		
DMR11:52039801	11	52039801	2900	2	3.43E-13	16	0.55		
DMR11:54307701	11	54307701	5400	2	9.51E-13	27	0.5	Myh15	Cytoskeleton
DMR11:54520901	11	54520901	2500	3	7.98E-11	16	0.64	Retnlg	Development
DMR11:55172101	11	55172101	3300	2	3.08E-11	21	0.63		
DMR11:56887301	11	56887301	1100	1	6.95E-13	10	0.9		
DMR11:60166601	11	60166601	4300	1	7.12E-10	38	0.88	LOC685680;Gcsam	Unknown
DMR11:63652301	11	63652301	1900	1	6.85E-10	6	0.31		
DMR11:64540801	11	64540801	400	1	1.01E-10	3	0.75	Upk1b	Signaling
DMR11:66016001	11	66016001	4700	1	4.24E-12	62	1.31	Hgd	Metabolism
DMR11:69267201	11	69267201	5700	1	6.35E-10	70	1.22	Ccdc14	Unknown
DMR11:69621201	11	69621201	1000	1	5.03E-10	14	1.4	Kalrn	Signaling
DMR11:70471101	11	70471101	2000	1	1.54E-10	23	1.15	Slc12a8	Transport
DMR11:74832201	11	74832201	1200	1	6.80E-10	9	0.75	Atp13a4	Transport
DMR11:75647001	11	75647001	2300	1	1.69E-10	22	0.95		
DMR11:79335501	11	79335501	2800	1	4.26E-11	43	1.53	Lpp	Cytoskeleton
DMR11:84795601	11	84795601	1200	5	2.59E-18	2	0.16	Klhl6	Unknown
DMR11:87467701	11	87467701	400	1	7.97E-10	1	0.25		
DMR12:1263101	12	1263101	7500	2	1.62E-10	118	1.57		
DMR12:2760101	12	2760101	7700	1	1.26E-10	46	0.59		
DMR12:7050201	12	7050201	1600	1	3.34E-11	23	1.43		
DMR12:8333901	12	8333901	6200	1	6.35E-10	76	1.22	Mtus2	Cytoskeleton
DMR12:10216301	12	10216301	8400	1	1.31E-10	101	1.2		
DMR12:10246001	12	10246001	1300	1	4.37E-10	5	0.38	Gpr12	Receptor
DMR12:11374701	12	11374701	900	1	9.44E-12	5	0.55	Kpna7	Unknown
DMR12:11886101	12	11886101	1600	1	4.81E-11	21	1.31		
DMR12:13400001	12	13400001	2200	1	4.18E-10	22	1	Spdye4	Cell Cycle
DMR12:20275901	12	20275901	1500	2	1.52E-11	14	0.93	LOC685157	Immune
DMR12:20380001	12	20380001	8000	1	1.48E-10	47	0.58		
DMR12:20653901	12	20653901	7100	3	1.48E-11	50	0.7	AABR07035641.1	Unknown
DMR12:20662001	12	20662001	12400	1	1.92E-10	59	0.47	AABR07035641.1	Unknown
DMR12:21090101	12	21090101	3900	1	1.72E-10	19	0.48		
DMR12:21156401	12	21156401	1500	1	4.24E-10	13	0.86		
DMR12:21425201	12	21425201	5000	1	7.72E-12	38	0.76	LOC100910636;AABR07035	Unknown
DMR12:21510401	12	21510401	2300	2	4.06E-14	10	0.43		
DMR12:21522201	12	21522201	6400	1	1.41E-10	62	0.96		
DMR12:21575601	12	21575601	4800	1	3.19E-10	26	0.54		
DMR12:21669301	12	21669301	3900	1	1.07E-10	24	0.61	LOC100910669	Immune
DMR12:21679401	12	21679401	9900	2	1.16E-11	140	1.41	LOC100910636	Unknown;Immune
DMR12:21926901	12	21926901	2700	3	7.47E-12	26	0.96	RGD1561143	Immune;Unknown
DMR12:22535601	12	22535601	7300	2	2.43E-12	94	1.28	AABR07035791.1	Unknown
DMR12:24716701	12	24716701	1700	2	7.52E-12	9	0.52	Abhd11;Stx1a	Metabolism;Transport
DMR12:25289101	12	25289101	1800	1	6.49E-10	16	0.88	5S_rRNA;Gtf2ird1	Unknown;Transcription
DMR12:26646801	12	26646801	3300	1	7.27E-10	21	0.63		
DMR12:28995901	12	28995901	1400	1	9.11E-12	8	0.57	5S_rRNA;Wbscr17	Unknown;Development
DMR12:29032501	12	29032501	2400	1	6.93E-13	33	1.37	LOC100912262;Wbscr17	Unknown;Development
DMR12:29528301	12	29528301	4400	1	4.69E-10	36	0.81	Caln1	Signaling
DMR12:31532401	12	31532401	500	1	4.59E-10	3	0.6	Rimbp2	Unknown
DMR12:36207401	12	36207401	1000	2	5.67E-11	11	1.1	Tmem132b	Unknown
DMR12:36419601	12	36419601	800	1	6.20E-11	13	1.62		
DMR12:37714001	12	37714001	4000	2	4.58E-12	41	1.02	Mphosph9	Cell Cycle

DMR12:42845601	12	42845601	2400	1	8.71E-12	21	0.87		
DMR12:43042101	12	43042101	4100	1	1.16E-11	64	1.56		
DMR12:43130001	12	43130001	1900	1	9.02E-11	24	1.26		
DMR12:43149501	12	43149501	6100	1	2.60E-10	95	1.55		
DMR12:43357201	12	43357201	1600	1	2.48E-10	19	1.18		
DMR12:43837801	12	43837801	2900	1	6.33E-11	54	1.86		
DMR12:49548201	12	49548201	3100	1	3.41E-10	44	1.41	AABR07036608.1	Unknown
DMR12:50780501	12	50780501	3400	2	1.41E-10	42	1.23		
DMR12:51086601	12	51086601	2300	1	2.90E-10	20	0.86		
DMR12:51221801	12	51221801	800	1	9.77E-14	4	0.5	Mn1	Unknown
DMR13:1874001	13	1874001	1300	1	1.24E-10	3	0.23	Rn60_13_0019.1	Unknown
DMR13:4719301	13	4719301	700	1	8.78E-10	0	0		
DMR13:11879901	13	11879901	700	1	1.97E-11	7	1		
DMR13:13668401	13	13668401	800	1	2.01E-11	4	0.5		
DMR13:15480501	13	15480501	1500	1	2.36E-10	18	1.2		
DMR13:18651301	13	18651301	800	1	3.08E-11	1	0.12	AABR07020222.1	Unknown
DMR13:19389701	13	19389701	5000	1	2.97E-10	28	0.56		
DMR13:19622801	13	19622801	4600	1	7.69E-10	28	0.6		
DMR13:19652301	13	19652301	2800	1	1.91E-12	8	0.28		
DMR13:20504701	13	20504701	600	1	8.70E-11	3	0.5		
DMR13:22583701	13	22583701	500	1	4.05E-11	0	0	Cntnap5b	Cytoskeleton
DMR13:26226901	13	26226901	3500	1	4.58E-10	37	1.05	Phlpp1	Signaling
DMR13:27070001	13	27070001	1800	1	8.73E-10	5	0.27	Serpinb13	Protease
DMR13:35540201	13	35540201	7700	1	2.17E-10	95	1.23	Tmem185b	Unknown
DMR13:37386901	13	37386901	900	1	3.97E-11	3	0.33		
DMR13:37967401	13	37967401	1600	1	1.82E-10	11	0.68		
DMR13:38058801	13	38058801	900	1	3.16E-10	5	0.55		
DMR13:38842001	13	38842001	2200	1	1.42E-10	8	0.36		
DMR13:42953101	13	42953101	3100	1	1.16E-11	35	1.12		
DMR13:44242901	13	44242901	3500	1	1.28E-10	39	1.11	Tmem163	Unknown
DMR13:46727401	13	46727401	2200	1	9.04E-10	11	0.5	Thsd7b	Extracellular Matrix
DMR13:48485401	13	48485401	2700	1	3.56E-10	24	0.88	Rab7b	Signaling
DMR13:49538201	13	49538201	1800	1	6.02E-12	20	1.11		
DMR13:50990401	13	50990401	2800	1	1.02E-10	22	0.78	Chit1	Metabolism
DMR13:59408501	13	59408501	800	1	4.67E-10	3	0.37		
DMR13:70663101	13	70663101	4800	1	2.32E-11	88	1.83	Lamc1	Extracellular Matrix
DMR13:73358701	13	73358701	5400	1	1.50E-10	68	1.25	Lhx4	Transcription
DMR13:86252301	13	86252301	2300	1	6.04E-11	9	0.39		
DMR13:87278301	13	87278301	700	1	6.20E-11	5	0.71		
DMR13:87589801	13	87589801	1300	1	2.02E-10	16	1.23		
DMR13:87750301	13	87750301	1700	1	2.22E-10	6	0.35		
DMR13:97421301	13	97421301	1100	2	8.77E-13	13	1.18		
DMR13:100679301	13	100679301	2800	1	5.15E-10	30	1.07	Degs1	Metabolism
DMR13:106743001	13	106743001	1800	1	9.58E-13	26	1.44	Ush2a	Extracellular Matrix
DMR13:108367501	13	108367501	4200	1	4.36E-10	55	1.3		
DMR13:110936101	13	110936101	4500	1	5.15E-11	42	0.93	Kcnh1	Transport
DMR14:3984201	14	3984201	2100	1	1.56E-11	16	0.76	AABR07014161.2	Unknown
DMR14:4498401	14	4498401	500	1	6.93E-10	5	1		
DMR14:7286001	14	7286001	6600	1	2.05E-17	153	2.31	Aff1	Transcription
DMR14:7812401	14	7812401	1700	1	7.49E-10	57	3.35	Ptpn13	Signaling
DMR14:7870601	14	7870601	11200	1	4.03E-10	151	1.34	Ptpn13	Signaling
DMR14:8869201	14	8869201	3900	1	2.48E-10	48	1.23		
DMR14:9077201	14	9077201	1800	1	6.76E-10	17	0.94	Rn60_14_0094.2	Unknown
DMR14:9093601	14	9093601	4200	1	5.74E-13	54	1.28	AABR07014317.1;Rn60_14_	Unknown
DMR14:10291701	14	10291701	500	1	2.01E-10	1	0.2	AABR07014342.1	Unknown
DMR14:11057201	14	11057201	900	1	5.00E-10	17	1.88		
DMR14:13798301	14	13798301	700	1	4.79E-10	9	1.28		
DMR14:15282701	14	15282701	700	1	5.26E-10	5	0.71		
DMR14:17179101	14	17179101	300	1	2.16E-10	2	0.66	Art3	Metabolism
DMR14:23087601	14	23087601	2000	1	4.75E-11	16	0.8	Tmprss11e	Protease
DMR14:27061101	14	27061101	2100	1	2.05E-10	13	0.61		
DMR14:29309901	14	29309901	500	1	1.28E-10	3	0.6		
DMR14:29817701	14	29817701	3300	1	1.13E-11	9	0.27		
DMR14:30447901	14	30447901	1100	1	2.15E-11	4	0.36		
DMR14:30981301	14	30981301	4100	1	3.82E-10	19	0.46		
DMR14:31303801	14	31303801	900	1	8.77E-11	8	0.88		
DMR14:33197301	14	33197301	22100	8	4.11E-16	172	0.77		

DMR14:33227501	14	33227501	8100	2	2.13E-15	42	0.51	AC097433.1	Unknown
DMR14:33321801	14	33321801	700	1	1.18E-11	3	0.42		
DMR14:33697901	14	33697901	1800	1	2.11E-11	23	1.27		
DMR14:33876101	14	33876101	3500	1	7.07E-10	18	0.51		
DMR14:35420401	14	35420401	2100	1	2.02E-10	27	1.28		
DMR14:35800301	14	35800301	8200	1	4.37E-13	110	1.34	AABR07014885.1	Unknown
DMR14:43582001	14	43582001	2000	1	1.00E-10	33	1.65	Nsun7;AABR07014992.2	Epigenetic;Unknown
DMR14:58904601	14	58904601	3900	1	2.09E-12	26	0.66		
DMR14:70641301	14	70641301	800	1	1.25E-11	2	0.25		
DMR14:72591801	14	72591801	400	1	6.52E-11	2	0.5		
DMR14:76276401	14	76276401	3500	1	6.56E-12	13	0.37	AABR07015784.1	Unknown
DMR14:79712601	14	79712601	9100	1	3.11E-12	140	1.53	Sorcs2;Psapl1	Receptor;Unknown
DMR14:87379001	14	87379001	3400	1	9.18E-11	20	0.58	Adcy1	Signaling
DMR14:98653201	14	98653201	4300	1	2.03E-11	101	2.34		
DMR14:100694601	14	100694601	700	1	1.97E-11	0	0		
DMR14:108604601	14	108604601	1700	1	8.04E-10	8	0.47		
DMR14:114019101	14	114019101	2100	1	3.06E-10	3	0.14	AABR07016779.1	Unknown
DMR14:114223401	14	114223401	1600	1	1.36E-10	19	1.18	Eml6	Cytoskeleton
DMR15:4455401	15	4455401	4900	1	4.28E-10	65	1.32	Nudt13	Metabolism
DMR15:6456901	15	6456901	1200	1	2.82E-11	7	0.58	Zfp385d	Transcription
DMR15:16200601	15	16200601	2000	1	1.48E-10	8	0.4		
DMR15:16959001	15	16959001	2100	1	6.93E-10	10	0.47		
DMR15:18356401	15	18356401	3800	1	2.71E-13	42	1.1	Fam3d	Unknown
DMR15:20396301	15	20396301	3700	1	4.73E-11	55	1.48		
DMR15:21022101	15	21022101	2000	1	4.70E-12	29	1.45		
DMR15:35239401	15	35239401	2700	1	6.51E-11	13	0.48		
DMR15:41210701	15	41210701	1900	1	2.94E-11	17	0.89		
DMR15:43132901	15	43132901	2000	1	3.65E-10	13	0.65		
DMR15:49115001	15	49115001	2700	1	2.09E-13	35	1.29	Scara5	Unknown
DMR15:50337401	15	50337401	2800	2	2.43E-10	7	0.25		
DMR15:60656801	15	60656801	1000	1	1.63E-10	7	0.7		
DMR15:61490501	15	61490501	3900	4	1.73E-13	51	1.3		
DMR15:72023501	15	72023501	400	1	1.88E-12	1	0.25		
DMR15:74996001	15	74996001	1400	1	2.03E-10	2	0.14		
DMR15:79375901	15	79375901	700	1	5.10E-10	5	0.71		
DMR15:82294301	15	82294301	400	1	5.96E-10	3	0.75	Dach1	Transcription
DMR15:86553701	15	86553701	1800	1	2.71E-10	6	0.33		
DMR15:89046301	15	89046301	4000	1	5.74E-10	54	1.35		
DMR15:89235701	15	89235701	600	1	7.22E-10	3	0.5		
DMR15:96741401	15	96741401	1100	1	7.17E-10	7	0.63		
DMR15:96757201	15	96757201	3100	1	8.60E-11	15	0.48		
DMR15:97033901	15	97033901	2500	1	9.71E-11	10	0.4		
DMR15:98135001	15	98135001	900	1	5.86E-13	6	0.66		
DMR15:104017201	15	104017201	2200	1	6.29E-10	31	1.4	AABR07019398.1;Cldn10	Unknown;Cell Junction
DMR15:104048801	15	104048801	2000	1	5.93E-10	25	1.25	Cldn10	Cell Junction
DMR15:104697901	15	104697901	3900	1	3.01E-10	40	1.02		
DMR15:104895201	15	104895201	2900	1	6.84E-10	30	1.03		
DMR15:105595601	15	105595601	2500	1	1.32E-12	31	1.24		
DMR15:106198701	15	106198701	7400	1	2.82E-10	114	1.54	AABR07019449.1	Unknown
DMR15:106303101	15	106303101	4400	1	7.76E-11	46	1.04		
DMR15:106498001	15	106498001	26300	2	3.30E-10	438	1.66	Farp1	Signaling
DMR15:106578001	15	106578001	700	1	1.44E-11	6	0.85	Farp1	Signaling
DMR15:108479901	15	108479901	900	2	2.00E-11	2	0.22		
DMR16:5497501	16	5497501	1100	1	1.16E-11	4	0.36	Cacna2d3	Transport
DMR16:12589601	16	12589601	2700	1	2.19E-10	15	0.55	AABR07024721.1	Unknown
DMR16:12702301	16	12702301	3300	1	3.37E-10	10	0.3	AABR07024724.1	Unknown
DMR16:17423701	16	17423701	1300	2	1.06E-11	12	0.92		
DMR16:18368901	16	18368901	2500	1	9.02E-10	11	0.44	AABR07024825.1	Unknown
DMR16:18694401	16	18694401	2200	1	7.35E-10	19	0.86	Mat1a	Metabolism
DMR16:25975301	16	25975301	3800	7	1.73E-13	11	0.28		
DMR16:28955001	16	28955001	2500	1	2.52E-12	3	0.12		
DMR16:40925201	16	40925201	2000	1	5.25E-13	1	0.05		
DMR16:50595501	16	50595501	2400	1	1.84E-10	20	0.83		
DMR16:50847701	16	50847701	300	1	1.47E-11	3	1	5S_rRNA	Unknown
DMR16:57042301	16	57042301	2100	1	6.95E-11	12	0.57		
DMR16:60445101	16	60445101	5600	1	8.31E-15	54	0.96		
DMR16:65256601	16	65256601	1500	1	9.89E-10	9	0.6	AABR07026137.2	Unknown

DMR16:72249201	16	72249201	2100	1	2.14E-10	17	0.8		
DMR16:72393001	16	72393001	1900	1	7.33E-10	23	1.21	LOC100910163	Unknown
DMR16:74611401	16	74611401	2600	1	2.00E-10	30	1.15	Tpte2	Signaling
DMR16:75176601	16	75176601	1100	1	1.77E-10	1	0.09	Defb9	Unknown
DMR16:75574301	16	75574301	4300	1	4.76E-11	35	0.81	Defb52;Defb33	Unknown
DMR16:77880901	16	77880901	2100	1	4.64E-12	19	0.9		
DMR16:78538001	16	78538001	3500	1	5.34E-10	32	0.91	Csmd1	Signaling
DMR16:78795301	16	78795301	900	1	1.36E-10	9	1	Csmd1	Signaling
DMR16:78865001	16	78865001	4500	1	2.45E-11	17	0.37		
DMR16:78883401	16	78883401	2800	1	4.38E-10	23	0.82	AABR07026492.1	Unknown
DMR16:79095701	16	79095701	2000	3	8.00E-18	14	0.7		
DMR16:81256001	16	81256001	1500	1	3.02E-10	4	0.26	AABR07026534.2	Unknown
DMR16:82903501	16	82903501	4500	1	2.70E-10	56	1.24		
DMR16:84000701	16	84000701	5200	2	2.20E-10	49	0.942		
DMR16:86324701	16	86324701	3700	1	3.92E-12	37	1		
DMR16:87904401	16	87904401	2100	1	5.00E-16	6	0.28		
DMR16:89306401	16	89306401	5400	1	5.22E-10	46	0.85		
DMR17:3992601	17	3992601	1700	1	8.03E-11	9	0.52	Ctsq	Protease
DMR17:4056401	17	4056401	500	1	6.73E-10	0	0	LOC100364523;Cts8l1	Unknown;Protease
DMR17:4717301	17	4717301	1400	1	1.37E-10	12	0.85		
DMR17:5065101	17	5065101	4200	1	4.79E-11	65	1.54		
DMR17:5178201	17	5178201	2900	1	1.16E-14	39	1.34		
DMR17:6015401	17	6015401	5500	1	1.16E-12	99	1.8	Ntrk2	Receptor
DMR17:10908901	17	10908901	6500	1	6.88E-10	86	1.32	Hrh2	Receptor
DMR17:16614301	17	16614301	2900	1	6.87E-12	38	1.31		
DMR17:17194101	17	17194101	2900	1	8.60E-11	36	1.24		
DMR17:18122301	17	18122301	4800	1	6.17E-11	68	1.41		
DMR17:20298601	17	20298601	2500	1	4.16E-10	24	0.96	Jarid2	Epigenetic
DMR17:20789001	17	20789001	2500	1	5.83E-11	25	1		
DMR17:22449001	17	22449001	1400	1	1.09E-10	4	0.28		
DMR17:27856001	17	27856001	4700	2	7.56E-10	61	1.29	AABR07027390.1	Unknown
DMR17:36317401	17	36317401	3800	1	2.96E-15	29	0.76		
DMR17:40033401	17	40033401	1900	3	1.02E-13	16	0.84		
DMR17:40154801	17	40154801	2100	1	1.71E-13	8	0.38		
DMR17:42859601	17	42859601	700	3	4.84E-13	7	1	Prl3d1	Hormone
DMR17:43541801	17	43541801	3000	1	1.75E-10	15	0.5	Slc17a3	Transport
DMR17:51612701	17	51612701	1400	1	4.77E-14	8	0.57		
DMR17:58684401	17	58684401	1800	1	2.29E-10	6	0.33	AABR07028172.1	Unknown
DMR17:62275201	17	62275201	1400	1	1.52E-10	7	0.5		
DMR17:63960601	17	63960601	1700	1	1.79E-12	10	0.58		
DMR17:65520401	17	65520401	1900	1	2.06E-10	18	0.94	AABR07028327.1	Unknown
DMR17:70470501	17	70470501	6100	1	3.98E-10	82	1.34	Il15ra	Immune
DMR17:70841101	17	70841101	1900	2	4.32E-15	24	1.26		
DMR17:73241301	17	73241301	4000	1	5.60E-10	22	0.55		
DMR17:75181601	17	75181601	600	1	3.96E-11	5	0.83	AABR07028568.1	Unknown
DMR17:75901101	17	75901101	1300	1	3.26E-10	7	0.53		
DMR17:78031601	17	78031601	4800	1	7.51E-10	40	0.83		
DMR17:78379101	17	78379101	3400	3	3.03E-10	20	0.58		
DMR17:80023601	17	80023601	2700	1	8.27E-10	22	0.81		
DMR17:81585101	17	81585101	6400	1	3.40E-10	43	0.67		
DMR17:81768401	17	81768401	400	1	8.79E-10	5	1.25		
DMR17:83510001	17	83510001	1600	1	5.98E-10	13	0.81	Plxdc2	Binding Protein
DMR17:87309701	17	87309701	1500	1	9.03E-10	1	0.06	Etl4	Unknown
DMR17:88263801	17	88263801	600	1	7.05E-10	4	0.66	Gpr158	Receptor
DMR17:88898301	17	88898301	4700	1	4.37E-10	18	0.38		
DMR17:88912001	17	88912001	700	1	5.97E-10	4	0.57		
DMR18:1424501	18	1424501	3700	1	3.37E-10	14	0.37		
DMR18:5831501	18	5831501	1900	1	1.20E-10	7	0.36	AABR07031251.1	Unknown
DMR18:7621301	18	7621301	600	1	4.21E-11	2	0.33		
DMR18:16622301	18	16622301	2900	1	8.36E-12	36	1.24	5S_rRNA;Mocos	Unknown;Metabolism
DMR18:18179701	18	18179701	1200	1	2.36E-11	3	0.25		
DMR18:27323901	18	27323901	1400	1	2.89E-11	15	1.07	Wnt8a	Signaling
DMR18:27825501	18	27825501	900	3	6.40E-12	1	0.11		
DMR18:32986301	18	32986301	1200	1	6.54E-10	7	0.58	AABR07031759.1	Unknown
DMR18:37004901	18	37004901	500	1	1.85E-11	3	0.6	Ppp2r2b	Signaling
DMR18:41754601	18	41754601	5800	1	1.34E-10	38	0.65		
DMR18:42048901	18	42048901	600	1	5.56E-10	2	0.33		

DMR18:45126401	18	45126401	2200	1	3.19E-11	3	0.13		
DMR18:49621501	18	49621501	2600	1	5.72E-11	22	0.84		
DMR18:50615801	18	50615801	2000	1	1.06E-11	14	0.7		
DMR18:52226901	18	52226901	1900	1	7.52E-13	16	0.84	Megf10	Extracellular Matrix
DMR18:52237901	18	52237901	3200	2	1.16E-10	33	1.03	Megf10	Extracellular Matrix
DMR18:57175101	18	57175101	4300	1	3.85E-12	40	0.93	Ablim3	Cytoskeleton
DMR18:62271801	18	62271801	3900	1	3.04E-10	30	0.76	AABR07032348.2	Unknown
DMR18:63991001	18	63991001	2200	1	1.69E-13	20	0.9	Ldlrad4	Receptor
DMR18:65979201	18	65979201	1700	2	8.01E-12	13	0.76		
DMR18:66013601	18	66013601	2700	1	2.74E-10	24	0.88		
DMR18:68972001	18	68972001	1800	1	6.00E-10	13	0.72	Stard6;LOC361346	Transport;Unknown
DMR18:73841501	18	73841501	1800	1	1.53E-14	19	1.05	Rnf165	Development
DMR18:77220501	18	77220501	2000	1	1.47E-10	22	1.1	Nfatc1	Transcription
DMR18:79198001	18	79198001	2800	1	4.04E-10	19	0.67		
DMR18:79619701	18	79619701	1300	2	1.05E-10	6	0.46		
DMR18:79804201	18	79804201	1200	1	2.66E-14	8	0.66	Zfp516	Transcription
DMR18:80180901	18	80180901	3700	2	2.70E-10	51	1.37	AABR07032757.1	Unknown
DMR18:81012701	18	81012701	6400	1	5.47E-11	158	2.46		
DMR18:81337801	18	81337801	1300	1	2.53E-11	21	1.61	Zfp407	Transcription
DMR18:81438801	18	81438801	2700	2	1.73E-14	42	1.55	Zfp407	Transcription
DMR18:81495401	18	81495401	2200	1	9.32E-11	14	0.63	Cndp1	Protease
DMR18:81933901	18	81933901	300	1	8.80E-10	4	1.33		
DMR18:86366401	18	86366401	700	1	6.07E-11	0	0	Cd226	Immune
DMR19:453701	19	453701	1800	1	2.39E-11	19	1.05	AABR07042633.1	Unknown
DMR19:8750001	19	8750001	2500	1	7.23E-12	14	0.56		
DMR19:9375701	19	9375701	900	1	1.23E-10	2	0.22		
DMR19:14725101	19	14725101	9500	2	2.77E-17	171	1.8		
DMR19:15828701	19	15828701	800	1	2.43E-10	4	0.5	Irx3	Transcription
DMR19:29149001	19	29149001	5600	2	7.92E-13	42	0.75	AABR07043525.1	Unknown
DMR19:29861301	19	29861301	2900	2	1.35E-16	29	1	Inpp4b	Signaling
DMR19:31752501	19	31752501	700	1	3.68E-10	2	0.28		
DMR19:35738101	19	35738101	2600	1	9.18E-10	19	0.73		
DMR19:36657001	19	36657001	1700	1	8.19E-10	12	0.7		
DMR19:39977201	19	39977201	1100	1	5.06E-10	9	0.81		
DMR19:40671401	19	40671401	2900	1	9.32E-15	27	0.93		
DMR19:44348401	19	44348401	6900	1	8.08E-10	50	0.72		
DMR19:44530401	19	44530401	300	1	8.72E-10	1	0.33		
DMR19:44642301	19	44642301	2200	1	5.07E-10	9	0.4		
DMR19:44921801	19	44921801	1300	1	2.53E-10	15	1.15	Cntnap4	Signaling
DMR19:44975301	19	44975301	1500	1	3.88E-10	6	0.4	Cntnap4	Signaling
DMR19:45820601	19	45820601	500	1	4.65E-14	4	0.8	AABR07043881.1	Unknown
DMR19:47145201	19	47145201	1500	1	1.67E-10	10	0.66		
DMR19:49193401	19	49193401	1200	1	1.31E-10	9	0.75	Cdyl2	Metabolism
DMR19:49350701	19	49350701	2600	1	2.56E-10	16	0.61	Cenpn	Cell Cycle
DMR19:50255101	19	50255101	4100	1	2.62E-10	41	1	Hsd17b2	Metabolism
DMR19:50418201	19	50418201	1500	2	3.92E-10	17	1.13		
DMR19:51756501	19	51756501	2000	1	5.13E-12	13	0.65	Cdh13	Extracellular Matrix
DMR19:52419201	19	52419201	3300	1	8.48E-10	48	1.45	Tldc1	Unknown
DMR19:52857101	19	52857101	6800	1	5.03E-10	109	1.6		
DMR19:53226601	19	53226601	1300	1	8.92E-12	20	1.53		
DMR19:56880901	19	56880901	3500	1	4.30E-11	44	1.25	AABR07072664.1	Unknown
DMR19:60001001	19	60001001	2300	1	8.27E-10	24	1.04	AABR07072668.1	Unknown
DMR20:831701	20	831701	1900	2	4.39E-10	17	0.89	Olr1693	Receptor
DMR20:2586401	20	2586401	600	1	1.10E-12	4	0.66	Rn50_20_0026.4	Unknown
DMR20:2985301	20	2985301	4200	1	1.74E-13	27	0.64	AABR07044346.1;Rn60_20_	Unknown
DMR20:3140401	20	3140401	16900	1	3.02E-12	171	1.01	AABR07044362.1;Rps2-ps2;	Unknown;Immune
DMR20:3256501	20	3256501	1200	1	6.74E-10	23	1.91	RT1-T24-1;LOC100909797	Immune
DMR20:3259101	20	3259101	7800	2	5.44E-12	127	1.62	RT1-T24-1;LOC100909797	Immune
DMR20:3985401	20	3985401	2900	1	2.36E-10	45	1.55	Psmb8;Tap1;Tap2;Psmb9	Protease;Transport
DMR20:8302401	20	8302401	3600	1	6.60E-11	111	3.08	Cmtr1;Rnf8	Epigenetic;Proteolysis
DMR20:8657701	20	8657701	3700	1	4.53E-12	56	1.51		
DMR20:15127901	20	15127901	1500	1	1.29E-11	10	0.66	Pcdh15	Extracellular Matrix
DMR20:20315101	20	20315101	3000	1	9.81E-10	49	1.63	Ank3	Cytoskeleton
DMR20:20744601	20	20744601	1200	1	5.43E-13	15	1.25		
DMR20:22371101	20	22371101	2800	1	4.58E-10	34	1.21		
DMR20:22560501	20	22560501	9000	2	1.29E-11	101	1.12		
DMR20:26989601	20	26989601	7800	1	2.99E-10	74	0.94	Mypn	Cytoskeleton

DMR20:28475001	20	28475001	5300	1	2.40E-11	42	0.79		
DMR20:31815501	20	31815501	1500	1	6.16E-11	17	1.13	Tspan15	Extracellular Matrix
DMR20:34611101	20	34611101	6000	2	1.73E-10	33	0.55	Cep85l	Epigenetic
DMR20:44392201	20	44392201	4600	1	2.01E-10	51	1.1		
DMR20:49876901	20	49876901	8300	1	6.56E-10	72	0.86	AABR07045510.1	Unknown
DMR20:51699601	20	51699601	800	1	2.38E-10	3	0.37		
DMR20:52771301	20	52771301	2100	2	2.10E-12	4	0.19		
DMRX:85101	X	85101	4800	1	4.25E-14	24	0.5		
DMRX:29393601	X	29393601	1400	1	2.99E-12	9	0.64		
DMRX:58894601	X	58894601	400	1	4.05E-11	0	0		
DMRX:73587501	X	73587501	500	1	1.87E-10	7	1.4	Rn50_X_0744.1	Unknown
DMRX:84403301	X	84403301	7400	1	8.72E-11	26	0.35		
DMRX:107611501	X	107611501	600	1	4.51E-10	6	1	Tmsbl1	Unknown
DMRX:117471201	X	117471201	3700	1	7.77E-10	26	0.7		
DMRX:119356601	X	119356601	800	1	5.26E-10	6	0.75		
DMRX:141074601	X	141074601	2000	1	1.37E-11	17	0.85		
DMRX:151484401	X	151484401	200	2	1.69E-12	1	0.5		
DMRX:159926301	X	159926301	5600	1	8.79E-10	36	0.64		
DMRY:18701	Y	18701	5900	1	9.96E-11	57	0.96	Rn60_Y_0004.4;Rn60_Y_00	Unknown
DMRY:273901	Y	273901	6600	1	1.92E-11	58	0.87	Rn60_Y_0001.3	Unknown
DMRY:412701	Y	412701	2900	1	2.23E-10	21	0.72	Rn60_Y_0008.1;Uba1y	Unknown;Proteolysis
DMRY:537201	Y	537201	5000	1	1.14E-10	19	0.38	Kdm5d;Rn60_Y_0006.2	Epigenetic;Unknown
DMRY:688601	Y	688601	3800	3	8.00E-11	28	0.73		
DMRY:2377601	Y	2377601	2000	1	6.02E-11	8	0.4		
