# Supplementary material for: Atrazine induced epigenetic transgenerational inheritance of disease, lean phenotype and sperm epimutation pathology biomarkers
Source: PLoS One. 2017 Sep 20;12(9):e0184306. doi: 10.1371/journal.pone.0184306 (PMC5606923; doi:10.1371/journal.pone.0184306)
Supplement: S6 Table — The overlapping testis disease DMR name, chromosome, lean and testis disease start site, lean and testis disease length (bp), lean and testis disease minimum p-value, lean and testis disease CpG number and density, and DMR associated gene are presented. (PDF) [file pone.0184306.s012.pdf]

**Supplemental Table S6**
**Overlap Between the Lean and Testis Disease Epimutation Signature**

| Overlap DMR.name | Chr | Lean Start | Testes Start | Lean Length | Testes Length | Lean MinP | Testes MinP | Lean CpG Num | Testes CpG Num | Lean CpG Density | Testes CpG Density | Annotation |
|---|---|---|---|---|---|---|---|---|---|---|---|---|
| oDMR:1 | 1 | 52818201 | 52818301 | 300 | 2400 | 1.28E-06 | 4.10E-06 | 1 | 24 | 0.33 | 1 | |
| oDMR:2 | 1 | 80882201 | 80881201 | 1100 | 1300 | 2.23E-06 | 1.21E-07 | 8 | 20 | 0.73 | 1.54 | Ceacam20 |
| oDMR:3 | 1 | 95955901 | 95954001 | 300 | 2500 | 3.78E-06 | 1.56E-06 | 4 | 21 | 1.33 | 0.84 | |
| oDMR:4 | 1 | 163524001 | 163523801 | 300 | 800 | 2.15E-07 | 1.88E-06 | 3 | 8 | 1 | 1 | AABR07004868.2 |
| oDMR:5 | 1 | 185926301 | 185926301 | 300 | 300 | 1.95E-08 | 5.18E-07 | 1 | 1 | 0.33 | 0.33 | Sox6 |
| oDMR:6 | 1 | 191605301 | 191604701 | 200 | 1200 | 4.29E-06 | 5.86E-06 | 4 | 10 | 2 | 0.83 | Usp31 |
| oDMR:7 | 1 | 195531601 | 195532101 | 700 | 100 | 1.77E-06 | 3.79E-07 | 2 | 0 | 0.29 | 0 | |
| oDMR:8 | 1 | 200251601 | 200251601 | 1900 | 1300 | 5.39E-06 | 2.49E-06 | 18 | 13 | 0.95 | 1 | |
| oDMR:9 | 1 | 205145201 | 205145101 | 1100 | 1200 | 4.70E-06 | 1.22E-06 | 7 | 8 | 0.64 | 0.67 | |
| oDMR:10 | 1 | 215134801 | 215134201 | 200 | 800 | 1.79E-06 | 9.81E-06 | 4 | 13 | 2 | 1.62 | LOC102557415; |
| oDMR:11 | 1 | 265841901 | 265842001 | 300 | 800 | 2.58E-06 | 9.02E-06 | 7 | 15 | 2.33 | 1.88 | Nolc1 |
| oDMR:12 | 2 | 66419301 | 66417601 | 1700 | 3500 | 1.16E-06 | 2.82E-06 | 12 | 21 | 0.71 | 0.6 | |
| oDMR:13 | 2 | 95359801 | 95360101 | 1200 | 900 | 4.43E-06 | 6.52E-07 | 18 | 13 | 1.5 | 1.44 | |
| oDMR:14 | 2 | 116691001 | 116691001 | 300 | 900 | 2.87E-08 | 2.36E-08 | 6 | 15 | 2 | 1.67 | |
| oDMR:15 | 2 | 152287101 | 152286901 | 1200 | 1300 | 4.01E-06 | 7.84E-06 | 2 | 3 | 0.17 | 0.23 | |
| oDMR:16 | 2 | 210988801 | 210989201 | 600 | 500 | 5.00E-06 | 4.21E-07 | 4 | 7 | 0.67 | 1.4 | Cyb561d1;Amigo1;Atxn7l2 |
| oDMR:17 | 2 | 251998101 | 251997901 | 2100 | 2300 | 7.22E-06 | 3.72E-06 | 19 | 20 | 0.9 | 0.87 | Mcoln3 |
| oDMR:18 | 3 | 54013101 | 54012601 | 200 | 2100 | 3.65E-06 | 6.81E-07 | 3 | 17 | 1.5 | 0.81 | |
| oDMR:19 | 3 | 67514901 | 67514001 | 1800 | 1500 | 6.69E-06 | 5.89E-06 | 25 | 18 | 1.39 | 1.2 | |
| oDMR:20 | 3 | 96291401 | 96289701 | 300 | 2000 | 1.31E-06 | 2.82E-07 | 2 | 9 | 0.67 | 0.45 | |
| oDMR:21 | 3 | 131490001 | 131487901 | 900 | 3000 | 6.93E-08 | 9.74E-07 | 12 | 26 | 1.33 | 0.87 | |
| oDMR:22 | 3 | 134918101 | 134915901 | 800 | 3100 | 6.06E-06 | 1.11E-06 | 9 | 24 | 1.12 | 0.77 | |
| oDMR:23 | 3 | 162667001 | 162667001 | 2700 | 2700 | 9.40E-07 | 6.35E-06 | 33 | 33 | 1.22 | 1.22 | |
| oDMR:24 | 3 | 166250901 | 166250901 | 200 | 700 | 3.98E-07 | 2.95E-08 | 3 | 21 | 1.5 | 3 | |
| oDMR:25 | 3 | 168075701 | 168075701 | 1300 | 1200 | 9.57E-06 | 3.47E-06 | 15 | 15 | 1.15 | 1.25 | |
| oDMR:26 | 3 | 174635601 | 174635701 | 900 | 800 | 1.66E-06 | 5.02E-06 | 6 | 6 | 0.67 | 0.75 | |
| oDMR:27 | 4 | 24048201 | 24048301 | 1100 | 1000 | 3.58E-06 | 1.97E-07 | 10 | 9 | 0.91 | 0.9 | |
| oDMR:28 | 4 | 43021901 | 43021201 | 600 | 1400 | 1.08E-06 | 3.21E-08 | 12 | 23 | 2 | 1.64 | Cttnbp2 |
| oDMR:29 | 4 | 55421501 | 55421101 | 200 | 1500 | 3.64E-06 | 4.08E-10 | 2 | 8 | 1 | 0.53 | |
| oDMR:30 | 4 | 62823301 | 62825001 | 1900 | 200 | 4.44E-07 | 6.09E-06 | 18 | 3 | 0.95 | 1.5 | Slc13a4 |
| oDMR:31 | 4 | 80723001 | 80721801 | 200 | 1500 | 8.91E-06 | 1.50E-06 | 6 | 31 | 3 | 2.07 | |
| oDMR:32 | 4 | 177821601 | 177820301 | 2900 | 3700 | 2.49E-06 | 1.66E-06 | 41 | 45 | 1.41 | 1.22 | |
| oDMR:33 | 4 | 177964301 | 177962201 | 1300 | 2500 | 6.95E-06 | 2.05E-07 | 24 | 29 | 1.85 | 1.16 | |
| oDMR:34 | 5 | 5704601 | 5704701 | 700 | 1000 | 5.54E-06 | 1.87E-06 | 13 | 18 | 1.86 | 1.8 | Prdm14 |
| oDMR:35 | 5 | 148610001 | 148610001 | 800 | 700 | 1.42E-06 | 3.23E-06 | 12 | 9 | 1.5 | 1.29 | Snrnp40 |
| oDMR:36 | 6 | 7417101 | 7417101 | 600 | 300 | 3.71E-06 | 4.48E-06 | 13 | 7 | 2.17 | 2.33 | Zfp36l2;Thada |
| oDMR:37 | 6 | 138197101 | 138196101 | 1500 | 5700 | 7.66E-06 | 1.58E-06 | 18 | 37 | 1.2 | 0.65 | Ighm |
| oDMR:38 | 6 | 141073601 | 141073601 | 100 | 1000 | 1.86E-06 | 4.46E-06 | 0 | 7 | 0 | 0.7 | |
| oDMR:39 | 8 | 3036401 | 3036701 | 2500 | 1300 | 2.57E-07 | 1.04E-06 | 13 | 9 | 0.52 | 0.69 | |
| oDMR:40 | 8 | 5557901 | 5558201 | 400 | 200 | 4.76E-07 | 1.37E-06 | 1 | 2 | 0.25 | 1 | |
| oDMR:41 | 8 | 45363701 | 45363201 | 2000 | 1100 | 7.40E-07 | 3.95E-09 | 29 | 13 | 1.45 | 1.18 | Ubash3b |
| oDMR:42 | 8 | 47709801 | 47709801 | 2400 | 6200 | 3.38E-06 | 6.16E-09 | 17 | 85 | 0.71 | 1.37 | |
| oDMR:43 | 8 | 64889101 | 64889101 | 1400 | 1400 | 5.41E-06 | 6.19E-06 | 24 | 24 | 1.71 | 1.71 | Rn50_8_0646.1 |
| oDMR:44 | 8 | 73512401 | 73512401 | 3100 | 2400 | 1.05E-06 | 3.80E-06 | 22 | 18 | 0.71 | 0.75 | |
| oDMR:45 | 8 | 90527301 | 90527301 | 300 | 300 | 9.13E-06 | 2.54E-06 | 4 | 4 | 1.33 | 1.33 | |
| oDMR:46 | 8 | 91827301 | 91827301 | 200 | 600 | 1.18E-06 | 7.25E-06 | 2 | 8 | 1 | 1.33 | RGD1560917 |
| oDMR:47 | 9 | 54645601 | 54645601 | 500 | 1000 | 2.00E-06 | 2.84E-06 | 1 | 2 | 0.2 | 0.2 | Myo1b |
| oDMR:48 | 9 | 110408201 | 110407601 | 3900 | 2500 | 4.88E-06 | 1.04E-06 | 55 | 32 | 1.41 | 1.28 | AABR07068590.1 |
| oDMR:49 | 10 | 8040401 | 8040401 | 2100 | 2100 | 5.28E-06 | 5.39E-06 | 37 | 37 | 1.76 | 1.76 | |
| oDMR:50 | 10 | 19719201 | 19719201 | 1100 | 300 | 2.66E-07 | 4.18E-06 | 18 | 6 | 1.64 | 2 | RGD1564698 |
| oDMR:51 | 10 | 31137201 | 31137201 | 300 | 500 | 7.77E-06 | 4.95E-06 | 5 | 5 | 1.67 | 1 | Adam19 |
| oDMR:52 | 10 | 98592901 | 98592801 | 700 | 300 | 1.74E-06 | 2.98E-06 | 6 | 1 | 0.86 | 0.33 | Abca5 |
| oDMR:53 | 11 | 63567501 | 63566301 | 300 | 1400 | 4.07E-06 | 5.37E-06 | 1 | 3 | 0.33 | 0.21 | |
| oDMR:54 | 11 | 66030801 | 66030501 | 1700 | 1200 | 6.94E-06 | 1.80E-06 | 17 | 8 | 1 | 0.67 | Hgd |
| oDMR:55 | 11 | 70738901 | 70737301 | 300 | 1800 | 8.41E-07 | 3.27E-06 | 1 | 10 | 0.33 | 0.56 | Snx4 |
| oDMR:56 | 12 | 8301801 | 8301801 | 1000 | 1900 | 1.24E-06 | 4.77E-06 | 21 | 38 | 2.1 | 2 | Mtus2 |
| oDMR:57 | 12 | 38367001 | 38365801 | 300 | 3000 | 7.83E-08 | 3.87E-08 | 4 | 40 | 1.33 | 1.33 | Clip1 |
| oDMR:58 | 12 | 38654001 | 38654001 | 1500 | 2100 | 1.89E-06 | 1.75E-06 | 32 | 50 | 2.13 | 2.38 | Bcl7a;AABR07072294.1 |
| oDMR:59 | 12 | 43732501 | 43735301 | 4100 | 600 | 1.87E-06 | 3.37E-06 | 42 | 5 | 1.02 | 0.83 | |
| oDMR:60 | 13 | 51743401 | 51743201 | 800 | 1000 | 9.81E-06 | 5.52E-06 | 7 | 8 | 0.88 | 0.8 | Ppp1r12b |
| oDMR:61 | 13 | 77113201 | 77113001 | 100 | 300 | 1.45E-06 | 3.43E-06 | 0 | 0 | 0 | 0 | |
| oDMR:62 | 14 | 32769401 | 32769001 | 300 | 900 | 8.28E-08 | 3.00E-08 | 1 | 4 | 0.33 | 0.44 | AABR07014823.1 |

| | | | | | | | | | | | | |
|---|---|---|---|---|---|---|---|---|---|---|---|---|
| oDMR:63 | 14 | 37885301 | 37887001 | 3500 | 1200 | 2.85E-07 | 1.45E-07 | 54 | 19 | 1.54 | 1.58 | |
| oDMR:64 | 14 | 52481601 | 52481601 | 2500 | 2500 | 2.86E-06 | 3.44E-08 | 10 | 10 | 0.4 | 0.4 | AABR07015338.1 |
| oDMR:65 | 14 | 90749901 | 90749901 | 800 | 800 | 2.46E-06 | 1.31E-06 | 4 | 4 | 0.5 | 0.5 | |
| oDMR:66 | 15 | 27179501 | 27179401 | 1600 | 1200 | 2.90E-06 | 2.24E-06 | 20 | 8 | 1.25 | 0.67 | Tlr11 |
| oDMR:67 | 15 | 103569901 | 103569301 | 200 | 800 | 4.75E-06 | 6.70E-07 | 0 | 6 | 0 | 0.75 | AABR07019388.1 |
| oDMR:68 | 16 | 11702401 | 11701901 | 400 | 900 | 1.91E-06 | 4.74E-07 | 2 | 8 | 0.5 | 0.89 | Grid1 |
| oDMR:69 | 16 | 88818301 | 88818401 | 2500 | 3300 | 2.31E-06 | 5.67E-06 | 19 | 27 | 0.76 | 0.82 | |
| oDMR:70 | 17 | 5613701 | 5612101 | 1400 | 3000 | 3.92E-06 | 8.23E-07 | 25 | 50 | 1.79 | 1.67 | Agtpbp1 |
| oDMR:71 | 18 | 68923001 | 68923101 | 2700 | 1100 | 3.43E-07 | 3.75E-07 | 43 | 21 | 1.59 | 1.91 | AABR07032503.1 |
| oDMR:72 | 18 | 85771101 | 85771101 | 200 | 200 | 3.89E-06 | 1.42E-06 | 1 | 1 | 0.5 | 0.5 | |
| oDMR:73 | 19 | 10363901 | 10362501 | 900 | 3900 | 4.14E-06 | 5.08E-07 | 10 | 78 | 1.11 | 2 | Drc7;Katnb1 |
| oDMR:74 | 20 | 12851401 | 12851401 | 300 | 300 | 1.21E-06 | 5.52E-06 | 6 | 6 | 2 | 2 | Lss |
