# Supplementary material for: Atrazine induced epigenetic transgenerational inheritance of disease, lean phenotype and sperm epimutation pathology biomarkers
Source: PLoS One. 2017 Sep 20;12(9):e0184306. doi: 10.1371/journal.pone.0184306 (PMC5606923; doi:10.1371/journal.pone.0184306)

Supplemental Figure S4. F1, F2, F3 Pathways in Cancer

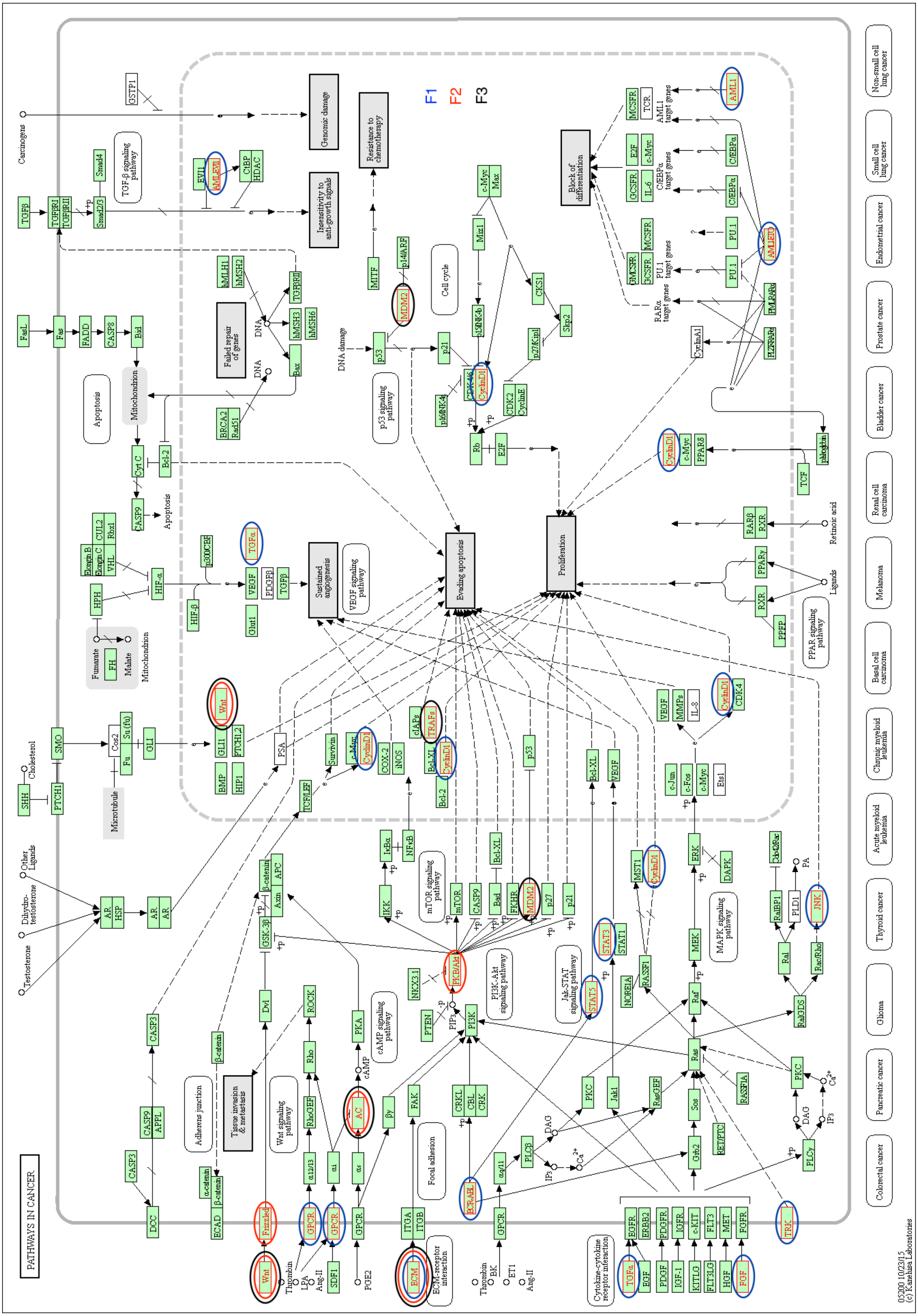

Supplement: S4 Fig — Circled in blue (F1), red (F2) or black (F3) DMR associated genes. (PDF) [file pone.0184306.s004.pdf]
